# Leptospirosis in Aotearoa New Zealand: Protocol for a Nationwide Case-Control Study

**DOI:** 10.2196/47900

**Published:** 2023-06-08

**Authors:** Shahista Nisa, Emilie Vallee, Jonathan Marshall, Julie Collins-Emerson, Polly Yeung, Gerard Prinsen, Jeroen Douwes, Michael G Baker, Jackie Wright, Tanya Quin, Maureen Holdaway, David A Wilkinson, Ahmed Fayaz, Stuart Littlejohn, Jackie Benschop

**Affiliations:** 1 Molecular Epidemiology and Public Health Laboratory School of Veterinary Science Massey University Palmerston North New Zealand; 2 EpiCentre School of Veterinary Science Massey University Palmerston North New Zealand; 3 School of Mathematical and Computational Sciences Massey University Palmerston North New Zealand; 4 School of Social Work Massey University Palmerston North New Zealand; 5 School of People, Environment and Planning Massey University Palmerston North New Zealand; 6 Research Centre for Hauora and Health Massey University Wellington New Zealand; 7 Department of Public Health University of Otago Wellington New Zealand; 8 Enteric and Leptospira Reference Laboratory Institute of Environmental Science and Research Christchurch New Zealand; 9 Goodfellow Unit University of Auckland Auckland New Zealand; 10 College of Health Massey University Palmerston North New Zealand; 11 Unité Mixte de Recherche, Animal, Santé, Territoires, Risques et Ecosystèmes, Centre de coopération internationale en recherche agronomique pour le développement, Institut national de la recherche agronomique University of Montpellier Plateforme Technologique Cyclotron Réunion Océan Indien Sainte-Clotilde, La Réunion France

**Keywords:** leptospirosis, Leptospira, case-control study, New Zealand, mixed methods, protocol, Indigenous health, public health, One Health

## Abstract

**Background:**

In Aotearoa New Zealand, 90% of patients with notified leptospirosis (a zoonotic bacterial disease) have been men working in agricultural industries. However, since 2008, the epidemiology of notified cases has been gradually changing, that is, more women are affected; there are more cases associated with occupations traditionally not considered high risk in New Zealand; infecting serovars have changed; and many patients experience symptoms long after infection. We hypothesized that there is a shift in leptospirosis transmission patterns with substantial burden on affected patients and their families.

**Objective:**

In this paper, we aimed to describe the protocols used to conduct a nationwide case-control study to update leptospirosis risk factors and follow-up studies to assess the burden and sources of leptospirosis in New Zealand.

**Methods:**

This study used a mixed methods approach, comprising a case-control study and 4 substudies that involved cases only. Cases were recruited nationwide, and controls were frequency matched by sex and rurality. All participants were administered a case-control questionnaire (study 1), with cases being interviewed again at least 6 months after the initial survey (study 2). A subset of cases from two high-risk populations, that is, farmers and abattoir workers, were further engaged in a semistructured interview (study 3). Some cases with regular animal exposure had their in-contact animals (livestock for blood and urine and wildlife for kidney) and environment (soil, mud, and water) sampled (study 4). Patients from selected health clinics suspected of leptospirosis also had blood and urine samples collected (study 5). In studies 4 and 5, blood samples were tested using the microscopic agglutination test to test for antibody titers against *Leptospira* serovars Hardjo type bovis, Ballum, Tarassovi, Pomona, and Copenhageni. Blood, urine, and environmental samples were also tested for pathogenic *Leptospira* DNA using polymerase chain reaction.

**Results:**

Participants were recruited between July 22, 2019, and January 31, 2022, and data collection for the study has concluded. In total, 95 cases (July 25, 2019, to April 13, 2022) and 300 controls (October 19, 2019, to January 26, 2022) were interviewed for the case-control study; 91 cases participated in the follow-up interviews (July 9, 2020, to October 25, 2022); 13 cases participated in the semistructured interviews (January 26, 2021, to January 19, 2022); and 4 cases had their in-contact animals and environments sampled (October 28, 2020, and July 29, 2021). Data analysis for study 3 has concluded and 2 manuscripts have been drafted for review. Results of the other studies are being analyzed and the specific results of each study will be published as individual manuscripts..

**Conclusions:**

The methods used in this study may provide a basis for future epidemiological studies of infectious diseases.

**International Registered Report Identifier (IRRID):**

DERR1-10.2196/47900

## Introduction

### Background

Leptospirosis is a neglected zoonotic disease that causes severe febrile illness, renal and hepatic failure, and death [1]. Globally, 1.03 million cases and 58,900 deaths occur annually [2], with a loss of 2.90 million disability-adjusted life years [3]. However, the burden is underestimated [4]. Adding to the disease burden, approximately 30% of patients have symptoms that persist for several years [5,6].

The disease is caused by bacteria of the genus *Leptospira*, which includes >71 species [7] and >300 serovars worldwide [8]. Pathogenic species of *Leptospira* colonize the kidneys of mammals, as well as birds, amphibians, and reptiles [9], and this leads to intermittent urinary shedding. High-risk populations vary spatially and temporally and include those in contact with infected animals (livestock or wildlife) or contaminated environments (water, mud, or soil) [4].

In Aotearoa New Zealand, leptospirosis is a notifiable disease, and its incidence peaked in 1971 with dairy farmers, abattoir workers, and pig farmers identified as a high-risk population [10]. At that time, cattle were the recognized maintenance hosts for serovar Hardjo type bovis and pigs for serovars Pomona and Tarassovi [10,11]. In the early 1980s, cattle vaccines against serovars Hardjo type bovis and Pomona and pig vaccines against serovars Pomona and Tarassovi were developed, and vaccination programs were implemented. Guidelines on the operation of dairy farms were developed, including minimum approved distances between livestock, pigs, and poultry [12]. Awareness campaigns resulted in the increased use of personal protective equipment (PPE) among agricultural workers. Although the guidelines, vaccinations, and PPE appeared to substantially reduce the disease risk in humans [13,14], these preventative measures are only partially effective. Leptospirosis animal vaccines offer strain-specific protection; however, current vaccines do not cover all strains, and noncommercial farmers are often not aware of industry guidelines. In addition, the use of protective equipment does not necessarily prevent infection [15].

Analysis of notification data from 1999 to 2017 showed a decline in leptospirosis cases attributed to serovars Hardjo type bovis and Pomona but an increase in cases attributed to serovars Ballum and Tarassovi [16]. From 1999 to 2017, leptospirosis notifications in abattoir workers were almost exclusively attributed to serovars Hardjo type bovis and Pomona, whereas leptospirosis notifications in dairy farmers were predominantly attributed to serovars Hardjo type bovis and Tarassovi. Occupationally acquired leptospirosis in New Zealand is covered by the Accident Compensation Act 2001. The Accident Compensation Corporation is the Crown entity responsible for enactment of the provisions set out under this Act and manages personal injury including economic, social, and personal costs [17]. However, workplace compensation for leptospirosis cases is challenging for some claimants because of reliance on serological testing and the limited range of automatically eligible occupations [18].

Notification data from 1999 to 2017 also showed that serovar Ballum cases were largely associated with occupations that were not agriculturally based [19]. Our pilot work suggests that wildlife sources [20] and environmental pathways [21] may be increasingly important in disease transmission. A longitudinal survey of wildlife on a coastal dairy farm and bordering forest conducted in autumn and spring for 2 years (from spring 2016 to autumn 2018) determined that the overall prevalence of serovar Ballum in mice, rats, and hedgehogs was 46% (95% CI 39%-52%); 44% (95% CI 26%-62%); and 27% (95% CI 11%-50%), respectively [22]. Metabarcoding of 24 enriched environmental cultures from water, soil, and mud samples taken from the same study site identified pathogenic *Leptospira* in all cultures [21].

### Objectives

On the basis of the changes in serovar predominance in the at-risk occupations, as well as an increase in cases with occupations that are not agriculturally based, we hypothesize a change in the epidemiology of leptospirosis in New Zealand. This identified the need to update risk factors and sources of infection, the protocol of which is outlined in this manuscript. The overall aim of this research is to improve the evidence base to inform effective policies and practices to lower the incidence, health impact, and burden of leptospirosis in New Zealand. The speciﬁc aims are as follows:

1. Assess risk factors for leptospirosis in New Zealand, with a focus on activities associated with livestock, wildlife, and pets, as well as environmental and recreational exposure.
2. Assess the effect of preventative measures such as the use of PPE, animal vaccinations, and hygiene practices during high-risk activities.
3. Describe the burden of the disease including the duration, frequency, and severity of symptoms, costs incurred, and support needed by patients.
4. Explore the barriers associated with workplace compensation for occupationally acquired leptospirosis.
5. Assess the potential sources of infection from in-contact animals and environments.
6. Identify the strains of
  Leptospira
  causing disease in New Zealand.
7. Establish a cohort of patients for a long-term follow-up study.

## Methods

### Study Design

This manuscript describes the design and methods of the study titled, “Emerging sources and pathways for leptospirosis—a paradigm shift,” an investigator-initiated project that was peer reviewed and funded by the Health Research Council of New Zealand (Multimedia Appendices 1-4).

The key study design is a nationwide frequency-matched case-control study with 4 substudies involving cases only. Studies comprise questionnaire-based investigations (studies 1 and 2), semistructured interviews with a few open questions (study 3), and laboratory-based investigations with testing in our research laboratory (studies 4 and 5):

- Study 1: Case-control survey for all participants (aim 1)
- Study 2: Follow-up survey of all cases (aims 2-4)
- Study 3: Semistructured interviews with a subset of cases who worked in agricultural industries at the time of diagnosis (aims 2-4)
- Study 4: Serological investigations of blood and molecular investigations of blood, urine and kidney samples from in-contact animals and environments of a subset of cases who had regular animal and environmental exposure at the time of diagnosis (aim 5)
- Study 5: Serological and molecular investigations of blood and urine samples from a subset of patients suspected of leptospirosis (aim 6)

### Study Location and Period

This is a nationwide study in which the participant recruitment was expected to last for 18 months with the aim of recruiting 150 notified cases starting from July 22, 2019. However, the recruitment period was extended until January 31, 2022, as the New Zealand government–imposed restrictions to manage the COVID-19 pandemic [23] slowed the recruitment process. The extension of the recruitment period allowed an adequate number of cases to be recruited to have sufficient statistical power.

### Cases

#### Case Definition

The case definition for those recruited between July 22, 2019, and October 14, 2020, was the Ministry of Health definition for a confirmed or probable case, that is, a clinically compatible illness with laboratory evidence from a diagnostic laboratory [24].

The case definition given in Textbox 1 was subsequently modified for 2 reasons that became apparent during the conduct of study 5. First, the microscopic agglutination test (MAT) requires 2 blood samples, an acute and a convalescent sample, 2 to 3 weeks apart [25], and 77% (10/13) of the patients did not return to provide the convalescent sample, despite consent to enrollment in the study. Most serological samples are prescreened with an immunoglobulin M (IgM) test and forwarded for MAT only if IgM is positive. Given the case number attrition associated with paired blood samples, case definitions were modified to include those that were IgM positive or equivocal at diagnostic laboratories. This occurred from October 15, 2020, until study completion. Second, 9 of the 10 patients suspected of leptospirosis that did not return to provide the second serology sample, tested positive by the polymerase chain reaction (PCR) analysis performed in the research laboratory. Thus, from January 28, 2021, until study completion, the case definition was further broadened to include cases who tested positive for the tests listed in Textbox 1, and the tests were performed at the research laboratory at Massey University.

**Laboratory evidence for confirmed or probable case as per the Ministry of Health definition.**

Confirmed case

- Isolation of leptospires from a clinical specimen
- Detection of leptospiral DNA from a clinical specimen using the polymerase chain reaction
- Detection of a ≥4-fold rise in serology titers between acute and convalescent sera using the microscopic agglutination test (MAT)
- Detection of a single raised titer of ≥400 using MAT

Probable case

- Detection of a single raised titer of <400 using MAT

#### Case Selection

**Figure 1:**
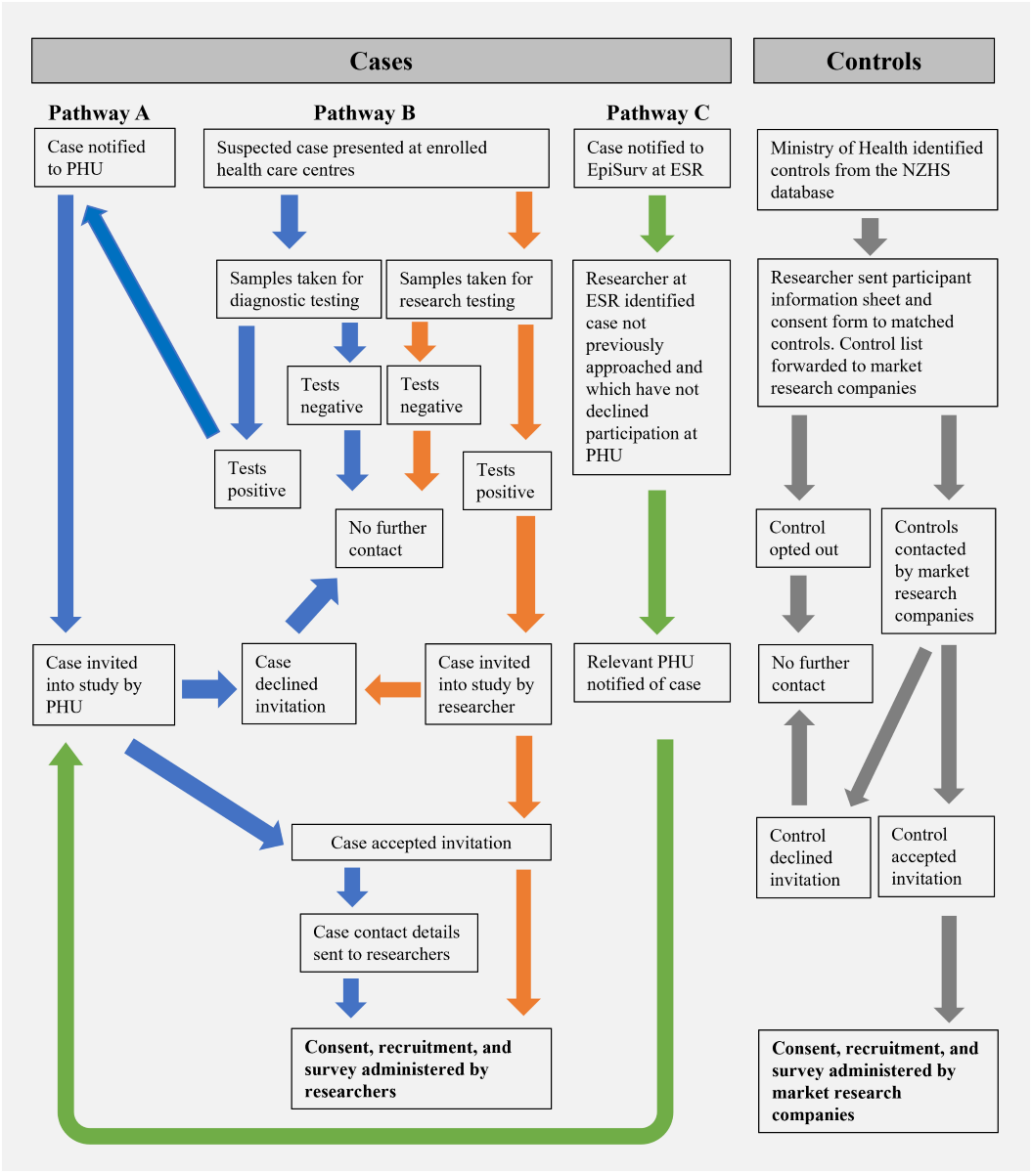
Study flow diagram of participant selection and recruitment for a case-control study on risk factors for leptospirosis in New Zealand. ESR: Environmental Science and Research; NZHS: New Zealand Health Survey; PHU: Public Health Unit.

Cases were identified via 3 pathways (A, B, and C) to ensure that all notified cases were given an opportunity to participate in the study (Figure 1).

1. Notified leptospirosis cases were identified by 12 Public Health Units that represented 20 District Health Boards in New Zealand. Public health officers called all leptospirosis cases to capture demographic and epidemiological data as part of their routine surveillance program for notifiable diseases. This call usually occurred 2 to 4 weeks from when cases first became ill, but the exact time was dependent on the diagnostic tests requested by the attending clinician [26]. Cases who were diagnosed by MAT were called nearer to 4 weeks or later after first becoming ill, because MAT requires 2 samples 2 to 3 weeks apart. At the end of their routine surveillance calls, public health officers informed cases of the study and asked for consent to share contact details with the research team. Cases who did not wish to participate were noted to ensure that they were not contacted again about the study via pathway C.
2. Patients suspected of leptospirosis were identified by clinicians at selected North Island health care facilities. Health care facilities were sampled for convenience based on prior links that had been established between researchers and facility staff and because they were based in regions with a high incidence of leptospirosis, that is, Northland, Waikato, and Hawke’s Bay [16]. To increase recruitment of Māori cases (New Zealand’s Indigenous population), from January 20, 2021, until the end of the study, an additional health care facility in Hawke’s Bay where 76% of service users are Māori was included. Clinicians informed the patients of the study at the time of suspicion of leptospirosis and sought consent to share the patients’ contact details and samples with the research team (Multimedia Appendix 5). A scanned copy of the written consent form was kept with patient notes at the clinic, and the original written consent form was sent to the researchers with the patient’s samples. In addition to the research samples, samples from these patients were sent to the diagnostic laboratories for standard testing.
3. Recruitment of notified cases from pathway A and suspected cases from pathway B was complemented by monthly check-ins with EpiSurv [27], the notifiable disease surveillance database managed by the Institute of Environmental Science and Research on behalf of the Ministry of Health. These check-ins identified notified cases from pathway A who had not already declined to participate and who were not already enrolled and suspected patients from pathway B who met the case definition after diagnostic testing. The local Public Health Units were informed of these cases and requested to contact them to invite them to participate in the study.

#### Case Consent and Recruitment

All consenting cases from pathways A, B, and C who met the case definition were sent a participant information sheet and consent form (Multimedia Appendix 6) via either post or email. The researchers called the cases over telephone at least 1 week after sending the information sheet to go through it. Once the researchers had answered cases’ queries, they requested and documented verbal consent from them for the following parameters:

- Participation in a
  case-control survey
  follow-up survey 6 months after the case-control survey
  semistructured interview if cases worked in an agricultural industry
- Access to cases’
  physician’s notes associated with leptospirosis
  leptospirosis diagnostic test results
  diagnostic DNA samples
- Sampling cases’ in-contact animals and environments if cases had regular animal exposure that was not at an abattoir
- Keeping cases’ contact details to invite them for the long-term follow-up study

#### Case Exclusion Criteria

All participants who were aged <16 years were excluded from the study.

### Controls

#### Control Definition

Frequency matching of controls was considered a suitable approach to reasonably ensure that cases and controls were drawn from the same source population. The frequency was based on the distribution of sex and rurality of leptospirosis notifications from January 1, 2014, to December 31, 2018 [27]. During this time, 89.2% (403/452) of cases were males and 63.6% (204/321) lived rurally. However, only 16.29% (766,060/4,699,755) of the New Zealand population lives rurally [28]. Thus, controls were frequency matched as 90% male and 65% rural dwelling, and a 2:1 ratio of controls to cases was planned. Rurality was determined by home address according to the most recent urban or rural classification method by Stats New Zealand [29].

#### Control Selection

The control population was selected from a database of 18,954 New Zealand Health Survey (NZHS) [26] participants who agreed to be approached for future surveys (Figure 1). This database from the 2016-2017 and 2017-2018 survey periods was provided by the Ministry of Health [30]. The NZHS participants included the usually resident population, that is, they had lived in New Zealand for at least 1 year, with a residence visa or citizenship. Exceptions included the prison population, those who live on small New Zealand islands, and people who have been away from their households for >4 weeks during the NZHS recruitment period.

#### Control Consent and Recruitment

An invitation letter endorsed by the Ministry of Health and Massey University, together with the participant information sheet and consent form (Multimedia Appendix 7), was posted by the researchers to 1715 NZHS participants that matched our sex and rurality criteria. There was an option to opt out by contacting the research team if they desired. A total of 1613 people who received the invitation for the study and did not opt out of the study had their details sent to the market research companies, Up Market Research and Infield International, who conducted the control surveys.

The market research companies contacted potential controls 6 times during the study period via telephone, verified control eligibility, and obtained verbal consent for participation in the study. Controls were only invited to participate in a telephone survey for the case-control survey (study 1).

#### Control Exclusion Criteria

The NZHS participants who were aged <16 years and who had an influenza-like illness in the 4 weeks before the control survey were excluded.

### Substudy Populations

#### Study 2: Selection of Cases for the 6-Month Follow-up Survey

All cases were invited to complete a follow-up questionnaire at the time of the case-control survey. The follow-up survey of cases was planned to occur at least 6 months after the case-control survey.

#### Study 3: Selection of Cases for the Semistructured Interview

Occupational data were collected from all cases who participated in the case-control survey. Participants identified as working in agricultural industries (specifically abattoir workers and farmers) and who were recruited as cases between July 22, 2019, and July 23, 2021, were selected for semistructured interviews. Māori participants were prioritized because they are proportionally overrepresented in the group of people affected by leptospirosis [16].

#### Study 4: Selection of Cases for In-contact Animal and Environmental Sampling

Animal and environmental exposure data were collected from all cases as part of the case-control survey. Participants identified as having regular non–abattoir-associated animal contact were invited to have their in-contact animals (livestock, pets, and wildlife) and environments sampled.

#### Study 5: Selection of Patients for Sampling

Biological specimens from patients for research testing at Massey University were acquired in two ways.

1. All patients suspected of leptospirosis who were recruited at selected health care facilities (study 5 or pathway B detailed in case selection) provided blood and urine samples to be tested at both diagnostic laboratories and the research laboratory.
2. All cases who met the case definition and agreed to participate in the case-control study provided consent for the researchers to access their diagnostic samples through the diagnostic laboratories. Only DNA extracted from blood, urine, cerebrospinal fluid, and plasma to test for leptospirosis with PCR testing was requested from the diagnostic laboratories.

### Data Sources, Collection, and Storage

#### Overview

Data for this study were sourced from:

- Quantitative telephone interviews using questionnaires (case-control and follow-up)
- Selective transcripts of semistructured interview approved by interviewees
- Physician’s notes
- Biological specimens collected from patients, patients’ in-contact animals, and patients’ in-contact environments. Specimens were processed and tested in the research or diagnostic laboratory.

**Figure 2:**
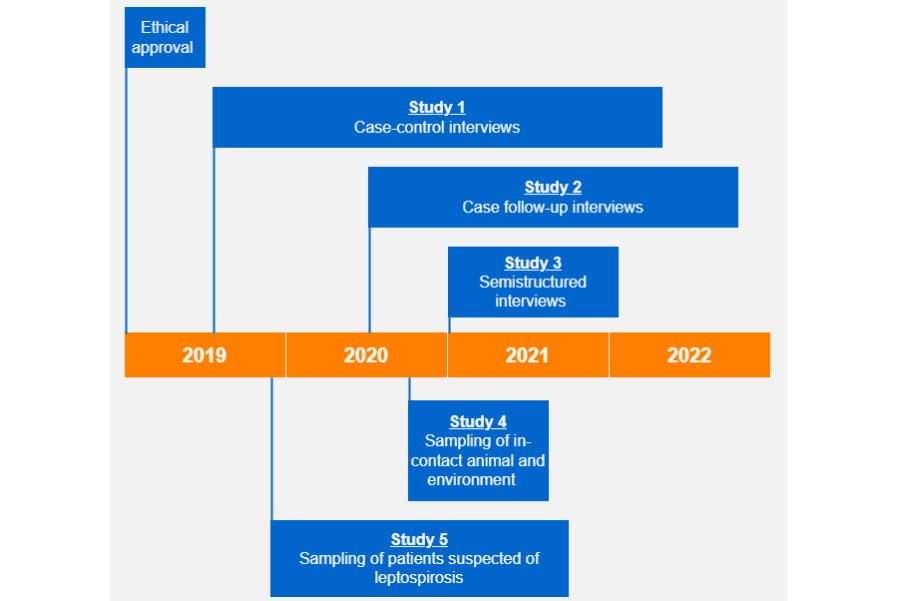
Study timeline of the 5 studies described in this protocol. Cases in study 1 were asked if they were interested in participating in studies 2 to 4 if they met the study selection criteria. Cases took part in study 2 at least 6 months after partaking in study 1. Cases took part in study 3 after they finished study 1 and study 2 interviews. Study 4 was conducted after study 1 at the earliest convenience. Study 5 participants were enrolled from health care facilities upon presentation.

All data were sourced between July 25, 2019, and October 25, 2022 (Figure 2). Study-specific source documents containing identifiable data were maintained either with a restricted access or in a password-protected network shared drive hosted by Massey University. Information pertaining to the identity of the cases was censored from databases and biological samples and was replaced using unique participant codes. Biological samples were stored frozen at −80 °C.

#### Study 1: Case-Control Questionnaire

Eligible cases and matched controls answered a case-control questionnaire delivered over the telephone (Multimedia Appendices 8 and 9). The case-control questionnaire was developed to obtain data on exposures. Care was taken during questionnaire content development, via the review of other studies and questionnaire contents, to ensure that appropriate variables and exposures were incorporated. The questionnaire included the following:

- Leptospirosis case report forms used by the Institute of Environmental Science and Research [27] and the Waikato District Health Board [31]
- Questionnaires used in the study of leptospirosis exposure and risk factors in farmers [32], abattoir workers [33], veterinarians [34], veterinary students [35], and livestock [36] in New Zealand
- Questionnaires used for leptospirosis research in Sri Lanka [37], Nepal [38], and Tanzania [39]
- Case-control questionnaires used for other diseases in New Zealand, including campylobacteriosis [40], acute rheumatic fever [41], Legionnaires’ disease [42], and Shiga toxin–producing *Escherichia coli* [43]

The case questionnaire also contained additional items including clinical course and outcome, sick leave, and sick leave entitlements as well as emotional well-being questions that were followed up in the substudy surveys. The researchers received training on the delivery of sensitive questions such as the Kessler Psychological Distress Scale (K10) [44] and risk assessment and management skills for interviewing sensitive people.

The case questionnaire was pilot tested by the researchers in 3 cases (patients who had leptospirosis before this study commenced). After piloting, changes were made to the survey questions to remove ambiguities.

Case interviews were conducted by the research team from July 25, 2019, to April 13, 2022, at a time that suited the participants and as soon as convenient after the initial contact. All cases were asked about their exposures in the month before they became ill. Data were collected using the electronic version of the questionnaire and transferred to the database, and 10% of the surveys were checked for data integrity.

All control interviews were conducted by the market research companies: UMR Market Research between October 19, 2019, and February 28, 2021, and Infield International between May 1, 2021, and January 26, 2022. The control questionnaire was pilot tested by the UMR Market Research on 12 controls (participants of the NZHS). The control surveys were conducted in 6 batches, with 50 controls interviewed in each batch. Batched control interviews were conducted in October and November 2019; January and February 2020; January and February 2021; May 2021; August 2021; and January 2022. The lack of control interviews between March 1, 2020, and December 31, 2020, was due to a delay in research during the COVID-19 pandemic. All controls were asked about their exposures in the month preceding the interviews. Data from the market research companies were received in emails with password-protected Microsoft Excel spreadsheets and transferred to the database, and 10% of the surveys were checked for data integrity.

#### Study 2: Follow-up Questionnaire

The follow-up questionnaire (Multimedia Appendix 10) was designed to expand on some of the questions from the case-only items in the case questionnaire and to introduce some new items. Follow-up interviews were conducted between July 9, 2020, and October 25, 2022. All symptoms identified in the case questionnaire were further quantified with symptom duration, frequency, and severity scores of 1 to 5, where 1 was mild and 5 was severe. Participants were also asked if they had any new symptoms that they attributed to leptospirosis since their previous interviews. Other questions included following up on current occupation, updates on sick leave, sick leave entitlements, and a repeat of the K10 questions. New items included the costs associated with leptospirosis treatment and support from the workplace, family, and friends during the illness. The follow-up interviews were administered to the cases at least 6 months after the case-control interview and by the same research team. The follow-up questionnaire was pilot tested by the researchers on 4 people who had leptospirosis before this study commenced. Data were collected using the electronic version of the questionnaire and transferred to the database, and 10% of the surveys were checked for data integrity.

#### Study 3: Semistructured Questionnaire

The semistructured questionnaire (Multimedia Appendix 11) was designed to collect patient experience data to inform the work of 3 groups of professionals: medical professionals (doctors and nurses); public health policy makers (Ministry of Health and Accident Compensation Corporation); and stakeholders in agricultural industries (managers, workers, industry boards, and workers’ unions). Semistructured interviews were conducted between January 26, 2021, and January 19, 2022. Cases were asked to share their experiences of the disease; the financial implications; and their advice to workmates, employers, and their community. The semistructured interviews were conducted by the research team that received speciﬁc training for this interview. The semistructured questionnaire was pilot tested by the researchers on other researchers in the team who role-played as patients. This helped the researchers to upskill their rapport-building and prompting skills.

Face-to-face interviews were carried out before the COVID-19 pandemic–related movement restrictions were implemented, after which distance interviews using telephone or video calling were used. Data were recorded using Dictaphone for in-person interviews or Zoom (Zoom Video Communications) for distance interviews. Selective transcripts were made from the recordings and sent to the respective interviewees for review and editing with a request to return the transcript within 2 weeks.

#### Study 4: In-contact Animal and Environmental Sampling

##### Farm Animal Samples

All farm animal samplings were conducted between October 28, 2020, and July 29, 2021. All live animals were sampled for blood and urine by either the case’s veterinarian or a veterinarian serving the area the farm was in. When animals were culled as part of normal farming practices (eg, prime cattle), kidney samples were also collected from the abattoir. On the basis of our earlier studies on leptospirosis in animals, up to 40 livestock of each relevant species were sampled; this allowed the detection of *Leptospira* infection or exposure for a prevalence as low as 15% for any herd or flock size with an error of 5% [45,46] and the isolation of leptospires, assuming a shedding prevalence of 35% and a success rate for isolation of 20% [47].

- Cattle were sampled while restrained in a race or milking shed. Cattle serum was collected from the jugular vein or tail vein with a 20 g needle with a volume not exceeding 10 mL. Urine from cattle was either an opportunistic free catch or collected by perivulval stimulation.
- Sheep were sampled while restrained by hand in a race or a yard. Sheep serum was collected from the jugular vein with a 20 g needle, with the volume not exceeding 10 mL. Urine from sheep was opportunistic free-catch urine or collected by a partial smothering technique. The partial smothering time was restricted to 15 seconds.
- Dogs were sampled while they were restrained by hand. Dog serum was taken from the cephalic or jugular vein with 23 to 22 g needles with the volume not exceeding 5 mL. Urine collection in dogs was by free catch, with an option for the veterinarian to use diuretics.

The samples were sent chilled to the laboratory at Massey University. Approximately 100 µL of urine was cultured in Ellinghausen-McCullough-Johnson-Harris (EMJH) medium, and cultures were kept at 28 °C with shaking and monitored for 13 weeks [48] before being discarded. The rest of the urine was centrifuged at 3000g for 20 minutes, with the supernatant removed [45], and the pellet stored at −80 °C and batch tested. All kidney samples were homogenized in an equal weight/volume of phosphate-buffered saline (PBS); the slurry was then centrifuged at 3000g for 20 minutes, and most of the supernatant was removed, leaving behind 1 mL of liquid [47], which was stored at −80 °C and batch tested. All serum samples were centrifuged at 3000g for 20 minutes, and the supernatant was stored at −80 °C and batch tested [49].

##### Wildlife Samples

Attempts were made to capture wildlife from farms that provided farm animal samples if the farmers were able to support this work. Wildlife was captured in kill traps, and necropsies were performed by the farmers who were provided with instructions by the researchers. Both kidneys were collected, bagged separately, and frozen upon collection by the farmer. The kidneys were then sent to the research laboratory at Massey University in 1 batch. Kidneys were thawed upon receipt and homogenized in equal weight/volume of PBS; the slurry was centrifuged at 3000 g for 20 min, and most of the supernatant was removed, leaving behind 1 mL of liquid that was stored at −80 °C and batch tested.

##### Environmental Samples

Attempts were made to obtain environmental samples from the farms that provided farm animal samples. Where possible, 10 sites (eg, farm dam, muddy paddock) that the participants identified as having contact with during the month before they became ill were sampled. Participants were sent materials and methods outlining how to collect environmental samples from the identified sites. Where possible, a 50-mL specimen bottle was filled with soil, mud, and water from each site. All samples were sent to the research laboratory at Massey University at room temperature and processed within 3 days of receipt. The soil and mud samples were resuspended in 50 mL of PBS, and the supernatant was collected for processing. Soil and mud supernatant and water samples were centrifuged at 3000g for 20 minutes, and most of the supernatant was removed, leaving behind 1 mL of liquid of which 50 µL was cultured in EMJH culture medium, while the rest was stored at −80 °C and batch tested with PCR [21].

#### Study 5: Human Samples

Samples from patients suspected of leptospirosis were obtained at the patients’ usual phlebotomy centers between November 6, 2019, and September 29, 2021. Research samples were collected at the same time as the diagnostic samples and included whole blood, serum, and urine samples. Approximately 100 µL of whole blood and urine was inoculated into the EMJH medium at the laboratories serving the phlebotomy centers. The culture medium and culture protocol were provided to these laboratories by the researchers as part of this study. The rest of the urine was centrifuged at 3000g for 20 minutes; the supernatant was removed, and the pellet was resuspended in RNAlater (Ambion). A total of 5 tubes containing blood culture, urine culture, serum, whole blood, and urine pellets in RNAlater were sent to the research laboratory at Massey University at room temperature. Blood and urine cultures were kept at 28 °C with shaking and monitored for 13 weeks before being discarded. Serum samples were centrifuged at 3000g for 20 minutes, and the supernatant was kept at −80 °C and batch tested. The urine pellet and whole blood were kept at −80 °C and were batch tested. Diagnostic laboratory results for all notified cases in the study were collected from EpiSurv to identify cases that were tested with a diagnostic PCR and to identify the diagnostic laboratories that ran these PCRs. The researchers then acquired the diagnostic sample (DNA) from the respective diagnostic laboratories to perform a research PCR.

### Ethics Approval

This study received human ethics approval from the Health and Disability Ethics Committee (reference number 19/STH/80). In addition, this study received locality agreements together with local Māori consultations from 20 District Health Boards. The study received animal ethics approval from the Massey University Animal Ethics Committee (reference number Protocol 19/11).

### Data Analysis Plan

#### Sample Size and Statistical Power

For common exposures (prevalence 30%-70%, such as exposure to flooded paddocks) [35], 150 cases and 300 controls provide more than 80% power for odds ratios (ORs) as low as 1.8, whereas for less common exposures (15%, such as wild deer on or near the farm) [32], this provides 80% power to detect ORs as low as 2.1.

#### Missing Data

Our approach to handling missing data was to design the answers to the survey questions with contingencies. For sensitive questions such as date of birth, participants could either provide their date of birth, age, or an age range. In addition, the category *unsure* was available to interviewers if participants were asked about the vaccination status of the animals they worked with or the treatment of their work water supply, as employees may not necessarily have this information. The *unsure* category was included in the data analysis.

#### Study 1: Risk Factors

Descriptive statistics consisted of comparing cases and controls (eg, percentage of respondents within strata and their CIs) according to the demographics and exposure factors present in the questionnaire. Contingency tables were drawn to compare dichotomous and ordinal variables. When applicable, 2 × 2 tables and Fisher exact test were used to calculate the crude ORs and CIs. Ordinal, discrete, and continuous variables with nonnormal distributions were compared using the nonparametric Mann-Whitney-Wilcoxon rank sum test. Boxplots were used to compare discrete and continuous variables. Comparison of independent variables and collinearity was determined using Pearson correlation coefficients. Timelines were plotted to analyze temporal trends and compare interviews of cases and controls.

Multivariable logistic regression models were used to calculate the ORs and CIs. Both crude and adjusted ORs were calculated for each exposure, with adjustment for potential confounders and matching variables. Variables were added in a stepwise manner forming precursor models; this was done separately for each main category of exposure variables, for example, wildlife. Subsequently, the different category precursor models were combined before running a further stepwise backward selection. To assist the decision of variable inclusion or exclusion, the following methods were used: relevance of the exposure in the literature and expert opinion, the Akaike Information Criterion of the model, and sensitivity analysis. Risk factors were initially estimated from a model without occupation, while adjusted for the matching factors (rurality and sex) and other confounders. To provide insight into the effect of occupation, a second analysis was conducted with occupation included.

#### Study 2: Follow-up of Cases

The proportion of cases with different symptoms at several time points after the onset of symptoms and up to the time of the follow-up interview was calculated. Kaplan-Meier survival curves were used to graphically display the time until symptoms were no longer present [50]. Multivariable logistic regression models were used as described for the analysis of risk factors to identify possible factors associated with the persistence of symptoms, and crude and adjusted ORs and 95% CIs were calculated.

Descriptive analyses using means, frequencies, and percentages were used to explore how living with leptospirosis had impacted patients’ activity participation, ability to cope, capability to return to usual daily activities, and support received from family and friends for recovery.

The K10 scores will be categorized using the method described in the NZHS [51]. Each of the 10 questions was scored from 0 to 4, and for NZHS reporting, psychological distress meant having a score of ≥12. This analysis will allow case K10 scores at both time points from the acute and follow-up surveys to be compared with those of NZHS participants. To assess changes in K10 scores over time, a mixed model repeated measures ANOVA will be used as described previously [52].

#### Study 3: Semistructured Interviews of Cases

Anonymized selective transcripts were made from the recordings of the semistructured interviews by the interviewer, leaving in what the interviewer considered to be the essential answers to inform the work of 3 groups of professionals. Selective transcripts were used as raw data for content analysis [53]. In this analysis, 3 important choices were made. First, the content analysis focused on *what was said* without the exploration of *what was meant*. Second, the content analysis was inductive, and recurring themes were identified as they emerged from the transcripts. Finally, an issue was considered a recurring theme when approximately one-third of the interviewees mentioned it [54-56].

#### Studies 4 and 5: Sources of Infection

Attempts to identify the strains of *Leptospira* in human, animal, and environmental samples were as follows:

- Genotyping pure *Leptospira* cultures: DNA extractions from cultures were subjected to *LipL32* quantitative PCR [57] and *glmU* conventional PCR [21] for the detection of pathogenic *Leptospira*. All *glmU* PCR amplicons were sequenced to identify the species and serovars of *Leptospira*. Any culture-negative samples for the pathogenic PCRs were subjected to a 16s PCR [58] to identify saprophytic or intermediate strains of *Leptospira.*
- Genotyping samples: all samples stored at −80 °C from humans (blood, urine, cerebrospinal fluid and serum); animals (urine and kidneys); and the environment (water, soil, and mud) were subjected to DNA extraction and to *LipL32* and *glmU* PCRs as described in the previous paragraph. Samples positive for *glmU* PCR were sent for amplicon sequencing to identify species and serovar of *Leptospira*.
- Serotyping via MAT: all serum samples were serially diluted from 1/24 to 1/3072 and subjected to a MAT assay against 5 serovars belonging to 2 species: *Leptospira borgpeterseneii* serovars Hardjo type bovis, Ballum, and Tarassovi and *Leptospira interrogans* serovars Pomona and Copenhageni. Seropositivity for humans was defined as per the case definition for cases (Textbox 1). Seropositivity for animals was defined as a MAT titer of ≥48, whereas titers of ≥384 indicated likely current or recent infection [59].

Culture media, cultivation of cultures, DNA extraction method, primers, and PCR protocols were as published previously [21].

## Results

All participants in this study were recruited between July 22, 2019, and January 31, 2022, and data collection for the study has completed. During this period, a total of 220 cases were notified to ESR, of which 139 were forwarded to us from the Public Health Units; 12 (8.6%) of whom declined to participate, 24 (17.3%) did not meet the case definition, and 7 (5%) were unable to be contacted; thus, 95 (68.3%) cases met the case definition, agreed to participate in the study, and were interviewed. All case interviews were conducted from July 25, 2019, to April 13, 2022. A total of 300 controls were recruited and interviewed: 150 (50%) from UMR Market Research and 150 (50%) through Infield International. Control interviews were conducted from October 19, 2019, to January 26, 2022. A total of 91 cases participated in the follow-up interviews that were conducted between July 9, 2020, and October 25, 2022, and 13 cases participated in the semistructured interviews that were conducted between January 26, 2021, and January 19, 2022. Semistructured interviews were conducted after the participants completed the case (study 1) and follow-up (study 2) interviews. Data integrity checks showed that all responses to the questions in the case-control and follow-up surveys were recorded because of the contingencies put in place to account for missing data. In-contact animal and environmental samples were collected from 4 farms (1 dairy farm, 1 sheep farm, and 2 beef farms) between October 28, 2020, and July 29, 2021. Samples were collected from cattle (16 kidney, 186 serum, and 193 urine samples); sheep (40 urine and 40 serum samples); dogs (12 serum samples); possums (28 kidney samples); rats (6 kidney samples); wild pigs (3 kidney samples); wild rabbits (3 kidney samples); and environmental sources including water (n=10), mud (n=11), and soil (n=15). A total of 20 samples were collected from patients suspected of leptospirosis between November 6, 2019, and September 29, 2021.

Data analysis for the semistructured interviews has concluded, and 2 manuscripts have been drafted for review. Results of the other studies are being analyzed and the specific results of each study will be published as individual manuscripts. All animal and human samples have been tested, and the results returned to farmers and patients by their veterinarians or clinicians, respectively.

## Discussion

### Principal Findings

This study is the first in New Zealand to quantify the association between leptospirosis and a range of risk factors including contact with livestock, wildlife, and pets; exposure to soil, mud, and water; and the use of PPE and animal vaccination. The identification of risk factors will enable us to design effective intervention strategies to reduce exposure to these factors and thus reduce the disease burden in New Zealand (study 1). Our assessment of the disease burden for patients is novel, as we included the potential difficulties associated with compensation for occupationally acquired cases. This information may be used to update the compensation policies to adequately support future leptospirosis cases (study 2). The successful follow-up survey of 91 cases and 13 cases from agricultural occupations who shared their experiences in a semistructured interview will provide a new and detailed understanding of the postleptospirosis experience and its burden on individuals and households. This information will help identify the appropriate measures, including social, financial, and emotional measures, to support leptospirosis cases (studies 2 and 3). Furthermore, the data from patients’ in-contact animals and environments will provide information on pathways for infection previously not considered important in New Zealand, such as pests or flooding, which will inform (and have informed) health messaging (study 4). Finally, this study identified a large underascertainment of cases, as 70% of patients did not return to provide a second blood sample and did not receive a diagnosis (study 5).

### Limitations

It is important to acknowledge the limitations of this study. Case-control studies are generally prone to biases, particularly differential recall, and selection biases. To reduce the impact of recall bias on the findings of this study, both cases and controls were given similar memory aids during the interview and close-ended questions were prioritized over open-ended questions at the analysis stage. Selection bias was reduced by matching only 2 variables (sex and rurality), the major known confounders, to ensure that controls were representative of the case population, as 90% of leptospirosis cases were males and 65% lived in rural areas of New Zealand. By focusing on notified cases, albeit with some broadening of the case definition, the issue of underascertainment, which has previously been estimated at 3-fold where mild cases do not seek medical attention and thus do not receive a diagnosis or get notified [60], was not directly addressed; however, the inclusion in our protocol to (1) enroll and test patients suspected of leptospirosis with a range of research tests (culture, PCR, and serology) and (2) test diagnostic samples with a research PCR will shed light on this hidden burden.

### Strengths

One of the strengths of this study was ensuring that all notified cases were given an opportunity to participate in the study by using 3 pathways to identify these cases nationwide (Figure 1). This effort is crucial for a disease uncommon in New Zealand (approximate incidence risk of 2 per 100,000 people) [16] and ensures that the results from this study will provide nationally valid data.

### Future Work

Future research goals include following the cohort of patients established in this study (aim 7) in a long-term follow-up study. Furthermore, the underascertainment of cases owing to the requirement of 2 tests identified in study 5 has led us to collaborate with human diagnostic laboratories to develop a PCR test that will require 1 sample for diagnosis and will subsequently be able to type the strain of *Leptospira*.

### Conclusions

The dissemination of the study results will occur through scientific and stakeholder channels including publications in international peer-reviewed journals and presentations at international and national meetings. Our stakeholders in the community such as the Meat Industry Association, NZ Meat Workers Union, Department of Conservation, Dairy Women’s Network, WorkSafe NZ, Rural Women New Zealand, and district and regional councils will use their websites, social media channels, and publications to deliver key messages. We held a leptospirosis forum in March 2019 that socialized the study with stakeholders and in September 2022 with stakeholders and patients to disseminate the findings of the study. The researchers have summarized the key findings of the study and circulated them to Public Health Units, District Health Board research offices, Māori Health teams, and all participants. These summaries will also be more widely disseminated to the community.

## Appendix

Multimedia Appendix 1 Peer review comments from the Health Research Council of New Zealand—Reviewer #11.

Multimedia Appendix 2 Peer review comments from the Health Research Council of New Zealand—Reviewer #48.

Multimedia Appendix 3 Peer review comments from the Health Research Council of New Zealand—Reviewer #73.

Multimedia Appendix 4 Peer review comments from the Health Research Council of New Zealand—Reviewer #97.

Multimedia Appendix 5 Suspected patient information sheet.

Multimedia Appendix 6 Case information sheet.

Multimedia Appendix 7 Control information sheet.

Multimedia Appendix 8 Case questionnaire.

Multimedia Appendix 9 Control questionnaire.

Multimedia Appendix 10 Follow-up questionnaire.

Multimedia Appendix 11 Semistructured questionnaire.

## Abbreviations

EMJH: Ellinghausen-McCullough-Johnson-Harris
IgM: immunoglobulin M
K10: Kessler Psychological Distress Scale
MAT: microscopic agglutination test
NZHS: New Zealand Health Survey
OR: odds ratio
PBS: phosphate-buffered saline
PCR: polymerase chain reaction
PPE: personal protective equipment

## Data Availability

The data sets generated during this study are not publicly available, as they are currently unpublished data. Data sets may be available from the corresponding author when they are published on reasonable request.
