# Supplementary material for: Leptospirosis in Aotearoa New Zealand: Protocol for a Nationwide Case-Control Study
Source: JMIR Res Protoc. 2023 Jun 8;12:e47900. doi: 10.2196/47900 (PMC10288348; doi:10.2196/47900)
Supplement: Multimedia Appendix 1 [file resprot_v12i1e47900_app1.pdf]

Applicant peer review report

Reviewer # 11

Proposal details

Title	Emerging Sources and Pathways for Leptospirosis - a paradigm shift
First named investigator	Dr Jackie Benschop (Massey University)

Rationale for Research — Score: 7

There are significant knowledge gaps relating to Leptospirosis not only in New Zealand but also the world. Given the current increase in Leptospirosis notifications in New Zealand it is very prudent to identify changing risk factors, identify infecting species and their sources. Moreover long term follow up has been needed for this disease for decades. I am very encouraged to see this included in the project and fully support such an undertaking.

Design and Methods — Score: 6

The 150 cases and 300 community controls should provide sufficient power to draw solid conclusions. The authors state they would not include those <17 years of age. I would be inclined to include this group however I totally understand that this may make the ethics application and process too burdensome.

Health Significance — Score: 7

This project appears timely given the increase in Leptospirosis notifications in New Zealand. Identifying the ever changing risk factors, the infecting serovars, sources of infection and long term follow up will only improve public health outcomes and reduce the burden on New Zealand's health care system. The importance of understanding the longer term impact of this neglected disease is also vital.

Research Team — Score: 7

The research team is headed by experts as evidenced by publications in international peer review journals, funding, teaching and clinical practice. The research team has extensive extensive experience in Leptospirosis and public health research. There are a number of discipline experts to help guide junior staff.

General comments

Wonderful project that I full endorse. I would encourage the researchers to review their budget for whole genome sequencing. I think $20 000 may be a bit of an underestimation however I am not familiar with the commercial costs of WGS in New Zealand.

Secondly, it would be good to clarify what criteria is used to define a case. Eg.....Are all cases confirmed by an MAT titre of say 400 with a reactive IgM or perhaps a 4 fold rise in MAT titre or culture. I would not be inclined to define a case based solely on a PCR.
