# Supplementary material for: Leptospirosis in Aotearoa New Zealand: Protocol for a Nationwide Case-Control Study
Source: JMIR Res Protoc. 2023 Jun 8;12:e47900. doi: 10.2196/47900 (PMC10288348; doi:10.2196/47900)
Supplement: Multimedia Appendix 2 [file resprot_v12i1e47900_app2.pdf]

Applicant peer review report

Health Research Council of New Zealand

Reviewer # 48

Proposal details

Title	Emerging Sources and Pathways for Leptospirosis - a paradigm shift
First named investigator	Dr Jackie Benschop (Massey University)

Rationale for Research Score: 5

The rationale for this research is that there has been a recent increase in the reported number of cases of leptospirosis and that the implicated serovars may be changing, current vaccines may not cover these serovars, and the number of cases could potentially increase further through rodent and environmental pathways. Increased flooding may be implicated in the increase in cases. The proportion of female cases is also increasing and there may be long term sequelae for cases. The focus on sources of leptospirosis will influence prevention and control mechanisms. The rationale is well presented.

The figure is somewhat hard to read, although an increase can be seen, the size of the oval shape has to be estimated (and its size equates with a different incidence in each time period). Can the researchers provide the data on incidence of serovar, with confidence intervals by year? While there are increases in some areas, others, particularly in the South appear to beshowing decreases.

It was not clear what evidence exists for “accepted workplace harm”- could this be expanded upon?

How much more likely to be hospitalised are those notified with the serovar Ballum as the p-value does not indicate the size of the effect?

Design and Methods Score: 3

A case control study of 150 cases of LS, 300 community controls and a qualitative study of 30 cases and 30 occupationally matched controls are to be undertaken. More detail on these methods would have been useful.

The sampling of cases is well described and the three tiered approach will enhance case ascertainment. Recruitment for tiers 1 and 2 will occur through the medical practitioners and laboratories– will there be a protocol for this? How will data on those attended, approached and consented be collected? What follow-up will there be if there is no initial response (for cases and controls)?

The sampling and recruitment of controls is not described, beyond reference to another study. Are the controls to be randomly selected and how will that occur; what sampling frame will be used? What sampling paradigm will be used- will incidence density sampling occur? Will they be matched on any factors? What processes will be in place to confirm that they do not have LS?

The interviews will be based on that used in the previous study – has that questionnaire been found to be valid and reliable? (I was not able to access the reference cited as it was a conference proceedings.) How will pre-testing occur? Over what time period will exposure, such as domestic animal contact, exposure to flood water, be asked about? Related to this what is the incubation period?

Who will undertake the interviews? Given the additional questions for cases, what strategies are in place to minimise interviewer bias? What is the potential for differential recall bias? Are there any opportunities to validate responses to some questions, e.g, exposure to flooding?

Sampling of contact animals and environment seems ambitious, but is probably feasible.

Re Aim 5, for how long will the cases be followed and how will persistent post LS symptoms be assessed? Will 150 cases be an adequately sized cohort?

The qualitative component is also only described very briefly as semi-structured interviews. In the analyses, what are the recognised and bespoke approaches?

Data analyses- It is stated "the association between LS and putative explanatory variables, including severity of symptoms and infecting serovars, will be assessed initially with multivariable logistic regression, adjusted for the effect of potential confounders." Are severity of symptoms and serovars explanatory variables or actually part of the outcome LS? Will this study have the power to investigate different serovars as outcomes? What are the potential confounders to be considered?

Health Significance — Score: 5

The study potentially will inform strategies for prevention of LS. In particular aim 4 will potentially have an important positive impact for persons with LS.

Because little information is provided on the qualitative component it is not clear what the impact with regard to PPE will be.

Dissemination of results appears comprehensive. What will be the communication strategies for influencing vaccine targets?

Research Team — Score: 5

A comprehensive skill set is covered and members of the team have collaborated previously. The team incorporated capacity building with a PhD student and 2 ECRs . Generally the team have good track records, some excellent and some appropriately reflecting practitioner experience. For example investigator Wright has 20+ years of research experience, but just 12 publications and 5 conference papers. However this 20+ years seems to be in a research laboratory and publications may not have been a focus of this position. The relationship of Prinsen's track record to this grant is less clear.

General comments
