# Supplementary material for: Leptospirosis in Aotearoa New Zealand: Protocol for a Nationwide Case-Control Study
Source: JMIR Res Protoc. 2023 Jun 8;12:e47900. doi: 10.2196/47900 (PMC10288348; doi:10.2196/47900)
Supplement: Multimedia Appendix 3 [file resprot_v12i1e47900_app3.pdf]

# Applicant peer review report

Health Research Council of New Zealand

Reviewer # 73

**Proposal details**

| | |
|---|---|
| Title | Emerging Sources and Pathways for Leptospirosis - a paradigm shift |
| First named investigator | Dr Jackie Benschop (Massey University) |

## Rationale for Research — Score: 6

The rationale for the research is well described and supported by citation of relevant research and other NZ data. While leptospirosis has long been recognised as an occupational hazard in particular NZ industries, its recent increase in incidence in both occupational and non-occupational settings is of concern, as is the emergence of a novel strain and changes in the patterns of isolates from cases in NZ and globally. It is possible that both changes in the wider environment (including the effects of climate change) and human behaviours may be contributing to the recent increase in incidence in the disease and other changes in its epidemiology. The disease has serious acute and long term health impacts, and the issue of barriers to obtaining ACC compensation for occupationally acquired disease is a longstanding one. The long term health impacts of the disease are poorly understood. The aims of the proposed study address some important questions which are of relevance in NZ and also have the potential to add to global knowledge of the disease:

1. What are the drivers of increased incidence of leptospirosis (in terms of changes in risk factors)?

2. What are the prevalent strains of Leptospira and are there species-specific sources for these?

3. What are the long term consequences of leptospirosis?

4. How well does the current ACC system respond to claims for occupationally-acquired leptospirosis.

The proposed study's approach is not necessarily highly original but its strength is in its use of multiple, robust and complementary methods to address the study questions. It will also be novel in using modelling and molecular tools to look at the role of contaminated water in the wider, non-occupational environment as a source for this disease.

## Design and Methods — Score: 5

A prospective case control study is the optimal approach in this situation and the three nested sub studies each address specific questions. While the proposal lacks detail of the case control questionnaire at this point, the process the investigators proposed to develop it is robust and the investigators and their collaborators have the requisite expertise for the task. The investigators have also given considerable thought to recruitment and the process of engagement with medical staff making the diagnosis as well as public health units (PHUs) receiving disease notifications. The study is described as a national one but the measures outlined regarding engagement with clinicians and PHUs are limited to the North Island. The study aims to recruit up to 20 cases from elsewhere in NZ so that if the investigators are successful in being funded, they will need to ensure that PHUs elsewhere in NZ are aware of this study.

The animal and environmental sampling proposed also seem reasonable but are highly dependent on a streamlined and timely process of case identification and interview. This will be challenging.

While I think that the investigators have given a lot of thought to how to maximize the recruitment of cases, I am not as convinced that their strategies for control recruitment, particularly occupationally matched controls will be as successful, even taking into account the strong networks with collaborators in the agricultural sector. The criteria for matching are not clear: will the controls be matched on any other criteria such as age or sex as well as occupation? It is also not clear how these matched controls will be selected or exactly what the sampling frame will be and I would be concerned at the

possibility of bias arising from convenience sampling (of course, if the data are not to be subject to statistical analysis but rather by qualitative analysis then convenience sapling can be justified). The investigators have also not provided a separate power calculation for this particular sub-study but the power will inevitably be lower than for the larger main study. I am also a bit concerned that a study with 30 cases and 30 controls will not be able to determine meaningful and statistically significant differences in the factors described under the heading Workplace Attitude Assessment on page 14. These factors might be better understood by taking a qualitative, rather than quantitative approach and this seems to be what is suggested further down on page 14. It is unclear to me why, if this sub-study is in fact a qualitative study (and it seems that it might be), matched controls are required at all. In my view, this is perhaps the weakest and least well developed part of the application. The study team has very strong human and veterinary epidemiological and laboratory skills but only one member is a social scientist and his contribution is given as 3%.

One of the study's aims is to establish a cohort of cases for long term follow up but exactly how the database of cases will be maintained (including the confidentiality of case data) and the nature and frequency of the follow up are not described at all in the application. While the latter may be the subject of another funding application at a future date, it is still important to be clear at this point what this database/registry will consist of, how the data will protected and who will have access to it (including informed consent from the cases).

## Health Significance **Score: 5**

The investigators have made quite a comprehensive assessment of the known health and economic impacts of leptospirosis in NZ. They have also explained how the components of the proposed study, including both human, animal and molecular epidemiology will advance knowledge relevant to health. A better understanding of the drivers of incidence and burden of disease of leptospirosis in NZ has strong potential to contribute to contribute to improved health outcomes for workers within the primary production sector and more widely as well.

## Research Team **Score: 6**

The research team have a strong track record and a wide range of appropriate research skills. All have been active in publishing their research and other dissemination activities. Collectively, they also have excellent networks within the primary production sector and good relationships with relevant parts of the health sector, including PHUs. The lead investigator has particularly strong relationships with both sectors which will serve to facilitate the conduct of this research and dissemination of the findings. Their international advisory panel is also very well placed to assist the dissemination of the results of the proposed study.

## General comments

In addition to the potential risk factors for the increase in non-occupational disease that the applicants have identified, such as flooding, they may also wish to consider the increasing frequency of human/environment/direct and indirect animal source contacts afforded by freedom camping and outdoor festivals and so-called "Gatherings" held either on agricultural land or within bush areas where participants get in rather closer touch with nature than they might otherwise do. I am aware of at least one recent case (who became seriously ill) who almost certainly acquired the disease having stayed for several days with others in a remote bush location and deliberately going barefoot to get in touch with nature. He was also initially reluctant to admit to this as the "Gathering" he attended was promoted through a closed Facebook network.
