# Supplementary material for: Leptospirosis in Aotearoa New Zealand: Protocol for a Nationwide Case-Control Study
Source: JMIR Res Protoc. 2023 Jun 8;12:e47900. doi: 10.2196/47900 (PMC10288348; doi:10.2196/47900)
Supplement: Multimedia Appendix 4 [file resprot_v12i1e47900_app4.pdf]

Applicant peer review report

Health Research Council of New Zealand

Reviewer # 97

Proposal details

Title	Emerging Sources and Pathways for Leptospirosis - a paradigm shift
First named investigator	Dr Jackie Benschop (Massey University)

Rationale for Research Score: 6

Leptospirosis is an important disease but high quality research is rare. I think the proposed work is highly relevant and is able to make substantial contribution to our understanding of leptospirosis. The combination of epidemiological research, genetics and economic evaluation is extremely powerful and might complement each other quite well.

Design and Methods Score: 5

First, I would like to point that it is difficult to explain a complex project within a limited number of pages. A thorough review of all the methods is therefore difficult. The critical points raised below are mainly minor or a result of limited space to provide a sufficient level of detail.

Having said that, I think there are some aspects which could be improved but nothing which cannot be sorted out by small adjustments.

- The authors mention that they will conduct a prospective case control study. However, I don't see anything prospective in this approach. First the disease status is determined and than risk factors are assessed retrospectively with a questionnaire. A long recruitment period does not make a study prospective. Prospective case control study designs are rather nested case control studies etc.

- Selection of controls: I am not sure if frequency matching does make a lot of sense. Given that in each recruitment center only very few persons per month will be recruited frequency matching by age and region will be difficult. I think pair matching would be more easy to implement. It is not clear to me in which way community controls will be selected. In the cited reference controls are selected by random landline dialling and selecting one person randomly via the birthday method. How can this approach be combined with matching on age? I highly recommend to increase the ration of the occupationally matched controls - 30 cases and 30 controls can't tell you much, especially in the view that the risks differ among different occupations.

- "Establishing a cohort of patients for long term follow-up" is a strong statement for one follow-up interview after 6 months.

- How many human LS samples will be available for sequencing? I understand that the applicants don't know it at the moment but an idea how a good and a worse case scenario would look like would be beneficial. I fear it will be below 30.

- The sample size calculation for the less common exposures is hard to follow. Either the 15% exposure is referring to the controls - in this case an exposure of almost 30% in the cases is hard to refer to as less common. Or the 15% is referring to the cases which would translate to about 8% in the controls - in this case 450 participants are likely not be sufficient for 80% power. The provided reference [9] makes things even more confusing: in this paper they found an overall prevalence of pig owner (cases and controls) of about 5%.

- Risk factor analysis: How can be severity of symptoms or infecting serovars be explanatory variables to determine risk factors in a case control study? They occur, per definition, only in the cases. 'Random forest' is a classification method. How it can be contribute to the assessment of risk factors is not clear to me.

Health Significance Score: 6

It is always difficult to translate research findings directly into interventions. But I think the research might provide some important input to help to design future interventions.

Research Team Score: 6

No doubt, this is a strong research team. The combination of human and veterinarian epidemiologists/health professionals is quite powerful and to have a bio-statistician on board is certainly a plus. The advisory board is well selected.

General comments
