# Supplementary material for: Leptospirosis in Aotearoa New Zealand: Protocol for a Nationwide Case-Control Study
Source: JMIR Res Protoc. 2023 Jun 8;12:e47900. doi: 10.2196/47900 (PMC10288348; doi:10.2196/47900)
Supplement: Multimedia Appendix 5 [file resprot_v12i1e47900_app5.docx]

**Multimedia Appendix 5: Suspected patient information sheet**

**Participant Sample and Survey Information Sheet**

| Study title: | **Emerging Sources and Pathways for Leptospirosis: a paradigm shift** | | |
| --- | --- | --- | --- |
| Locality: | **Massey University** | Ethics committee ref.: | **19/STH/80** |
| Lead investigator: | **Jackie Benschop** | Contact phone number: | **06 951 6994** |

What is the purpose of the study?

The Molecular Epidemiology and Public Health Laboratories (mEpiLab) at Massey University has been funded by the Health Research Council of New Zealand to understand how people get leptospirosis. Understanding this can help us prevent future spread of the disease by implementing prevention and intervention strategies.

This study has been approved by the Health and Disability Ethics Committee, reference number 19/STH/80.

This Information Sheet will help you decide if you would like to take part in this study. It sets out **what your participation would involve**, what the benefits and risks to you may be, and what will happen after the study ends. We will go through this information with you and answer any questions you may have. This document is 10 pages long, including the Consent Form. Please make sure you have read and understood all the pages.

**If you agree to take part in this study, please sign the Consent Form on the last page of this document and hand back pages 9 and 10 to your doctor/nurse**

Please keep the Information Sheet and the Consent Form from pages 1 to 8 for your reference.

What will my participation in the study involve?

Leptospirosis, commonly known as “lepto”, can cause severe and sometimes long lasting illness. Your GP suspects you may have leptospirosis and we would like to invite you to be a part of this study. Your decision to participate is your choice.

If you choose to participate:

1. We will request one urine sample and two blood samples to test for lepto in a research lab, in addition to any tests that your doctor orders to test for lepto in a diagnostic lab.
2. We will request a copy of your doctor-ordered laboratory test results. **If your test results are negative for lepto, this is the end of your participation**
3. If your tests results are positive for lepto, we will ask you to participate further in the study. This includes consenting to approximately a **30 minute telephone survey.**
4. Depending on your answers to the survey, you may be eligible to participate further in the study. We will send you details of these in the mail.

What is the purpose of the survey?

The survey will be used to compare the habits and experiences of a group of people who have recently been diagnosed with leptospirosis (cases), with the habits and experiences of a group of people who have not had this illness in the past 4 weeks (controls). We can then look at these results to determine what aspects of people’s habits and experiences put them more at risk of contracting leptospirosis. This information can be used to put measures in place to reduce the number of people getting leptospirosis.

what sort of questions will be asked?

You will be answering questions that will cover aspects of your health, contact with animals and water, places of travel and activities you may have taken part in the month before contracting leptospirosis as well as a brief assessment on your emotional wellbeing in that month. This telephone survey will be conducted by Massey University researchers and will take approximately 30 minutes depending on how many of the questions are relevant to you. All answers will be recorded and transcribed by the researchers into a database which only the research team will have access to. We will schedule appointments to call at a time that is convenient. Please let your household members know that Massey University will be calling to schedule an appointment with you.

how will data be de-identified?

All identifying information will be removed from 1) samples and 2) survey data and be given unique codes (de-identified). All identifying information and the de-identified dataset will be stored in a password encrypted database, using a secure server housed at the Hopkirk Institute, Massey University, which is a restricted access facility. Participants will not be identifiable in any of the reports or presentations of findings from this survey. Researchers will ensure that storage of all information will be in accordance with the requirements of the Privacy Act 1993. This is also a requirement of the Health and Disabilities Ethics Committee approval.

How will the results be used?

The answers you give in the survey will be added to other people’s answers to create a de-identified dataset. This de-identified data will then be used for data analysis to help determine what the important risk factors are for leptospirosis. This information will be used to inform risk reduction strategies and information on pathways for infection such as flooding will inform health messaging.

What are the possible benefits and risks of this study?

There will be public health benefits for the New Zealand society as a result of this study. Your answers can help improve the understanding of risk factors for leptospirosis and will provide evidence for policies and practices to lower incidence and health consequences.

Some questions in the survey regarding your health and wellbeing such as those around anxiety and depression may cause you some discomfort or distress. During the phone interview, you may like to have a support person present or inform the interviewer whom you would like us to contact should you feel distressed. Otherwise, we have prepared a list of support services (see section “Who Do I Contact For More Information or If I have Concerns) that you can access. You can decline to answer any questions during the telephone interview without providing an explanation or can request to reschedule the interview to suit your circumstances.

Who pays for the study?

Massey University will pay for all tests. You will not have to pay any money to be part of this study but you will receive a $30 supermarket voucher as koha for submitting samples for research purposes in recognition for your participation in the study.

What if something goes wrong?

If you were injured in this study, which is unlikely, you would be eligible for compensation from ACC just as you would be if you were injured in an accident at work or at home. You will have to lodge a claim with ACC, which may take some time to assess. If your claim is accepted, you will receive funding to assist in your recovery.

If you have private health or life insurance, you may wish to check with your insurer that taking part in this study won’t affect your cover.

What are my rights?

- Participation in this study is voluntary and there will be no disadvantage to you if you choose to decline or withdraw from the study.
- You have the right to access information about yourself that is collected as part of this study. The researchers will give you a copy of this upon request.
- We will notify your GP of any significant results that becomes available during the study that may impact your health.
- Your privacy will be protected through the established protocols of Massey University. Your personal information will not be stored with any health results or information.
- This study will look at DNA from bacteria. We will not be looking at human DNA or genetics. Your samples will not be included in any research into human genomics.

What happens after the study or if I change my mind?

- Your samples will be kept for a minimum of 3 years (until May 30, 2022). After that time all samples will be destroyed by incineration. Any health information collected as part of this study will be held for a minimum of 10 years.
- If you change your mind during the study, you can withdraw from the study at any point before the first draft of the final report is written (September 2021) by contacting the study coordinator Dr. Shahista Nisa, phone: (06) 951 6918, email: [s.nisa@massey.ac.nz](mailto:s.nisa@massey.ac.nz). All data and samples relating to you will be permanently and securely destroyed upon withdrawal except for bacterial cultures which will be kept as an anonymized sample.

Maori Tissue Statement

You may hold beliefs about a sacred and shared value of all or any tissue samples removed. The cultural issues associated with storing your tissue should be discussed with your family/ whānau as appropriate. There are a range of views held by Māori around these issues; some iwi disagree with storage of samples citing whakapapa and advise their people to consult before participating in research where this occurs. However, it is acknowledged that individuals have the right to choose.

Who do I contact for more information or if I have concerns?

If you have any questions, concerns or complaints about the study at any stage, you can contact:

Name: Shahista Nisa

Phone: (06) 951 6918

Email: s.nisa@massey.ac.nz

If you want some additional information about leptospirosis in New Zealand or the study “Emerging sources and Pathways for Leptospirosis: a paradigm shift**”** please go to leptospirosis.org.nz

If you want to talk to someone who is not involved with the study, you can contact an independent health and disability advocate on:
 Phone: 0800 555 050
 Fax: 0800 2 SUPPORT (0800 2787 7678)
 Email: advocacy@hdc.org.nz

For Maori Health support, talk to your whānau in the first instance. Alternatively, you can reach a Maori support person in your area at:

**Hawke’s Bay DHB**

Name: John Barry Heperi-Smith

Telephone number: 0272056638

Email: JB.Heperi-Smith@hbdhb.govt.nz

You can also contact the health and disability ethics committee (HDEC) that approved this study on:

Phone: 0800 4 ETHICS Email: hdecs@moh.govt.nz

Below is a list of contact details for health professionals and counsellors if you wish to seek further medical advice. These professionals are not involved with the study.

- 1. Free call or text **1737** any time for support from a trained counsellor
  2. Health line – 0800 611 116 any time for support from a registered nurse
  3. Lifeline – 0800 543 354
  4. The Depression helpline – 0800 111 757
  5. Rural Support Trust -0800787254

**Participant Sample and Survey**

**Consent Form**

**Please tick to indicate you consent to the following on pages 8-9. Please note that if you answer “NO” to anything in this section, you WILL NOT be able to participate in this study**

| I am 16-years or older | Yes 🞏 | No 🞏 |
| --- | --- | --- |
| I have read, or have had read to me, and I understand this Participant Information Sheet. | Yes 🞏 | No 🞏 |
| I have been given sufficient time to consider whether or not to participate in this study. | Yes 🞏 | No 🞏 |
| I am satisfied with the answers I have been given regarding the study and I have a copy of this consent form and information sheet | Yes 🞏 | No 🞏 |
| I understand that taking part in this study is voluntary (my choice) and that I may withdraw from the study at any time until September 2021 without this affecting my medical care. | Yes 🞏 | No 🞏 |
| I consent to the research staff collecting and processing my information, including information about my health. | Yes 🞏 | No 🞏 |
| I consent to my GP or current provider being informed about my participation in the study and of any significant results obtained during the study. | Yes 🞏 | No 🞏 |
| I agree to an approved auditor appointed by the New Zealand Health and Disability Ethic Committees, or any relevant regulatory authority or their approved representative reviewing my relevant medical records for the sole purpose of checking the accuracy of the information recorded for the study. | Yes 🞏 | No 🞏 |
| I understand that my participation in this study is confidential and that no material which could identify me personally, will be used in any reports on this study. | Yes 🞏 | No 🞏 |
| I understand the compensation provisions in case of injury during the study. | Yes 🞏 | No 🞏 |
| I know whom to contact if I have any questions about the study in general. | Yes 🞏 | No 🞏 |
| I understand my responsibilities as a study participant. | Yes 🞏 | No 🞏 |
| I understand that samples collected as part of this study will be held for a minimum of 3 years. | Yes 🞏 | No 🞏 |
| I understand that any health information collected as part of this study will be held for a minimum of 10 years. | Yes 🞏 | No 🞏 |
| I consent to the research staff contacting me. | Yes 🞏 | No 🞏 |
| I consent to my blood and urine being used for research purposes. | Yes 🞏 | No 🞏 |
| I consent to participate in a telephone questionnaire to be given in English if it is confirmed that I have lepto. | Yes 🞏 | No 🞏 |
| I consent for my answers to be used for research purposes only. | Yes 🞏 | No 🞏 |
| **Please tick to indicate your consent to the following OPTIONAL consents (please note that if you answer “NO” to anything below, you WILL still be able to participate in this study)** | | |
| If I decide to withdraw from the study, I agree that bacterial cultures isolated from my samples may continue to be processed | Yes 🞏 | No 🞏 |
| I consent to the research staff receiving a copy of my lab results | Yes 🞏 | No 🞏 |
| I wish to receive a summary of the results from the study. | Yes 🞏 | No 🞏 |
| I wish to receive a $30 voucher | Yes 🞏 | No 🞏 |

*This page is intentionally left blank*

**Consent Form**

Initial:

Date:

**Participant Sample and Survey**

**Please tick to indicate you consent to the following on pages 8-9. Please note that if you answer “NO” to anything in this section, you WILL NOT be able to participate in this study**

| I am 16-years or older | Yes 🞏 | No 🞏 |
| --- | --- | --- |
| I have read, or have had read to me, and I understand this Participant Information Sheet. | Yes 🞏 | No 🞏 |
| I have been given sufficient time to consider whether or not to participate in this study. | Yes 🞏 | No 🞏 |
| I am satisfied with the answers I have been given regarding the study and I have a copy of this consent form and information sheet | Yes 🞏 | No 🞏 |
| I understand that taking part in this study is voluntary (my choice) and that I may withdraw from the study at any time until September 2021 without this affecting my medical care. | Yes 🞏 | No 🞏 |
| I consent to the research staff collecting and processing my information, including information about my health. | Yes 🞏 | No 🞏 |
| I consent to my GP or current provider being informed about my participation in the study and of any significant results obtained during the study. | Yes 🞏 | No 🞏 |
| I agree to an approved auditor appointed by the New Zealand Health and Disability Ethic Committees, or any relevant regulatory authority or their approved representative reviewing my relevant medical records for the sole purpose of checking the accuracy of the information recorded for the study. | Yes 🞏 | No 🞏 |
| I understand that my participation in this study is confidential and that no material which could identify me personally, will be used in any reports on this study. | Yes 🞏 | No 🞏 |
| I understand the compensation provisions in case of injury during the study. | Yes 🞏 | No 🞏 |
| I know whom to contact if I have any questions about the study in general. | Yes 🞏 | No 🞏 |
| I understand my responsibilities as a study participant | Yes 🞏 | No 🞏 |
| I understand that samples collected as part of this study will be held for a minimum of 3 years | Yes 🞏 | No 🞏 |
| I understand that any health information collected as part of this study will be held for a minimum of 10 years | Yes 🞏 | No 🞏 |
| I consent to the research staff contacting me | Yes 🞏 | No 🞏 |
| I consent to my blood and urine being used for research purposes | Yes 🞏 | No 🞏 |
| I consent to participate in a telephone questionnaire to be given in English if it is confirmed that I have lepto | Yes 🞏 | No 🞏 |
| I consent for my answers to be used for research purposes only | Yes 🞏 | No 🞏 |
| **Please tick to indicate your consent to the following OPTIONAL consents (please note that if you answer “NO” to anything in this section, you WILL still be able to participate in this study)** | | |
| If I decide to withdraw from the study, I agree that bacterial cultures isolated from my samples may continue to be processed | Yes 🞏 | No 🞏 |
| I consent to the research staff receiving a copy of my lab results | Yes 🞏 | No 🞏 |
| I wish to receive a summary of the results from the study. | Yes 🞏 | No 🞏 |
| I wish to receive a $30 voucher | Yes 🞏 | No 🞏 |

Initial:

Date:

**Additional important information, please fill out all which apply to you:**

Name: ____________________________________________________________

Postal address: ____________________________________________________________

_________________________________________________________________________

Phone number: (____) _________________ Mobile number: ________________________

Email address: _____________________________________________________________

Best time to contact: 🞏 Anytime 🞏 9am-5pm 🞏 5pm-9pm

🞏 Other (specify) ___________________________________

**Declaration by participant:**

I hereby consent to take part in this study.

| Participant’s name: | |
| --- | --- |
| Signature: | Date: |
