# Supplementary material for: Leptospirosis in Aotearoa New Zealand: Protocol for a Nationwide Case-Control Study
Source: JMIR Res Protoc. 2023 Jun 8;12:e47900. doi: 10.2196/47900 (PMC10288348; doi:10.2196/47900)
Supplement: Multimedia Appendix 6 [file resprot_v12i1e47900_app6.docx]

**Multimedia Appendix 6: Case information sheet**

Shahista Nisa

Molecular Epidemiology and Public Health Laboratory

Massey University

Palmerston North

Phone: (06) 951 6918

Email: s.nisa@massey.ac.nz

[DATE]

[ADDRESS

ADDRESS

ADDRESS

ADDRESS]

**RE: Emerging Sources and Pathways for Leptospirosis: a paradigm shift**

Dear [PARTICIPANT NAME]

We are writing to you because your Public Health Officer [PHO NAME] from [DHB NAME] District Health Board has informed us that you would like to find out about the leptospirosis study run by Massey University. Thank you for allowing us to contact you.

Enclosed in this letter is a Participation Information Sheet and Consent Form for this study. This outlines the descriptions of what your participation may involve, how the data in this study will be collected and how the results will be used. Your contributions to the study will help others as a result of the medical and scientific knowledge gained.

You do not have to do anything at this point.

We will contact you in about a week to go through this information with you and answer any questions you may have about the study. You do not need to return the consent form to us, you can give your consent verbally to the interviewer when they call you.

If you would like some additional information about leptospirosis in New Zealand and the study “Emerging Sources and Pathways for Leptospirosis: a paradigm shift”, you can find this at leptospirosis.org.nz for your reference.

Yours sincerely,
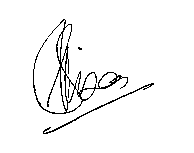


Shahista Nisa

Study coordinator

mEpiLab

**Participation Information Sheet**

| Study title: | **Emerging Sources and Pathways for Leptospirosis: a paradigm shift** | | |
| --- | --- | --- | --- |
| Locality: | **Massey University** | Ethics committee ref.: | **19/STH/80** |
| Lead investigator: | **Jackie Benschop** | Contact phone number: | **06 951 6994** |

What is the purpose of the study?

The Molecular Epidemiology and Public Health Laboratories (mEpiLab) at Massey University has been funded by the Health Research Council of New Zealand to understand how people get leptospirosis. Understanding this can help us prevent future spread of the disease by implementing prevention and intervention strategies.

This study has been approved by the Health and Disability Ethics Committee, reference number 19/STH/80.

This Information Sheet will help you decide if you would like to participate in the study. It sets out what your participation would involve, what the benefits and risks to you might be, and what will happen after the study ends. This document is 7 pages long, including the Consent Form. Please keep this Information Sheet and Consent Form for your reference.

What will my participation in the study involve?

Leptospirosis, commonly known as “lepto”, can cause severe and sometimes long lasting illness. You have been identified as someone who has recently contracted leptospirosis and has expressed interest in this study.

1. **Main study:** If you choose to participate in the study, we will ask you to consent to a 30-minute telephone survey. You have already done this. Thank you!

You will also have the option of participating further in the study if you wish.

1. You will be invited to participate in a follow-up telephone survey 6 months later to see you are doing.
2. We will seek your consent to access your doctor’s notes relating to this episode of leptospirosis.
3. We will seek your consent to access your laboratory test results.
4. We will seek your consent to access your diagnostic samples to run additional research tests.
5. If the main study survey reveals that you are from a farming or meat working background, we may ask you to participate in a 1-hour face-to-face semi-structured interview.
6. We will seek your consent to send you an invitation to join a long-term study. We will send details of this study to you closer to time (approximately in 2021) and you will have the opportunity to decline to participate in this if you wish when you receive this.

What is the purpose of these options

1. **Main study**: The survey will be used to compare the habits and experiences of a group of people who have recently been diagnosed with leptospirosis (cases), with the habits and experiences of a group of people who have not had this illness in the past 4 weeks. We can then look at these results to determine what aspects of people’s habits and experiences put them more at risk of contracting leptospirosis. This information can be used to put measures in place to reduce the number of people getting leptospirosis.
2. 6 month follow-up: Leptospirosis can sometimes cause long-term illness. We would like to assess how you are doing including the costs you incurred and any compensations that helped you in this 6 months.
3. Doctor’s notes: This information will tell us what your symptoms of leptospirosis was. We will use this data and compare it to other people’ symptoms of the illness. This may give us an indication of the kinds of symptoms that can lead to long-lasting illness versus a short-term illness.
4. Testing your diagnostics samples: Our research lab uses a number of different tests to study *Leptospira* (the bacteria that causes leptospirosis). This may further inform us on the type of bacteria that caused your illness.
5. Semi-structured interview: Farmers and meat workers are high-risk occupations. We would like to understand if vaccination and personal protection equipment can be improved to prevent future illness in these groups.
6. Long-term study: Some people with leptospirosis can be unwell for years. We would like to assess long-lasting leptospirosis symptoms and the support people have and/or need with a long-term follow-up study.

what sort of questions will be asked?

Main study: You will be answering questions that will cover aspects of your health, contact with animals and water, places of travel and activities you may have taken part in the month before contracting leptospirosis as well as a brief assessment on your emotional wellbeing in that month. Depending on your answers, this should take approximately 30 minutes. All answers will be recorded and transcribed by the researchers into a database.

6 month follow-up: You will be answering questions that will cover aspects of your health including duration of symptoms, the costs associated with your illness and any workplace compensation that you received. This interview will repeat the brief assessment on your emotional wellbeing in that month. This should take approximately 15 minutes. All answers will be recorded and transcribed by the researchers into a database.

Semi-structured interview: You will be asked about your experience with leptospirosis and workplace compensation, and your attitudes towards animal vaccination and personal protective equipment. This will be face-to-face and should take approximately one hour. All answers will be recorded on a dictaphone and transcribed by the researchers into a database.

who will be doing the interviews

All surveys and interviews will be conducted by Massey University researchers. We will call and schedule appointments at a time that is convenient for you. Please let your household members know that Massey University will be calling to schedule an appointment with you.

how will data be de-identified?

All interviews will be transferred onto a secure server. All identifying information will be removed from survey data and recorded interviews and be given unique codes (de-identified). This de-identified dataset will be stored in a password encrypted database, using a secure server housed at the Hopkirk Institute, Massey University, which is a restricted access facility. Participants will not be identifiable in any of the reports or presentations of findings from this survey. Researchers will ensure that storage of all information will be in accordance with the requirements of the Privacy Act 1993. This is also a requirement of the Health and Disabilities Ethics Committee approval.

How will the results be used?

Main study: The answers you give in the surveys will be added to other people’s answers to create a de-identified dataset. This de-identified data will then be used for data analysis to help determine what the important risks are for leptospirosis. This information will be used to inform risk reduction strategies and information on pathways for infection, such as flooding, will inform health messaging.

Burden of leptospirosis: All patients will be asked about the social, financial and emotional burden leptospirosis had on them so useful measures can be put in place to support future patients with leptospirosis.

Worker compensation for occupational leptospirosis: We hope to identify reasons behind accepted and rejected claims with the aim of providing clear guidelines to make access to compensation easy and straightforward for eligible patients.

Attitude assessment to current control strategies: This will include a subset of people with leptospirosis that are from the farming and meat working background. We aim to establish findings that will inform the improvement on current control strategies to prevent future illness in these groups.

What are the possible benefits and risks of this study?

There will be public health benefits for the New Zealand society as a result of this study. Your answers can help improve the understanding of risks for leptospirosis and will provide evidence for policies and practices to reduce the number of people who get leptospirosis.

Some questions in the survey regarding your health and wellbeing such as those around anxiety and depression may cause you some discomfort or distress. During the phone interview, you may like to have a support person present or inform the interviewer whom you would like us to contact should you feel distressed. Otherwise, we have prepared a list of support services (see section “Who Do I Contact For More Information or If I have Concerns”) that you can access. You can decline to answer any questions during the telephone interview without providing an explanation or can request to reschedule the interview to suit your circumstances.

Who pays for the study?

Massey University will pay for all tests.

You will not have to pay any money to be part of this study but can enter a draw to win a $500 supermarket voucher.

If you decide to participate in the semi-structured interview, you will receive a $30 supermarket voucher.

What if something goes wrong?

If you were injured in this study, which is unlikely, you would be eligible for compensation from ACC just as you would be if you were injured in an accident at work or at home. You will have to lodge a claim with ACC, which may take some time to assess. If your claim is accepted, you will receive funding to assist in your recovery.

If you have private health or life insurance, you may wish to check with your insurer that taking part in this study won’t affect your cover.

What are my rights?

- Participation in any part of this study is voluntary and there will be no disadvantage to you if you choose to decline or withdraw from the study.
- You have the right to access information about yourself that is collected as part of this study. The researchers will give you a copy of this upon request.
- We will notify your GP of any significant results that becomes available during the study that may impact your health.
- You privacy will be protected through the established protocols of Massey University. Your personal information will not be stored with any health results or information.
- This study will look at DNA from bacteria. We will not be looking at human DNA or genetics. Your samples will not be included in any research into human genomics.

What happens after the study or if I change my mind?

- Your samples will be kept for a minimum of 3 years (until July, 2022). After that time all samples will be destroyed by incineration. Any health information collected as part of this study will be held for a minimum of 10 years.
- If you change your mind during the study, you can withdraw from the study at any point before the first draft of the final report is written (September 2021) by contacting the study coordinator, Dr. Shahista Nisa, phone: (06) 951 6918, email: [s.nisa@massey.ac.nz](mailto:s.nisa@massey.ac.nz). After this date, results will no longer be connected to any information about you. All data and samples relating to you will be permanently and securely destroyed upon withdrawal.

Maori Tissue Statement

You may hold beliefs about a sacred and shared value of all or any tissue samples removed. The cultural issues associated with storing your tissue should be discussed with your family/ whānau as appropriate. There are a range of views held by Māori around these issues; some iwi disagree with storage of samples citing whakapapa and advise their people to consult before participating in research where this occurs. However, it is acknowledged that individuals have the right to choose.

Who do I contact for more information or if I have concerns?

If you have any questions, concerns or complaints about the study at any stage, you can contact the study coordinator:

Name: Shahista Nisa

Phone: (06) 951 6918

Email: s.nisa@massey.ac.nz

If you want to talk to someone who is not involved with the study, you can contact an independent health and disability advocate on:
 Phone: 0800 555 050
 Fax: 0800 2 SUPPORT (0800 2787 7678)
 Email: advocacy@hdc.org.nz

For Maori Health support, talk to your whānau in the first instance. Alternatively, you can reach a Maori support person in your area at:

**Hawke’s Bay DHB**

Name: John Barry Heperi-Smith

Telephone number: 0272056638

Email: JB.Heperi-Smith@hbdhb.govt.nz

You can also contact the health and disability ethics committee (HDEC) that approved this study on:

Phone: 0800 4 ETHICS Email: [hdecs@moh.govt.nz](mailto:hdecs@moh.govt.nz)

Below is a list of contact for health professionals and counsellors if you wish to seek further advice. These professionals are not involved with the study

- 1. Free call or text **1737** any time for support from a trained counsellor
  2. Health line – 0800 611 116 any time for support from a registered nurse
  3. Lifeline – 0800 543 354
  4. The Depression helpline – 0800 111 757

**Participation Consent Form**

**Please note that if you answer “NO” to anything in this section, you WILL NOT be able to participate in this study**

| I am 16-years or older | Yes 🞏 | No 🞏 |
| --- | --- | --- |
| I have read, or have had read to me, and I understand this Participant Information Sheet. | Yes 🞏 | No 🞏 |
| I have been given sufficient time to consider whether or not to participate in this study. | Yes 🞏 | No 🞏 |
| I am satisfied with the answers I have been given regarding the study and I have a copy of this consent form and information sheet | Yes 🞏 | No 🞏 |
| I understand that taking part in this study is voluntary (my choice) and that I may withdraw from the study at any time until September 2021 without this affecting my medical care. | Yes 🞏 | No 🞏 |
| I consent to the research staff collecting and processing my information, including information about my health. | Yes 🞏 | No 🞏 |
| I consent to my GP or current provider being informed about my participation in the study and of any significant results obtained during the study. | Yes 🞏 | No 🞏 |
| I agree to an approved auditor appointed by the New Zealand Health and Disability Ethic Committees, or any relevant regulatory authority or their approved representative reviewing my relevant medical records for the sole purpose of checking the accuracy of the information recorded for the study. | Yes 🞏 | No 🞏 |
| I understand that my participation in this study is confidential and that no material which could identify me personally, will be used in any reports on this study. | Yes 🞏 | No 🞏 |
| I understand the compensation provisions in case of injury during the study. | Yes 🞏 | No 🞏 |
| I know whom to contact if I have any questions about the study in general. | Yes 🞏 | No 🞏 |
| I understand my responsibilities as a study participant | Yes 🞏 | No 🞏 |
| I understand that any samples collected as part of this study will be held for a minimum of 3 years | Yes 🞏 | No 🞏 |
| I understand that any health information collected as part of this study will be held for a minimum of 10 years | Yes 🞏 | No 🞏 |
| I consent to the research staff contacting me | Yes 🞏 | No 🞏 |
| I consent to participate in a 30 minute telephone questionnaire to be given in English | Yes 🞏 | No 🞏 |
| I consent for my answers to be used for research purposes only | Yes 🞏 | No 🞏 |
| **Please indicate your consent to the following OPTIONAL consents (please note that if you answer “NO” to anything below, you WILL still be able to participate in this study)** | | |
| I wish to enter the draw to win a $500 voucher | Yes 🞏 | No 🞏 |
| I consent to participate in a 6 month follow-up telephone questionnaire to be given in English | Yes 🞏 | No 🞏 |
| If I am from a farming or meat working background, I wish to participate in a semi-structured face-to-face interview | Yes 🞏 | No 🞏 |
| I wish to receive a $30 koha for participating in the semi-structured face-to-face interview | Yes 🞏 | No 🞏 |
| I consent to the research staff receiving a copy of my lab results | Yes 🞏 | No 🞏 |
| I consent to have my diagnostic samples tested with research tests | Yes 🞏 | No 🞏 |
| I give consent to the research staff to assess my doctor’s notes by the researchers regarding my leptospirosis | Yes 🞏 | No 🞏 |
| I consent to be sent an invitation with details of a long-term follow-up study | Yes 🞏 | No 🞏 |
| I wish to receive information about the study | Yes 🞏 | No 🞏 |
| I wish to receive a summary of the results from the study. | Yes 🞏 | No 🞏 |

**Declaration by participant:**

I hereby have given verbal consent to take part in this study.

Participant name: _________________________________ Date: _________________

**Declaration by member of research team:**

I have given a verbal explanation of the research project to the participant, and have answered the participant’s questions.

I believe that the participant understands the study and has given informed consent to participate.

Researcher’s name: _________________________________ Date: _________________

Signature: __________________________
