# Supplementary material for: Leptospirosis in Aotearoa New Zealand: Protocol for a Nationwide Case-Control Study
Source: JMIR Res Protoc. 2023 Jun 8;12:e47900. doi: 10.2196/47900 (PMC10288348; doi:10.2196/47900)
Supplement: Multimedia Appendix 7 [file resprot_v12i1e47900_app7.docx]

**Multimedia Appendix 7: Control information sheet**

Study title: **Emerging Sources and Pathways for Leptospirosis**

Locality: **Massey University** Ethics committee ref.: **19/STH/80**

Lead investigator: **Jackie Benschop** Phone number: **(06) 951 6994**

What is the purpose of the study?

Massey University is doing a study to understand how people get leptospirosis. Understanding this can help us prevent future cases by implementing prevention and intervention strategies. This research is being carried out from July 2019 to September 2022.

This study will be undertaken by the Molecular Epidemiology and Public Health Laboratories (mEpiLab). mEpiLab has commissioned a market research company called UMR, a call-centre in Auckland, to carry out this telephone surveys.

This Information Sheet will help you decide if you would like to participate in the study. It sets out what your participation would involve, what the benefits and risks to you might be, and what would happen after the study ends.

What will my participation in the study involve?

You will receive a call from UMR sometime during the study period. They will go through this information sheet with you and answer any questions you may have.

If you choose to participate in the study, you will be asked to answer a 30-minute telephone survey.

Consent to participate in the survey and the survey can take place in the same call, however, we can also make an appointment for a more convenient time.

What is the purpose of the survey

The survey will be used to compare the habits and experiences of a group of people who have recently been diagnosed with leptospirosis (cases), with the habits and experiences of a group of people who have not had this illness in the past 4 weeks (that is yourself, this is known as the control). We can then look at these results to determine what aspects of people’s habits and experiences put them more at risk of contracting leptospirosis. This information can be used to put measures in place to reduce the number of people getting leptospirosis.

what sort of questions will be asked?

You will be answering questions that will cover aspects of your health such as any medical conditions you may have, contact with animals and water, places of travel and activities you may have taken part in the month before the survey call.

how will data be de-identified?

All identifying information will be removed from survey data and be given unique codes (de-identified). All identifying information will be contained in a separate and secure password encrypted database. The purpose of recording these details is to know who has been contacted. Researchers will ensure that storage of your information will be in accordance with the requirements of the Privacy Act 1993 which is to promote and protect individual privacy. This is also a requirement of the Health and Disabilities Ethics Committee approval.

How will the results be used?

The answers you give in the survey will be added to other people’s answers to create a de-identified dataset. This de-identified data will then be used for data analysis to help determine what the important risks are for leptospirosis and to calculate the burden of the disease to New Zealanders. Information on the sources and pathways for leptospirosis will inform ways to reduce future risk.

who will be doing the interviews

Massey University has commissioned UMR Market Research company, which has a call-centre in Auckland, to conduct the interviews.

What are the possible benefits of this study?

There will be public health benefits for the New Zealand society as a result of this study. Your answers can help improve the understanding of risk factors for leptospirosis and will provide evidence for policies and practices to reduce the number of people who get leptospirosis.

Who pays for the study?

The Health Research Council of New Zealand is paying Massey University to carry out this study. You will not have to pay any money to be a part of this study.

What if something goes wrong?

This study involves answering survey questions over the phone. The risks of this study to you is low.

In the highly unlikely event of injury from participating this this study, you would be eligible for compensation from ACC just as you would be if you were injured in an accident at work or at home. You will have to lodge a claim with ACC, which may take some time to assess. If your claim is accepted, you will receive funding to assist in your recovery.

If you have private health or life insurance, you may wish to check with your insurer that taking part in this study won’t affect your cover.

What are my rights?

- Participation in this study is voluntary. There will be no disadvantage to you if you choose to decline or withdraw from the study.
- You have the right to access information about yourself that is collected as part of this study. The researchers will give you a copy of this upon request.
- You privacy will be protected through the established protocols of Massey University and of UMR Market Research. Your personal information will not be stored with any health results or information.

What happens after the study or if I change my mind?

- Any health information collected as part of this study will be held for a minimum of 10 years.
- If you change your mind during the study, you can withdraw from the study at any point before the first draft of the final report is written (September 2021) by contacting the study coordinator, Dr. Shahista Nisa, phone: (06) 951 6918, email: [s.nisa@massey.ac.nz](mailto:s.nisa@massey.ac.nz). After this date, results will no longer be connected to any information about you. All data relating to you will be permanently and securely destroyed.

Who do I contact for more information or if I have concerns?

1. If you have any questions, concerns or complaints about the study at any stage, you can contact the study coordinator:

Name: Shahista Nisa

Phone: (06) 951 6918

Email: s.nisa@massey.ac.nz

1. If you want to talk to someone who is not involved with the study, you can contact an independent health and disability advocate on:

Phone: 0800 555 050

Fax: 0800 2 SUPPORT (0800 2787 7678)

Email: advocacy@hdc.org.nz

1. For Maori Health support, talk to your whānau in the first instance. Alternatively, you can reach a Maori support person in your area at:

Northland (09) 430 4100

Waikato (07) 834 3644

Hawke’s Bay 027 205 6638

Taranaki (06) 753 7777 ext: 8826

Waitemata and Auckland (09) 486 8324 ext: 42324

MidCentral (06) 350 8210

Tairawhiti (06) 869 1311 ext: 8876

Capital and Coast (04) 806 0948

Hutt Valley (04) 5666 999

Wairarapa (06) 946 4431

Whanganui (06) 348 3377 ext: 8377
Bay of Plenty (07) 579 8564

Lakes 027 226 4370

Southern  (03)4740 999 ext: 58649

Nelson Marlborough 027 246 8905

Canterbury (03) 364 0640 ext: 86154

South Canterbury (03) 615 5180

West Coast (03) 769 7400 ext: 2802

1. You can also contact the health and disability ethics committee (HDEC) that approved this study on:

Phone: 0800 4 ETHICS

Email: [hdecs@moh.govt.nz](mailto:hdecs@moh.govt.nz)

**Consent Form - Controls**

**Please note that if you answer “NO” to anything in this section, you WILL NOT be able to participate in this study**

| I am 16-years or older | Yes 🞏 | No 🞏 |
| --- | --- | --- |
| I have read, or have had read to me, and I understand this Participant Information Sheet. | Yes 🞏 | No 🞏 |
| I have been given sufficient time to consider whether or not to participate in this study. | Yes 🞏 | No 🞏 |
| I am satisfied with the answers I have been given regarding the study and I have a copy of this consent form and information sheet | Yes 🞏 | No 🞏 |
| I understand that taking part in this study is voluntary (my choice) and that I may withdraw from the study at any time without this affecting my medical care. | Yes 🞏 | No 🞏 |
| I consent to the research staff collecting and processing my information, including information about my health. | Yes 🞏 | No 🞏 |
| I agree to an approved auditor appointed by the New Zealand Health and Disability Ethic Committees, or any relevant regulatory authority or their approved representative reviewing my relevant medical records for the sole purpose of checking the accuracy of the information recorded for the study. | Yes 🞏 | No 🞏 |
| I understand that my participation in this study is confidential and that no material which could identify me personally, will be used in any reports on this study. | Yes 🞏 | No 🞏 |
| I understand the compensation provisions in case of injury during the study. | Yes 🞏 | No 🞏 |
| I know whom to contact if I have any questions about the study in general. | Yes 🞏 | No 🞏 |
| I understand my responsibilities as a study participant | Yes 🞏 | No 🞏 |
| I understand that any health information collected as part of this study will be held for a minimum of 10 years | Yes 🞏 | No 🞏 |
| I consent to participate in a 25 minute telephone questionnaire to be given in English | Yes 🞏 | No 🞏 |
| I consent for my answers to be used for research purposes only | Yes 🞏 | No 🞏 |
| **Please tick to indicate your consent to the following OPTIONAL consents (please note that if you answer “NO” to any of the below you WILL still be able to participate in this study)** | | |
| I wish to receive information about the study | Yes 🞏 | No 🞏 |
| I wish to receive a summary of the results from the study. | Yes 🞏 | No 🞏 |

**Declaration by participant:**

I hereby have given verbal consent to take part in this study.

Participant name: _________________________________ Date: _________________

**Declaration by member of UMR Market Research:**

I have given a verbal explanation of the research project to the participant and have answered the participant’s questions.

I believe that the participant understands the study and has given informed consent to participate.

Market Researcher’s name: ___________________________ Date: _________________

Signature: ___________________________________________________________
