# Supplementary material for: Leptospirosis in Aotearoa New Zealand: Protocol for a Nationwide Case-Control Study
Source: JMIR Res Protoc. 2023 Jun 8;12:e47900. doi: 10.2196/47900 (PMC10288348; doi:10.2196/47900)
Supplement: Multimedia Appendix 8 [file resprot_v12i1e47900_app8.docx]

**Multimedia Appendix 8: Case questionnaire**

[COMMENTS WRITTEN IN SQUARE BRACKETS REPRESENTS NOTES FOR THE INTERVIEWER]

Participants Name: ______________________________ Participant ID: _____

Interviewer name: __________________________________

Number of attempts to contact: _______________________ Date of first contact: ___________

Interview date: ____________

Support person name: _______________________________

Support contact number: _____________________________

Hello, my name is Shahista Nisa and I am calling you from Massey University.

1. Are you [PARTICIPANTS NAME]?

Yes [GO TO INTRODUCTION]

No

1. May I speak to [PARTICIPANTS NAME] please?

Yes [GO TO INTRODUCTION]

No

1. When would be a good time to reach [PARTICIPANT NAME]?

___________________________ [ENTER TIME]

1. What is the best number to reach [PARTICIPANT NAME]?

___________________________ [ENTER NUMBER]

**INTRODUCTION FOR SUSPECTED CASES (provided previous consent)**

Hello [PARTICIPANT NAME]. I am calling you because you have recently been diagnosed with leptospirosis and you consented to take part in our survey. Thank you for agreeing to participate in this research. [PAUSE]

1. Is this a good time to talk?

Yes [GO TO Q4]

No

1. When would be a good time to reach you?

________________________ [ENTER TIME]

1. What is the best number to reach you?

________________________ [ENTER NUMBER]

**ELIGIBILITY**

1. Before we begin the survey, I need to confirm, are you 16 years or older?

Yes [GO TO Consent]

No. Sorry we are not able to include you in the study. Thank you for your time. Do you have any questions for me [PARTICIPANT NAME] before we conclude?

**CONSENT FOR TIER 1**

In order to comply with the rules specified by the Health and Disability Ethics Committee, we will go through a verbal consent process.

1. Did you fill in a Participant Sample and Survey Consent form at the doctor’s?

Yes [ GO TO TIER 1 CONFIRMED PISCF]

No [ GO TO TIER 2/3 PISCF]

**INTRODUCTION FOR NEW CASES**

Hello [PARTICIPANTS NAME]. I am calling you because you have recently been diagnosed with leptospirosis. I was notified by your health protection officer from [DHB NAME] that you wish to learn more about this study. Thank you for considering this.

1. Is this a good time to talk?

Yes [ GO TO Q4]

No

1. When would be a good time to reach you?

________________________ [ENTER TIME]

1. What is the best number to reach you?

________________________ [ENTER NUMBER]

**ELIGIBILITY**

1. Before we begin, I need to confirm, are you 16 years or older?

Yes [ GO TO Consent]

No. Sorry we are not able to include you in the study. Thank you for your time. Do you have any questions for me [PARTICIPANTS NAME] before we conclude?

**CONSENT FOR NEW CASES**

In order to comply with the rules specified by the Health and Disability Ethics Committee and for your information, I will go through some information about this study and get your verbal consent if you wish to participate in this study.

1. Did you receive a Participant Information Sheet and Consent Form from Massey University?

Yes [ GO THROUGH “PISCF3 READING” SHEET AND VERBAL CONSENT, SKIP Q6 and Q7]

No

1. We can send another one out to you. Do you prefer email or post?

Email

Post

[CONFIRM DETAILS]

1. In the meantime, can I go ahead with the verbal consent process?

Yes [ GO THROUGH “PISCF3 READING” SHEET WITH THEM AND VERBAL CONSENT]

No. Thank you for your time. We will call you back in about a week’s time. Hopefully you would have received the Participant Information Sheet and Consent form by then.

Most of the questions I’ll be asking will either have a “Yes “or “No” answer or I will be giving you options to select answers from. I will ask you some questions that you may consider sensitive. Please remember, it is your choice to answer these questions and we can stop at any point. Do you have any questions for me before we begin the survey?

**SECTION 3: IDENTIFICATION**

I would like to confirm your contact details please

1. What is your address? [ENTER TEXT AND NUMBER]

NUMBER: _____________ STREET NAME: ____________________________

SUBURB: ______________ TOWN: _________________________ POST CODE: _________

1. What is the name of your nearest primary school?

[ENTER TEXT]

1. Do you live? [READ OPTIONS AND SELECT ONE]

In a town or city

On a lifestyle block *i.e.* <10 acres

On a farm

1. What is the best contact number for you?

[ENTER NUMBER]

1. What is your email address?

[ENTER TEXT]

1. What is the best method to contact you? [READ OPTIONS AND SELECT ALL THAT APPLIES]

Telephone

Email

Others [SPECIFY] ­­­­­­­___________________________________

1. When is the best time to contact you? [READ OPTIONS AND SELECT ONE]

Anytime

Weekends [ASK FOR POSSIBLE TIME OPTIONS] _________________

Weekdays [ASK FOR POSSIBLE TIME OPTIONS] __________________

1. What is the name of your usual GP?

[ENTER TEXT]

1. What is the clinic or practice name your usual GP works in?

[ENTER TEXT]

1. Can your provide us with an email and/or phone number for your GP?

[ENTER TEXT]

1. Did your usual GP diagnose your leptospirosis?

Yes [GO TO SECTION 4]

No [GO TO QUESTION 12]

1. What is the name of the doctor that diagnosed your leptospirosis?

[ENTER TEXT]

1. What is the clinic or practice name this GP works in?

[ENTER TEXT]

1. Can you provide us with an email and/or phone number for the GP who diagnosed your lepto?

[ENTER TEXT]

**SECTION 4: CLINICAL COURSE AND OUTCOME**

Thank you [PARTICIPANTS NAME] for providing this information. I will now ask you some questions about when you got sick with leptospirosis.

1. Do you remember the date when you first became ill with leptospirosis?

[ENTER DATE: DD/MM/YYYY]_____________________________

1. Do you remember the date when you first sought medical help for this?

[ENTER DATE: DD/MM/YYYY]_____________________________

1. Were you hospitalized for leptospirosis?

Yes

No [GO TO QUESTION 6]

1. How many nights were you in hospital?

[ENTER NUMBER]

1. Were you admitted in ICU?

Yes [SPECIFY HOW MANY DAYS]

No

1. Did you take any time off work when you were sick with leptospirosis?

Yes

No [GO TO QUESTION 8]

1. How many days did you take off? [ENTER NUMBER]
2. When you first became ill with leptospirosis, which of the following symptoms did you experience?

[READ OPTIONS AND SELECT ALL THAT APPLY]

Headache

Fever

Sore eyes

Sensitivity to light

Muscle pain in your legs

Muscle pain in your back

Nausea

Diarrhoea

Abdominal pain

Fatigue

Cough

Others [SPECIFY] _______________

1. Do you still have any of these symptoms?

[SELECT ALL THAT APPLY]

Headache

Fever

Sore eyes

Sensitivity to light

Muscle pain (legs)

Muscle pain (back)

Nausea

Diarrhoea

Abdominal pain

Fatigue

Cough

Others [SPECIFY] _______________

None

1. Were you given any antibiotics for your leptospirosis?

Yes

No [GO TO QUESTION 14]

Unsure

1. What antibiotics were you given? [READ OPTIONS]

Doxycycline

Amoxicillin

Erythromycin

Unsure

Others [SPECIFY]

1. Did you miss any dose?

Yes

No [GO TO QUESTION 14]

Unsure

1. How many times did you miss a dose?

Once or twice

More than twice

Unsure

1. Do you know how you can get leptospirosis?

Yes

No [GO TO SECTION 5]

1. Can you give me one example of how you can get leptospirosis? [DO NOT READ]

Animal’s faeces

Animal’s urine

Water

Soil

Other [SPECIFY]

Refused to answer

The next set of questions are more about you.

**SECTION 5: DEMOGRAPHY**

1. What is your date of birth?

Date of Birth [FORMAT: DD/MM/YYYY] _____________________

Age [IF UNWILLING TO GIVE DATE OF BIRTH, REQUEST AGE] _________________

Age band [IF UNWILLING TO GIVE AGE, ASK FOR AGE BAND]

<20

20-29

30-39

40-49

50-59

60-69

70 +

Refuse to answer [DO NOT READ]

1. Which gender do you identify with?

Male

Female

Other

1. Which of the following ethnic groups do you belong to? [READ OPTIONS, SELECT ALL THAT APPLY]

NZ European Niuean

NZ Māori Chinese

Samoan Indian

Cook Island Māori Others [SPECIFY]

Tongan

1. What is your highest education level? [READ OPTIONS]

No education

Primary education

Secondary education

Tertiary education

1. What is your total personal income, including income support before tax, in the past 12 months? [READ OPTIONS]

Zero income

$1 to $14,000

$14,001 to $48,000

$48,001 to $70,000

over $70,001

Refuse to answer [DO NOT READ]

Now I am going to ask you some questions about what you did in the month before you first became ill.

1. What was your job or occupation in the month before you first became ill? If you had more than one job, please tell me all of them.

[THE STUDY IS PARTICULARLY INTERESTED IN OCCUPATIONS THAT INVOLVE ANIMALS OR OUTDOOR ENVIRONMENTS. OCCUPATIONS OF THIS SORT SHOULD BE PROBED FOR A MORE SPECIFIC DESCRIPTION. FOR EXAMPLE, IF THE JOB TITLE IS FARMER ASK IF THEY ARE DAIRY FARMER, BEEF FARMER, SHEEP FARMER, CROP FARMER, MIXED FARMING OR OTHER TYPES]

Job title 1: __________________

Job title 2: ___________________

Job title 3: ____________________

Not working/retired: ______________

Other (specify): ____________________

1. Did you do any other work *e.g.* part-time work, help a friend or work outside in the month before you first became ill?

Yes [SPECIFY JOBS]

No

Unsure

# SECTION 6: ANIMAL EXPOSURE

I will now ask you about your animal contacts in the month before you first became ill.

1. Did you come in direct contact with any of the following livestock or farmed animals or their urine in the month before you first became ill? [READ OPTIONS AND SELECT ALL THAT APPLY]
   1. If yes to any, approximately how many days in that month
   2. If yes to any, was it at work (W), outside of your paid work (O), both (B) or unsure (U)?

| **Livestock contact** | **Yes/No/Unsure** | **a. Days in month** | **b. W/O/B/U** |
| --- | --- | --- | --- |
| Dairy cattle | Yes/No/Unsure |  | W/O/B/U |
| Beef cattle | Yes/No/Unsure |  | W/O/B/U |
| Sheep | Yes/No/Unsure |  | W/O/B/U |
| Pigs | Yes/No/Unsure |  | W/O/B/U |
| Goats | Yes/No/Unsure |  | W/O/B/U |
| Deer | Yes/No/Unsure |  | W/O/B/U |
| Alpacas | Yes/No/Unsure |  | W/O/B/U |
| Horses | Yes/No/Unsure |  | W/O/B/U |
| Others [SPECIFY] |  |  | W/O/B/U |
| None [GO TO QUESTION 7] |  |  |  |

1. Were you involved in any of the following livestock associated activities in the month before you first became ill with leptospirosis? [READ OPTIONS AND SELECT ALL THAT APPLY]
   1. If yes to any, approximately how many days in that month
   2. If yes to any, was it at work (W), outside of your paid work (O) or both (B)?
   3. If yes to any, was the flock/herd vaccinated
      1. Fully vaccinated
      2. Partially vaccinated
      3. Not vaccinated at all
      4. Unsure [DO NOT READ]

| **Livestock** | **Livestock activities** | **Yes/No/Unsure** | **a. Days in month** | **b. W/O/B/U** | **c. Vaccinated against lepto** |
| --- | --- | --- | --- | --- | --- |
| [BEEF CATTLE] | Assisting calving | Yes/No/Unsure |  | W/O/B/U | 1 2 3 4 |
|  | Milking cows | Yes/No/Unsure |  | W/O/B/U | 1 2 3 4 |
|  | Docking/castrating calves | Yes/No/Unsure |  | W/O/B/U | 1 2 3 4 |
|  | Drenching cattle | Yes/No/Unsure |  | W/O/B/U | 1 2 3 4 |
| [DAIRY CATTLE] | Assisting calving | Yes/No/Unsure |  | W/O/B/U | 1 2 3 4 |
|  | Milking cows | Yes/No/Unsure |  | W/O/B/U | 1 2 3 4 |
|  | Docking/castrating calves | Yes/No/Unsure |  | W/O/B/U | 1 2 3 4 |
|  | Drenching cattle | Yes/No/Unsure |  | W/O/B/U | 1 2 3 4 |
| [SHEEP] | Assisting lambing | Yes/No/Unsure |  | W/O/B/U | 1 2 3 4 |
|  | Crutching or dagging sheep | Yes/No/Unsure |  | W/O/B/U | 1 2 3 4 |
|  | Shearing sheep | Yes/No/Unsure |  | W/O/B/U | 1 2 3 4 |
|  | Docking/castrating lambs | Yes/No/Unsure |  | W/O/B/U | 1 2 3 4 |
|  | Palpating udders of ewes | Yes/No/Unsure |  | W/O/B/U | 1 2 3 4 |
|  | Milking sheep | Yes/No/Unsure |  | W/O/B/U | 1 2 3 4 |
|  | Drenching sheep | Yes/No/Unsure |  | W/O/B/U | 1 2 3 4 |
| [SPECIFY ANIMAL SPECIES AND FILL IN A, B, C FOR EACH SPECIES] | Cleaning urine or faeces from yard surface?  Species:  Species:  Species:  Species: | Yes/No/Unsure |  | W/O/B/U | 1 2 3 4 |
|  | Home kill?  Species:  Species:  Species:  Species: | Yes/No/Unsure |  | W/O/B/U | 1 2 3 4 |
|  | Killing for animal welfare?  Species:  Species:  Species:  Species: | Yes/No/Unsure |  | W/O/B/U | 1 2 3 4 |
|  | Slaughtering animal?  Species:  Species:  Species:  Species: | Yes/No/Unsure |  | W/O/B/U | 1 2 3 4 |
|  | Dressing animal carcass?  Species:  Species:  Species:  Species: | Yes/No/Unsure |  | W/O/B/U | 1 2 3 4 |
| Other activities [SPECIFY ACTIVITY AND ANIMAL SPECIES] |  |  |  |  |  |

1. Have you had any contact with animals that have been newly introduced to the farm in the month before you first became ill with leptospirosis? [READ OPTIONS]

Yes

No [GO TO QUESTION 5]

Unsure [GO TO QUESTION 5]

1. Were the introduced animals fully vaccinated, partially vaccinated or not vaccinated at all?

Fully vaccinated

Partially vaccinated

Not vaccinated at all

Unsure [DO NOT READ]

1. Generally, did you use any of the following personal protective equipment while handling livestock bodies or carcasses in the month before you first became ill with leptospirosis? [READ OPTIONS AND SELECT ALL THAT APPLY]

Gloves

Boots

Apron

Goggles

Facemask

Others [SPECIFY]

Unsure [DO NOT READ]

None [DO NOT READ]

1. How often did you wash your hands after handling livestock in the month before you first became ill with leptospirosis? [READ OPTIONS]

All the time

Sometimes

Never

Others [SPECIFY]

1. Did you come in direct contact with any of the following wild animals in the month before you first became ill with leptospirosis? [READ OPTIONS AND SELECT ALL THAT APPLY]
   1. If yes to any, approximately how many days in that month
   2. If yes to any, was it at work (W), outside of your paid work (O), both (B) or unsure (U)?

| **Wild animal contact** | **Yes/No/Unsure** | **a. Days in month** | **b. W/O/B/U** |
| --- | --- | --- | --- |
| Possum | Yes/No/Unsure |  | W/O/B/U |
| Hedgehog | Yes/No/Unsure |  | W/O/B/U |
| Wild rats | Yes/No/Unsure |  | W/O/B/U |
| Wild mice | Yes/No/Unsure |  | W/O/B/U |
| Ferret/Stoat/Weasel | Yes/No/Unsure |  | W/O/B/U |
| Wild rabbits | Yes/No/Unsure |  | W/O/B/U |
| Wild deer | Yes/No/Unsure |  | W/O/B/U |
| Wild goat | Yes/No/Unsure |  | W/O/B/U |
| Feral cats | Yes/No/Unsure |  | W/O/B/U |
| Others[SPECIFY] | Yes/No/Unsure |  | W/O/B/U |
| None | Yes/No/Unsure |  | W/O/B/U |

1. Did you notice any evidence of wild animals, for example, droppings, gnawed feed bags or fruit damage in the month before you first became ill with leptospirosis? [READ OPTIONS AND SELECT ALL THAT APPLY]

| Wild animals | Possum  Hedgehog  Wild rats  Wild mice  Ferret/Stoat/Weasel  Wild rabbit | Wild deer  Wild goat  Wild pig  Feral cat  Others[SPECIFY]  None |
| --- | --- | --- |

1. Did you set any traps/poison for any wild animals in the month before you became ill? [READ OPTIONS AND SELECT ALL THAT APPLY]
2. If yes to any, approximately how many days in that month
3. If yes to any, was it at work (W), outside of your paid work (O), both (B) or unsure (U)?

| **Wild animal** | **Yes/No/Unsure** | **a. Days in month** | **b. W/O/B/U** |
| --- | --- | --- | --- |
| Possum | Yes/No/Unsure |  | W/O/B/U |
| Hedgehog | Yes/No/Unsure |  | W/O/B/U |
| Wild rats | Yes/No/Unsure |  | W/O/B/U |
| Wild mice | Yes/No/Unsure |  | W/O/B/U |
| Ferret/Stoat/Weasel | Yes/No/Unsure |  | W/O/B/U |
| Wild rabbits | Yes/No/Unsure |  | W/O/B/U |
| Wild deer | Yes/No/Unsure |  | W/O/B/U |
| Wild goat | Yes/No/Unsure |  | W/O/B/U |
| Feral cats | Yes/No/Unsure |  | W/O/B/U |
| Others[SPECIFY] | Yes/No/Unsure |  | W/O/B/U |
| None | Yes/No/Unsure |  | W/O/B/U |

1. Did you empty traps for any of the following animals in the month before you first became ill? [READ OPTIONS AND SELECT ALL THAT APPLY]
2. If yes to any, approximately how many days in that month
3. If yes to any, was it at work (W), outside of your paid work (O), both (B) or unsure (U)?

| **Wild animal** | **Yes/No/Unsure** | **a. Days in month** | **b. W/O/B/U** |
| --- | --- | --- | --- |
| Possum | Yes/No/Unsure |  | W/O/B/U |
| Hedgehog | Yes/No/Unsure |  | W/O/B/U |
| Wild rats | Yes/No/Unsure |  | W/O/B/U |
| Wild mice | Yes/No/Unsure |  | W/O/B/U |
| Ferret/Stoat/Weasel | Yes/No/Unsure |  | W/O/B/U |
| Wild rabbits | Yes/No/Unsure |  | W/O/B/U |
| Wild deer | Yes/No/Unsure |  | W/O/B/U |
| Wild goat | Yes/No/Unsure |  | W/O/B/U |
| Feral cats | Yes/No/Unsure |  | W/O/B/U |
| Others[SPECIFY] | Yes/No/Unsure |  | W/O/B/U |
| None | Yes/No/Unsure |  | W/O/B/U |

[IF NO TO QUESTION Q8, Q9 and Q10 GO TO QUESTION 13]

1. Did you use any of the following personal protective equipment while handling wildlife or traps in the month before you first became ill with leptospirosis? [READ OPTIONS AND SELECT ALL THAT APPLY]

Gloves

Boots

Apron

Goggles

Facemask

Others [SPECIFY]

Unsure [DO NOT READ]

None [DO NOT READ]

1. How often did you wash your hands after handling wildlife in the month before you first became ill with leptospirosis?

All the time

Sometimes

Never

Others [SPECIFY]

1. Did you come in direct contact with any of the following pet animals in the month before you first became ill with leptospirosis? [READ OPTIONS AND SELECT ALL THAT APPLY]
2. If yes to any, approximately how many days in that month
3. If yes to any, was it at work (W), outside of your paid work (O), both (B) or unsure (U)?

| **Pet animal contact** | **Yes/No/Unsure** | **a. Days in month** | **b. W/O/B/U** |
| --- | --- | --- | --- |
| Dogs (pets, farm or service) | Yes/No/Unsure |  | W/O/B/U |
| Cats | Yes/No/Unsure |  | W/O/B/U |
| Rats | Yes/No/Unsure |  | W/O/B/U |
| Mice | Yes/No/Unsure |  | W/O/B/U |
| Guinea pigs | Yes/No/Unsure |  | W/O/B/U |
| Rabbits | Yes/No/Unsure |  | W/O/B/U |
| Others[SPECIFY] | Yes/No/Unsure |  | W/O/B/U |

1. Did you clean up urine or faeces of any of the following pet animals in the month before you first became ill with leptospirosis? [READ OPTIONS AND SELECT ALL THAT APPLY]
2. If yes to any, approximately how many days in that month
3. If yes to any, was it at work (W), outside of your paid work (O), both (B) or unsure (U)?

| **Pet animal urine/faeces contact** | **Yes/No/Unsure** | **a. Days in month** | **b. W/O/B/U** |
| --- | --- | --- | --- |
| Dogs (pets, farm or service) | Yes/No/Unsure |  | W/O/B/U |
| Cats | Yes/No/Unsure |  | W/O/B/U |
| Rats | Yes/No/Unsure |  | W/O/B/U |
| Mice | Yes/No/Unsure |  | W/O/B/U |
| Guinea pigs | Yes/No/Unsure |  | W/O/B/U |
| Rabbits | Yes/No/Unsure |  | W/O/B/U |
| Others [SPECIFY] | Yes/No/Unsure |  | W/O/B/U |

Thinking back about all the animals that you mentioned you had contact with, including farmed, wild or pets:

1. Did you hunt any animals in the month before you became ill?

Yes [SPECIFY WHAT ANIMALS]

No

1. Were you involved with skinning or plucking animals in the month before you became ill?

Yes [SPECIFY WHAT ANIMALS]

No

1. Have you had contact with a dead animals that were not slaughtered on purpose at home work or elsewhere in the month before you became ill?

Yes [SPECIFY WHAT ANIMALS]

No

Unsure

1. Have you had contact with animals that aborted or have had still births?

Yes [SPECIFY ANIMALS]

No

Unsure

1. Did you handle any animal feed in the month before you first became ill with leptospirosis?

Yes [SPECIFY]

No

Unsure

1. If yes to any, approximately how many days in that month
2. If yes, was it at work (W), outside of your paid work (O), both (B), Unsure (U)
3. Did you generally use gloves when handling animal feed Yes (Y), No (N), Unsure (U)

| **Type of feed** | **a. Days in month** | **b. W/O/B/U** | **c. Gloves** |
| --- | --- | --- | --- |
|  |  | W/O/B/U | Y/N/U |
|  |  | W/O/B/U | Y/N/U |
|  |  | W/O/B/U | Y/N/U |
|  |  | W/O/B/U | Y/N/U |

Thank you [PARTICIPANTS NAME] for your answers, this is all I have about animal exposures. The next section will focus on water exposures.

**SECTION 7: WATER EXPOSURES**

1. What was the source of your water supply in your home in the month before you first became ill? [READ OPTIONS, SELECT ALL THAT APPLY and ASK IF THIS WATER WAS TREATED]

| **Home water supply** | **Treated (Yes/No/Unsure)** |
| --- | --- |
| Usual town supply | Yes/No/Unsure |
| Private bore/spring water | Yes/No/Unsure |
| Tanker truck water | Yes/No/Unsure |
| Roof/rain water | Yes/No/Unsure |
| Creek/stream water | Yes/No/Unsure |
| Other main source of drinking | Yes/No/Unsure |
| Unsure [DO NOT READ] | Yes/No/Unsure |

1. What was the source of water supply at your place of employment in the month before you first became ill? [READ OPTIONS, SELECT ALL THAT APPLY and ASK IF THIS WATER WAS TREATED]

| **Place of employment** | **Treated (Yes/No/Unsure)** |
| --- | --- |
| Usual town supply | Yes/No/Unsure |
| Private bore/spring water | Yes/No/Unsure |
| Tanker truck water | Yes/No/Unsure |
| Roof/rain water | Yes/No/Unsure |
| Creek/stream water | Yes/No/Unsure |
| Other main source of drinking | Yes/No/Unsure |
| Unsure [DO NOT READ] | Yes/No/Unsure |

1. Did you have contact with water from the following sources for recreational purpose in the month before you got ill? [READ OPTIONS, SELECT ALL THAT APPLY]

Ocean

River

Others [SPECIFY]

None [GO TO QUESTION 5]

1. Were you involved with any of the following recreational activities? [READ OPTIONS AND SELECT ALL THAT APPLY]
   1. If yes to any, select frequency

| **Water activity** | More than once a week | Once a week | Less than once a week |
| --- | --- | --- | --- |
| Swimming in river or fresh water |  |  |  |
| Boating |  |  |  |
| Fishing |  |  |  |
| Others [SPECIFY] |  |  |  |

1. Did you encounter any of the following situations in the month before you first became ill? [READ OPTIONS AND SELECT ALL THAT APPLY]
2. If yes to any, approximately how many days in that month
3. If yes to any, was it at work (W), outside of your paid work (O), both (B) or Unsure?

| **Situations** | **Yes/No/Unsure** | **a. Days in month** | **b. W/O/B/U** |
| --- | --- | --- | --- |
| Flooding | Yes/No/Unsure |  | W/O/B/U |
| Animal effluent | Yes/No/Unsure |  | W/O/B/U |
| Mud | Yes/No/Unsure |  | W/O/B/U |
| Drainage/plumbing work | Yes/No/Unsure |  | W/O/B/U |
| Landscaping | Yes/No/Unsure |  | W/O/B/U |
| Wetlands | Yes/No/Unsure |  | W/O/B/U |
| Other contact with water [SPECIFY] | Yes/No/Unsure |  | W/O/B/U |

Thank you [PARTICIPANTS NAME] for your answers, this is all I have about water exposures. The next section will focus on other exposures.

**SECTION 8: OTHER EXPOSURES**

1. Have you had any contact with soil, for example gardening or horticulture, in the month before you first became ill with leptospirosis?

Yes

No [GO TO QUESTION 6]

Unsure [GO TO QUESTION 6]

1. Was this part of your paid employment?

Yes

No

Unsure

1. Did you use animal manure fertilizer while gardening or during horticulture?

Yes

No

Unsure

1. Did you use any of the following personal protective equipment while gardening or during horticulture? [READ OPTIONS, SELECT ALL THAT APPLY]

Gloves

Boots

Goggles

Facemask

Others [SPECIFY]

Unsure [DO NOT READ]

None [DO NOT READ]

1. How often did you wash your hands after gardening in the month before you first became ill with leptospirosis? [READ OPTIONS]

All the time

Sometimes

Never

Others [SPECIFY]

1. Did you drink any raw milk in the month before you first became ill?

Yes

No

Unsure

1. Have you taken part in other outdoor activities in the month before you first became ill? [READ OPTIONS, SELECT ALL THAT APPLY]

Hiking/Walking

Camping

Others [SPECIFY]

No

1. Have you walked barefoot outside in the month before you first became ill?

Yes [SPECIFY HOW OFTEN]

No

Unsure

1. Did you travel outside New Zealand in the month before you first became ill?

Yes

No [GO TO Section 9]

Unsure [GO TO Section 9]

1. Can you please provide some details of that travel outside of New Zealand?

- Date of departure: [ENTER DATE DD/MM/YY]
- Date of return: [ENTER DATE DD/MM/YY]
- Countries of travel [ENTER TEXT]

**SECTION 9: WORKPLACE COMPENSATION**

In the next section, I will ask you some questions about workplace compensation for your leptospirosis.

1. Did you think you got leptospirosis as part of your paid employment?

Yes

No [GO TO SECTION 10]

Unsure

1. Were you eligible for Workers Compensation?

Yes

No [GO TO SECTION 10]

Unsure [GO TO SECTION 10]

1. Was a claim was lodged?

Yes [GO TO QUESTION 5]

No [GO TO QUESTION 4]

Unsure [GO TO SECTION 10]

1. Do you know why a claim was not lodged?

Yes [SPECIFY REASON] [GO TO SECTION 10]

No [GO TO SECTION 10]

1. Was the claim accepted?

Yes [GO TO QUESTION 7]

No [GO TO QUESTION 6]

Haven’t heard back yet [GO TO SECTION 10]

1. Did you receive any feedback about why your claim was not accepted?

Yes [SPECIFY REASON, GO TO QUESTION 8]

No [GO TO QUESTION 8]

1. Did you receive any of the following entitlement?

Income replacement

Treatment and rehabilitation costs

Lump-sum compensation

Other [SPECIFY]

Unsure

None

1. How would you rate your compensation experience? [READ OPTIONS AND SELECT ONE]

Extremely satisfied

Satisfied

Dissatisfied

Extremely dissatisfied

1. Could you identify up to 3 areas that would have improved your experience with workers compensation? [READ OPTIONS, SELECT ALL THAT APPLY]

Support from case managers

Communication

Timeliness

Others [SPECIFY]

**SECTION 10: HEALTH STATUS**

In the next section, I will be asking you about your general health.

1. Did you have a cut or scratch in the month before you first became ill?

Yes

No [GO TO QUESTION 3]

Unsure [GO TO QUESTION 3]

1. Did you cover your wound?

Yes

No

Unsure

1. Do you smoke cigarettes regularly, that is, one or more a day?

Yes [GO TO QUESTION 5]

No

Refused

1. Have you ever been a regular smoker of one or more cigarettes a day?

Yes

No

1. Do you suffer from any of the following medical conditions?
   1. If yes to any, can you tell me how many years you have had this condition for [READ OPTIONS AND SELECT ALL THAT APPLY]

| **Medical conditions** | **Yes/No/Unsure** | **Years** |
| --- | --- | --- |
| Hay fever | Yes/No/Unsure |  |
| Asthma | Yes/No/Unsure |  |
| Diabetes | Yes/No/Unsure |  |
| Heart disease | Yes/No/Unsure |  |
| Lung disease | Yes/No/Unsure |  |
| Anxiety | Yes/No/Unsure |  |
| Depression | Yes/No/Unsure |  |
| Other [SPECIFY] | Yes/No/Unsure |  |

1. Are you on any regular medication?

Yes [SPECIFY]

No

Unsure

Refused [DO NOT READ]

1. Were you given antibiotics in the month before you first became ill?

Yes

No [GO TO QUESTION 9]

Unsure [GO TO QUESTION 9]

1. What antibiotics were you given and why?

[ENTER TEXT]

1. Have you ever been diagnosed with leptospirosis before this?

Yes

No [GO TO K10]

Unsure [GO TO K10]

1. How recent was that diagnosis? [READ OPTIONS]

In the last 2 years

Between 2 to 5 years ago

Over 5 years

**K10 QUESTIONS**

[THE FOLLOWING QUESTIONS MAY CAUSE DISTRESS TO THE PARTICIPANT. DO NOT GO TO NEXT QUESTION IF THE PARTICIPANT ANSWERS SOME OF THE TIME, MOST OF THE TIME OR ALL OF THE TIME FOR THE QUESTIONS. PERFORM RISK ASSESSMENT AND RISK MANAGEMENT AS PER THE PROTOCOL BEFORE PROCEEDING WITH THE QUESTIONS]

We have now come to the last 10 questions in the survey. Before we get onto them, I just want to see how you are doing… [WAIT FOR THE PARTICIPANT TO RESPOND]. In this set of questions, we will ask you about your wellbeing in relation to anxiety and depression. 1 in 3 people suffering from leptospirosis have long term symptoms including depression. This simple questions aims to check whether you may have been affected by depression and anxiety during the past four weeks. There is no right or wrong answer. Don’t linger too long over each question; usually your first response is the best. Are you ready to proceed?

[WAIT TO SEE IF THE PARTICIPANT HAS ANY QUESTION]

1. Thinking back to the last 30 days, about how often did you feel tired out for no good reason? [READ OPTIONS]

None of the time

A little of the time

Some of the time

Most of the time

All of the time

1. Thinking back to the last 30 days, about how often did you feel nervous? [READ OPTIONS]

None of the time

A little of the time

Some of the time

Most of the time

All of the time

1. Thinking back to the last 30 days, about how often did you feel so nervous that nothing could calm you down?

None of the time

A little of the time

Some of the time

Most of the time

All of the time

1. Thinking back to the last 30 days, about how often did you feel hopeless? [READ OPTIONS]

None of the time

A little of the time

Some of the time

Most of the time

All of the time

1. Thinking back to the last 30 days, about how often did you feel restless or fidgety? [READ OPTIONS]

None of the time

A little of the time

Some of the time

Most of the time

All of the time

1. Thinking back to the last 30 days, about how often did you feel so restless you could not sit still? [READ OPTIONS]

None of the time

A little of the time

Some of the time

Most of the time

All of the time

1. Thinking back to the last 30 days, about how often did you feel depressed? [READ OPTIONS]

None of the time

A little of the time

Some of the time

Most of the time

All of the time

1. Thinking back to the last 30 days, about how often did you feel that everything was an effort? [READ OPTIONS]

None of the time

A little of the time

Some of the time

Most of the time

All of the time

1. Thinking back to the last 30 days, about how often did you feel so sad that nothing could cheer you up? [READ OPTIONS]

None of the time

A little of the time

Some of the time

Most of the time

All of the time

1. Thinking back to the last 30 days, about how often did you feel worthless? [READ OPTIONS]

None of the time

A little of the time

Some of the time

Most of the time

All of the time

Thank you for participating in the survey. It is now complete. How are you feeling? Just a reminder that if you had any concerns or felt unease after this phone call, we have provided a list of support services in the information sheet for you to seek help. You can also talk to you doctor for any issues raised from this survey. Once again, thank you for your support. Your input in this research will enable us to develop appropriate strategies to support people with leptospirosis.

[IF PARTICIPANT GAVE CONSENT TO PARTICIPATE IN THE 6 MONTH FOLLOW-UP INTERVIEW, ASK THEM TO KEEP A DIARY OF ANY COSTS THEY INCUR DUE TO THEIR LEPTOSPIROSIS IN THE NEXT 6 MONTHS, ANY VISITS TO DOCTORS OR MEDICATION GIVEN, THE DURATION OF THEIR SYMTOMS IF THEY STILL HAVE SYMPTOMS AND ANY WORKPLACE COMPENSATION THEY HAVE RECEIVED].

Take care and bye for now.
