# Supplementary material for: Leptospirosis in Aotearoa New Zealand: Protocol for a Nationwide Case-Control Study
Source: JMIR Res Protoc. 2023 Jun 8;12:e47900. doi: 10.2196/47900 (PMC10288348; doi:10.2196/47900)
Supplement: Multimedia Appendix 9 [file resprot_v12i1e47900_app9.docx]

**Multimedia Appendix 9: Control questionnaire**

_______________________________________________________________________ Final 2

LEPTO Survey

________________________________________________________________________________

INTRO Hello, my name is %INAME% and I am calling you on behalf of Massey

University.

I would like to speak to %FNAM% %SNAM% [Person identified from

Database]- Is that you?

[If the correct person is on the telephone, go to Introduction.]-[If

it is not the correct person on the telephone, ask, can I please

speak to %FNAM% %SNAM%?

[When the correct person comes to the telephone - repeat greeting

and go to Introduction]

[If the correct person is not available, depending on quotas

required:

A) - Thank the person on the telephone and end the call, or..

B) - Arrange a time to call back when the named contact person is in.

SP

Proceed..........................................[] GO TO INTRO1

Stop interview, Make Appointment (ARRANGE CALL

BACK)............................................[]

Don't proceed....................................[]

________________________________________________________________________________

GOTOSMS

________________________________________________________________________________

INTRO1 Hello %FNAM%. I am calling you because you have been randomly

selected to take part in a control survey.

Have you received a letter containing information about this study?

Yes..............................................1

No...............................................2

________________________________________________________________________________

I2 Is this a good time to talk?

Yes..............................................1 GO TO SKIPI5

No - Make Appointment (ARRANGE CALL BACK)........2

________________________________________________________________________________

GOTOSMS

________________________________________________________________________________

SKIPI5 CONTINUE

________________________________________________________________________________

I5 Before we begin the survey, I need to confirm, are you 16 years or

older?

Yes..............................................1 GO TO SKIPQ6

No...............................................2

________________________________________________________________________________

IF I5='No' ASK

________________________________________________________________________________

I5TERM Sorry we are not able to include you in the study. Thank you for

your time. Do you have any questions for me %FNAM% before we

conclude?

Respondent Does Not Meet the Criteria for the

Survey...........................................1

________________________________________________________________________________

GOTOSMS

ASK ALL

________________________________________________________________________________

SKIPQ6 CONTINUE

I6 Have you had a flu-like illness in the last month?[DO NOT READ]

Yes..............................................1

No...............................................2 GO TO SKIPCON

________________________________________________________________________________

IF I6='Yes' ASK

________________________________________________________________________________

I6TERM Sorry we are not able to include you in the study. Thank you for

your time. Do you have any questions for me %FNAM% before we

conclude?

Respondent Does Not Meet the Criteria for the

Survey...........................................1

________________________________________________________________________________

GOTOSMS

ASK ALL

________________________________________________________________________________

SKIPCON CONTINUE

________________________________________________________________________________

In order to comply with the rules specified by the National Human Ethics

Committee and for your information, can we get your verbal consent if you wish

to participate in this study

[GO TO PISCF-4 WITH THEM FOR VERBAL CONSENT]

________________________________________________________________________________

VC1 [PISCF-4 COMPLETED WITH THEM FOR VERBAL CONSENT]

Do you consent for your answers to be used for research purposes

only?

Yes - I consent .................................1 GO TO SKIPVCY

Make Appointment (ARRANGE CALL BACK).............2

No - I dont consent my answers...................3

________________________________________________________________________________

GOTOSMS

________________________________________________________________________________

SKIPVCY CONTINUE

Thank you for consenting to this study. We will go through the survey questions

now. During this survey, I will ask you some questions that you may consider

sensitive. Please remember, it is your choice to answer these questions and we

can stop at any point.

I would like to confirm some details please

________________________________________________________________________________

C1 What is the name of your nearest primary school?

(18-37)

________________________________________________________________________________

C2 Do you live? [READ OPTIONS AND SELECT ONE]

In a town or city................................1

On a lifestyle block i.e. <10 acres..............2

On a farm........................................3

________________________________________________________________________________

I am going to ask two questions about your knowledge of leptospirosis. Your

answers will help us identify if there are knowledge gaps in society.

________________________________________________________________________________

C3 Do you know how you can get leptospirosis?

Yes..............................................1

No...............................................2

________________________________________________________________________________

IF C3='Yes' ASK

________________________________________________________________________________

C4 Can you give one example? [DO NOT READ]

Animal faeces....................................1

Animal urine.....................................2

Water............................................3

Soil.............................................4

Refused..........................................5

Other (specify) 6

(41-60)

Specified Other

________________________________________________________________________________

ASK ALL

________________________________________________________________________________

The next set of questions are more about you.

________________________________________________________________________________

D1 What is your date of birth?

Enter Date of Birth - FORMAT: DD/MM/YYYY^o.......1

[DO NOT READ] Refused............................2

Other (specify) 3

(8-27)

Specified Other

________________________________________________________________________________

IF D1='[DO NOT READ] Refused' ASK

________________________________________________________________________________

D1A What is your Age [IF REFUSED DATE OF BIRTH REQUEST AT D1]

0+ __________ (28-36)

Refused..........................................{

________________________________________________________________________________

IF D1A=REF ASK

________________________________________________________________________________

D1B What age group are you in?

Please stop me when I read out the appropriate age group.

[READ LIST]

Under 20.........................................1

20-29............................................2

30-39............................................3

40-49............................................4

50-59............................................5

60-69............................................6

70 Plus..........................................7

[DO NOT READ] Refused............................8

________________________________________________________________________________

ASK ALL

________________________________________________________________________________

D2 Which gender do you identify with? [READ OPTIONS]

Male.............................................1

Female...........................................2

Other............................................3

________________________________________________________________________________

D3 Which of the following ethnic groups do you belong to?[READ OPTIONS,

SELECT ALL THAT APPLY]

MP

NZ European......................................1

NZ Maori.........................................2

Samoan...........................................3

Cook Island Maori................................4

Tongan...........................................5

Nuiean...........................................6

Chinese..........................................7

Indian...........................................8

Other (specify) 9

(40-59)

Specified Other

________________________________________________________________________________

D4 What is your highest education level? [READ OPTIONS]

No education.....................................1

Primary..........................................2

Secondary........................................3

Tertiary education...............................4

[DO NOT READ] Refused............................5

________________________________________________________________________________

D5 Which statement best describes your yearly income (before tax) for

the last 12 months? [READ OPTIONS]

Zero income......................................1

$0 to $14,000....................................2

$14,001 to $48,000...............................3

$48,001 to $70,000...............................4

$70,001 and over.................................5

[DO NOT READ]Refuse to answer....................6

________________________________________________________________________________

D6 Over the past month what has been your job or occupation? If you had

more than one job, please tell me all of them.[THE STUDY IS

PARTICULARLY INTERESTED IN OCCUPATIONS THAT INVOLVE ANIMALS OR

OUTDOOR ENVIRONMENTS.

OCCUPATIONS OF THIS SORT SHOULD BE PROBED FOR A MORE SPECIFIC

DESCRIPTION. FOR EXAMPLE, IF THE JOB TITLE IS FARMER ASK IF THEY ARE

DAIRY FARMER, BEEF FARMER, SHEEP FARMER, CROP FARMER, MIXED FARMING

OR OTHER TYPES]

MP

Job title 1:^o...................................1

Job title 2:^o...................................2

Job title 3:^o...................................3

not working / retired............................4

Other (specify) 5

(8-27)

Specified Other

________________________________________________________________________________

D7 Did you do any other work e.g. part-time work, help a friend or work

outside in the past month?

Yes [SPECIFY JOBS]^o.............................1

No...............................................2

Unsure...........................................3

Other (specify) 4

(29-48)

Specified Other

________________________________________________________________________________

Throughout the questionnaire most of the questions I ask will be about what you

have done only in the past month.

I will now ask you about what contact with animals you may have had in the past

month.

________________________________________________________________________________

A1 Did you come in direct contact with any of the following livestock

or farmed animals or their urine in the past month? [READ OPTIONS

AND SELECT ALL THAT APPLY]

Dairy Beef

cattle cattle Sheep Pigs Goats Deer Alpacas

(50) (51) (52) (53) (54) (55) (56) SP

Yes............... 1 1 1 1 1 1 1

No................ 2 2 2 2 2 2 2

Unsure............ 3 3 3 3 3 3 3

Any

others-

Horses specify

(57) (58) SP

Yes............... 1 1

No................ 2 2

Unsure............ 3 3

________________________________________________________________________________

***** NEW QUESTION *****

________________________________________________________________________________

IF A1(1)='No' AND A1(2)='No' AND A1(3)='No' AND A1(4)='No' AND A1(5)='No'.AND

.A1(6)='No' AND A1(7)='No' AND A1(8)='No' AND A1(9)='No' GOTO SKIPA7

IF A1(9)='Yes' ASK

________________________________________________________________________________

A1OTH Please Specify

(59-78)

________________________________________________________________________________

ASK ALL

________________________________________________________________________________

A1A approximately how many days in that month, did you come in direct

contact with?..

Dairy Beef

cattle cattle Sheep Pigs Goats Deer Alpacas

4/8-16 (17-25) (26-34) (35-43) (44-52) (53-61) (62-70)

0+................ _______ _______ _______ _______ _______ _______ _______

Don't know........ Y Y Y Y Y Y Y

Any

others-

Horses specify

(71-79) 5/8-16

0+................ _______ _______

Don't know........ Y Y

________________________________________________________________________________

***** NEW QUESTION *****

________________________________________________________________________________

IF A1(9)='Yes' ASK

________________________________________________________________________________

A1AOTH approximately how many days in that month, did you come in direct

contact with [A1 YES RESPONSE]?-[.....]

0+ __________ (17-25)

Don't know.......................................Y

________________________________________________________________________________

ASK ALL

________________________________________________________________________________

A1B was direct contact with .... at work, outside of paid work or both?

Dairy Beef

cattle cattle Sheep Pigs Goats Deer Alpacas

(26) (27) (28) (29) (30) (31) (32) SP

At work........... 1 1 1 1 1 1 1

Outside of paid

work.............. 2 2 2 2 2 2 2

Both.............. 3 3 3 3 3 3 3

Unsure............ 4 4 4 4 4 4 4

Any

others-

Horses specify

(33) (34) SP

At work........... 1 1

Outside of paid

work.............. 2 2

Both.............. 3 3

Unsure............ 4 4

________________________________________________________________________________

***** NEW QUESTION *****

________________________________________________________________________________

IF A1(9)='YES' ASK

________________________________________________________________________________

A1BOTH was direct contact with [A1 YES RESPONSE]-[.....] at work, outside

of paid work or both?

At work..........................................1

Outside of paid work.............................2

Both.............................................3

Unsure...........................................4

________________________________________________________________________________

ASK ALL

________________________________________________________________________________

A2X Were you involved in any of the following livestock associated

activities in the past month? [READ OPTIONS AND SELECT ALL THAT

APPLY]

-1- Assisting in the calving of beef cattle

-2- Docking/castrating calves of beef cattle

-3- Drenching beef cattle

-4- Assisting in the calving of dairy cattle

-5- Milking dairy cows

-6- Docking/castrating calves of dairy cattle

-7- Drenching dairy cattle

-8- Assisting lambing

-9- Crutching or dagging sheep

-10- Shearing sheep

-11- Docking/castrating lambs

-12- Palpating udders of ewes

-13- Milking sheep

-14- Drenching sheep

-15- Cleaning urine or faeces from yard surface

-16- Home kill

-17- Killing for animal welfare

-18- Slaughtering animal

-19- Dressing animal carcass

-20- Any other livestock associated activities

-15- -16- -17- -18- -19- -20-

(9) (10) (11) (12) (13) (14) SP

Yes............... 1 1 1 1 1 1

No................ 2 2 2 2 2 2

Unsure............ 3 3 3 3 3 3

________________________________________________________________________________

***** NEW QUESTION *****

________________________________________________________________________________

IF A2X(20)='Yes' ASK

________________________________________________________________________________

A2XOTH Please specify any other?

(15-34)

________________________________________________________________________________

ASK ALL

________________________________________________________________________________

A2XA approximately how many days in that month were you involved with ....

-1- Assisting in the calving of beef cattle

-2- Docking/castrating calves of beef cattle

-3- Drenching beef cattle

-4- Assisting in the calving of dairy cattle

-5- Milking dairy cows

-6- Docking/castrating calves of dairy cattle

-7- Drenching dairy cattle

-8- Assisting lambing

-9- Crutching or dagging sheep

-10- Shearing sheep

-11- Docking/castrating lambs

-12- Palpating udders of ewes

-13- Milking sheep

-14- Drenching sheep

-15- Cleaning urine or faeces from yard surface

-16- Home kill

-17- Killing for animal welfare

-18- Slaughtering animal

-19- Dressing animal carcass

-20- Any other livestock associated activities

-15- -16- -17- -18- -19- -20-

(17-25) (26-34) (35-43) (44-52) (53-61) (62-70)

0+................ _______ _______ _______ _______ _______ _______

Don't know........ Y Y Y Y Y Y

________________________________________________________________________________

***** NEW QUESTION *****

________________________________________________________________________________

IF A2X(20)='Yes' ASK

________________________________________________________________________________

A2XAOTH approximately how many days in that month were you involved with

[.....]

0+ __________ (71-79)

Don't know.......................................Y

________________________________________________________________________________

ASK ALL

________________________________________________________________________________

A2XB was .... at work, outside of paid work or both?

-1- Assisting in the calving of beef cattle

-2- Docking/castrating calves of beef cattle

-3- Drenching beef cattle

-4- Assisting in the calving of dairy cattle

-5- Milking dairy cows

-6- Docking/castrating calves of dairy cattle

-7- Drenching dairy cattle

-8- Assisting lambing

-9- Crutching or dagging sheep

-10- Shearing sheep

-11- Docking/castrating lambs

-12- Palpating udders of ewes

-13- Milking sheep

-14- Drenching sheep

-15- Cleaning urine or faeces from yard surface

-16- Home kill

-17- Killing for animal welfare

-18- Slaughtering animal

-19- Dressing animal carcass

-20- Any other livestock associated activities

-15- -16- -17- -18- -19- -20-

(21) (22) (23) (24) (25) (26) SP

At work........... 1 1 1 1 1 1

Outside of paid

work.............. 2 2 2 2 2 2

Both.............. 3 3 3 3 3 3

Unsure............ 4 4 4 4 4 4

________________________________________________________________________________

***** NEW QUESTION *****

________________________________________________________________________________

IF A2X(20)='Yes' ASK

________________________________________________________________________________

A2XBOTH was [.....] at work, outside of paid work or both?

At work..........................................1

Outside of paid work.............................2

Both.............................................3

Unsure...........................................4

________________________________________________________________________________

ASK ALL

________________________________________________________________________________

A2XC When you were involved in ...., were the animals, fully vaccinated,

partially vaccinated or not vaccinated at all against leptospirosis?

-1- Assisting in the calving of beef cattle

-2- Docking/castrating calves of beef cattle

-3- Drenching beef cattle

-4- Assisting in the calving of dairy cattle

-5- Milking dairy cows

-6- Docking/castrating calves of dairy cattle

-7- Drenching dairy cattle

-8- Assisting lambing

-9- Crutching or dagging sheep

-10- Shearing sheep

-11- Docking/castrating lambs

-12- Palpating udders of ewes

-13- Milking sheep

-14- Drenching sheep

-15- Cleaning urine or faeces from yard surface

-16- Home kill

-17- Killing for animal welfare

-18- Slaughtering animal

-19- Dressing animal carcass

-20- Any other livestock associated activities

-15- -16- -17- -18- -19- -20-

(42) (43) (44) (45) (46) (47) SP

Fully vaccinated.. 1 1 1 1 1 1

Partially

vaccinated........ 2 2 2 2 2 2

Not vaccinated at

all............... 3 3 3 3 3 3

[DO NOT

READ]Unsure....... 4 4 4 4 4 4

________________________________________________________________________________

***** NEW QUESTION *****

________________________________________________________________________________

IF A2X(20)='Yes' ASK

________________________________________________________________________________

A2XCOTH When you were involved in ....., were the animals, fully vaccinated,

partially vaccinated or not vaccinated at all?

Fully vaccinated.................................1

Partially vaccinated.............................2

Not vaccinated at all............................3

[DO NOT READ]Unsure..............................4

________________________________________________________________________________

IF A2X(15)='Yes' ASK

________________________________________________________________________________

A2XD15 Earlier you indicated that you cleaned urine or faeces from a yard

surface - what type of animal or animals were involved?[VERBATIM

RESPONSE]]

(49-68)

________________________________________________________________________________

IF A2X(16)='Yes' ASK

________________________________________________________________________________

A2XD16 Earlier you indicated that you were involved in Home kill activity -

what type of animal or animals were involved? [VERBATIM RESPONSE]

(8-27)

________________________________________________________________________________

IF A2X(17)='Yes' ASK

________________________________________________________________________________

A2XD17 Earlier you indicated that you were involved in Killing for animal

welfare - what type of animal or animals were involved? [VERBATIM

RESPONSE]

(28-47)

________________________________________________________________________________

IF A2X(18)='Yes' ASK

________________________________________________________________________________

A2XD18 Earlier you indicated that you were involved in Slaughtering

animal(s) - what type of animal or animals were involved? [VERBATIM

RESPONSE]

(48-67)

________________________________________________________________________________

IF A2X(19)='Yes' ASK

________________________________________________________________________________

A2XD19 Earlier you indicated that you were involved in Dressing animal

carcass(es) - what type of animal or animals were involved?

[VERBATIM RESPONSE]

(8-27)

________________________________________________________________________________

ASK ALL

________________________________________________________________________________

A3 Have you had any contact with any animals that have been newly

introduced to a farm in the past month?

Yes..............................................1

No...............................................2 GO TO SKIPA5

Unsure...........................................3 GO TO SKIPA5

________________________________________________________________________________

A4 Were the introduced animals fully vaccinated, partially vaccinated

or not vaccinated at all?

Fully vaccinated.................................1

Partially vaccinated.............................2

Not vaccinated at all............................3

[DO NOT READ]Unsure..............................4

________________________________________________________________________________

SKIPA5 CONTINUE

IF A1(1)='No' AND A1(2)='No' AND A1(3)='No' AND A1(4)='No' AND A1(5)='No'.AND

.A1(6)='No' AND A1(7)='No' AND A1(8)='No' AND A1(9)='No' GOTO SKIPA7

ASK ALL

________________________________________________________________________________

A5X Generally, did you use any of the following personal protective

equipment while handling livestock in the past month?[READ

LIST]-[MULTI]

MP

Gloves...........................................1

Boots............................................2

Apron............................................3

Goggles..........................................4

Facemask.........................................5

Other (specify) 6

Unsure[DO NOT READ]..............................7

None[DO NOT READ]................................9

(8-27)

Specified Other

________________________________________________________________________________

A6 How often did you wash your hands with water and soap immediately

after handling livestock in the past month?

All the time.....................................1

Sometimes........................................2

Never............................................3

Other (specify) 4

(29-48)

Specified Other

________________________________________________________________________________

SKIPA7 CONTINUE

________________________________________________________________________________

I am also interested to know if you came across any wild animals

________________________________________________________________________________

A8 Did you come in direct contact with any of the following wild

animals in the past month?..

Wild Wild Wild Wild Feral Wild Wild

goats pigs deer rabbits cats rats mice

(73) (74) (75) (76) (77) (78) (79) SP

Yes............... 1 1 1 1 1 1 1

No................ 2 2 2 2 2 2 2

Unsure............ 3 3 3 3 3 3 3

Ferret/

Stoat/ He Any

Weasel Possums dgehogs others

(80) 10/8 (9) (10) SP

Yes............... 1 1 1 1

No................ 2 2 2 2

Unsure............ 3 3 3 3

________________________________________________________________________________

***** NEW QUESTION *****

________________________________________________________________________________

IF A8(11)='Yes' ASK

________________________________________________________________________________

A8OTH Please specify any others:[PROMPT FOR ONE ANIMAL THEY HAD MOST

DIRECT CONTACT WITH]

(11-30)

________________________________________________________________________________

ASK ALL

IF A8(1)='Yes' OR A8(2)='Yes' OR A8(3)='Yes' OR A8(4)='Yes' OR A8(5)='Yes'.OR

.A8(6)='Yes' OR A8(7)='Yes' OR A8(8)='Yes' OR A8(9)='Yes' OR A8(10)='Yes' ASK

________________________________________________________________________________

A8A approximately how many days in that month did you come in direct

contact with ......

Wild Wild Wild Wild Feral Wild Wild

goats pigs deer rabbits cats rats mice

(31-39) (40-48) (49-57) (58-66) (67-75) 11/8-16 (17-25)

0+................ _______ _______ _______ _______ _______ _______ _______

Don't know........ Y Y Y Y Y Y Y

Ferret/

Stoat/ He Any

Weasel Possums dgehogs others

(26-34) (35-43) (44-52) (53-61)

0+................ _______ _______ _______ _______

Don't know........ Y Y Y Y

________________________________________________________________________________

***** NEW QUESTION *****

________________________________________________________________________________

IF A8(11)='Yes' ASK

________________________________________________________________________________

A8AOTH approximately how many days in that month did you come in direct

contact with [.....]

0+ __________ (62-70)

Don't know.......................................Y

________________________________________________________________________________

ASK ALL

________________________________________________________________________________

A8B was the contact with .... at work, outside of paid work or both?

Wild Wild Wild Wild Feral Wild Wild

goats pigs deer rabbits cats rats mice

(71) (72) (73) (74) (75) (76) (77) SP

At work........... 1 1 1 1 1 1 1

Outside of paid

work.............. 2 2 2 2 2 2 2

Both.............. 3 3 3 3 3 3 3

Unsure............ 4 4 4 4 4 4 4

Ferret/

Stoat/ He Any

Weasel Possums dgehogs others

(78) (79) (80) 12/8 SP

At work........... 1 1 1 1

Outside of paid

work.............. 2 2 2 2

Both.............. 3 3 3 3

Unsure............ 4 4 4 4

________________________________________________________________________________

***** NEW QUESTION *****

________________________________________________________________________________

IF A8(11)='Yes' ASK

________________________________________________________________________________

A8BOTH was the contact with ..... at work, outside of paid work or both?

At work..........................................1

Outside of paid work.............................2

Both.............................................3

Unsure...........................................4

________________________________________________________________________________

ASK ALL

________________________________________________________________________________

ASK ALL

________________________________________________________________________________

A7X Did you notice any evidence, for example, droppings, gnawed feed

bags or fruit damage etc in the past month of the following types of

animals? [READ LIST]-[SELECT ALL THAT APPLY]

MP

Wild goats.......................................1

Wild deer........................................2

Feral cats.......................................3

Wild mice........................................4

Possums..........................................5

Wild pigs........................................6

Wild rabbits.....................................7

Wild rats........................................8

Ferret/Stoat/Weasel..............................9

Hedgehogs........................................0

None.............................................1

Other (specify) 2

(30-49)

Specified Other

________________________________________________________________________________

A9X Did you set any traps/poison for any of the following animals in the

past month?..

Ferret/

Wild Wild Wild Feral Wild Wild Stoat/

deer goats rabbits cats rats mice Weasel

(30) (31) (32) (33) (34) (35) (36) SP

Yes............... 1 1 1 1 1 1 1

No................ 2 2 2 2 2 2 2

Unsure............ 3 3 3 3 3 3 3

________________________________________________________________________________

He Any

Possums dgehogs others

(37) (38) (39) SP

Yes............... 1 1 1

No................ 2 2 2

Unsure............ 3 3 3

________________________________________________________________________________

***** NEW QUESTION *****

________________________________________________________________________________

IF A9X(10)='Yes' ASK

________________________________________________________________________________

A9XOTH Please specify any others:[PROMPT FOR ONE ANIMAL THEY HAD MOST

DIRECT CONTACT WITH]

(40-59)

________________________________________________________________________________

ASK ALL

IF A9X(1)='Yes' OR A9X(2)='Yes' OR A9X(3)='Yes' OR A9X(4)='Yes' OR A9X(5)='Yes'

OR A9X(6)='Yes' OR A9X(7)='Yes' OR A9X(8)='Yes' OR A9X(9)='Yes' OR A9X(10)=

'Yes' ASK

________________________________________________________________________________

A9AX approximately how many days in that month did you set any

traps/poison for ....

Ferret/

Wild Wild Wild Feral Wild Wild Stoat/

deer goats rabbits cats rats mice Weasel

(60-68) (69-77) 49/8-16 (17-25) (26-34) (35-43) (44-52)

0+................ _______ _______ _______ _______ _______ _______ _______

Don't know........ Y Y Y Y Y Y Y

He Any

Possums dgehogs others

(53-61) (62-70) (71-79)

0+................ _______ _______ _______

Don't know........ Y Y Y

________________________________________________________________________________

***** NEW QUESTION *****

________________________________________________________________________________

IF A9X(10)='Yes' ASK

________________________________________________________________________________

A9AXOTH approximately how many days in that month did you set any

traps/poison for .....

0+ __________ (8-16)

Don't know.......................................Y

________________________________________________________________________________

ASK ALL

________________________________________________________________________________

A9BX was the traps/poison for .... at work, outside of paid work or both?

Ferret/

Wild Wild Wild Feral Wild Wild Stoat/

deer goats rabbits cats rats mice Weasel

(17) (18) (19) (20) (21) (22) (23) SP

At work........... 1 1 1 1 1 1 1

Outside of paid

work.............. 2 2 2 2 2 2 2

Both.............. 3 3 3 3 3 3 3

Unsure............ 4 4 4 4 4 4 4

He Any

Possums dgehogs others

(24) (25) (26) SP

At work........... 1 1 1

Outside of paid

work.............. 2 2 2

Both.............. 3 3 3

Unsure............ 4 4 4

________________________________________________________________________________

***** NEW QUESTION *****

________________________________________________________________________________

IF A9X(10)='Yes' ASK

________________________________________________________________________________

A9XBOTH was the traps/poison for ..... at work, outside of paid work or both?

At work..........................................1

Outside of paid work.............................2

Both.............................................3

Unsure...........................................4

________________________________________________________________________________

ASK ALL

________________________________________________________________________________

A10X Did you empty any traps for any of the following animals in the past

month?..

Ferret/

Wild Wild Wild Feral Wild Wild Stoat/

deer goats rabbits cats rats mice Weasel

(30) (31) (32) (33) (34) (35) (36) SP

Yes............... 1 1 1 1 1 1 1

No................ 2 2 2 2 2 2 2

Unsure............ 3 3 3 3 3 3 3

He Any

Possums dgehogs others

(37) (38) (39) SP

Yes............... 1 1 1

No................ 2 2 2

Unsure............ 3 3 3

________________________________________________________________________________

***** NEW QUESTION *****

________________________________________________________________________________

IF A10X(10)='Yes' ASK

________________________________________________________________________________

A10XOTH Please specify any others:[PROMPT FOR ONE ANIMAL THEY HAD MOST

DIRECT CONTACT WITH]

(40-59)

________________________________________________________________________________

ASK ALL

IF A10X(1)='Yes' OR A10X(2)='Yes' OR A10X(3)='Yes' OR A10X(4)='Yes' OR A10X(5)=

'Yes' OR A10X(6)='Yes' OR A10X(7)='Yes' OR A10X(8)='Yes' OR A10X(9)='Yes'.OR

.A10X(10)='Yes' ASK

________________________________________________________________________________

A10AX approximately how many days in that month did you empty any traps

for ....

Ferret/

Wild Wild Wild Feral Wild Wild Stoat/

deer goats rabbits cats rats mice Weasel

(60-68) (69-77) 51/8-16 (17-25) (26-34) (35-43) (44-52)

0+................ _______ _______ _______ _______ _______ _______ _______

He Any

Possums dgehogs others

(53-61) (62-70) (71-79)

0+................ _______ _______ _______

________________________________________________________________________________

***** NEW QUESTION *****

________________________________________________________________________________

IF A10X(10)='Yes' ASK

________________________________________________________________________________

A10AXOTH approximately how many days in that month did you empty any traps

for .....

0+ __________ (8-16)

________________________________________________________________________________

ASK ALL

________________________________________________________________________________

A10BX was emptying the traps for .... at work, outside of paid work or

both?

Ferret/

Wild Wild Wild Feral Wild Wild Stoat/

deer goats rabbits cats rats mice Weasel

(17) (18) (19) (20) (21) (22) (23) SP

At work........... 1 1 1 1 1 1 1

Outside of paid

work.............. 2 2 2 2 2 2 2

Both.............. 3 3 3 3 3 3 3

Unsure............ 4 4 4 4 4 4 4

He Any

Possums dgehogs others

(24) (25) (26) SP

At work........... 1 1 1

Outside of paid

work.............. 2 2 2

Both.............. 3 3 3

Unsure............ 4 4 4

________________________________________________________________________________

***** NEW QUESTION *****

________________________________________________________________________________

ASK ALL

IF (A8(1)='No' AND A8(2)='No' AND A8(3)='No' AND A8(4)='No' AND A8(5)='No'.AND

.A8(6)='No' AND A8(7)='No' AND A8(8)='No' AND A8(9)='No' AND A8(10)='No' AND A8

(11)='No') AND (A9X(1)='No' AND A9X(2)='No' AND A9X(3)='No' AND A9X(4)='No'.AND

.A9X(5)='No' AND A9X(6)='No' AND A9X(7)='No' AND A9X(8)='No' AND A9X(9)='No'

AND A9X(10)='No') AND (A10X(1)='No' AND A10X(2)='No' AND A10X(3)='No' AND A10X

(4)='No' AND A10X(5)='No' AND A10X(6)='No' AND A10X(7)='No' AND A10X(8)='No'

AND A10X(9)='No' AND A10X(10)='No') GOTO SKIPA13

ASK ALL

________________________________________________________________________________

A11X Did you use any of the following personal protective equipment while

handling wildlife or traps in the past month?[READ LIST]-[SELECT ALL

THAT APPLY]

MP

Gloves...........................................1

Boots............................................2

Apron............................................3

Goggles..........................................4

Facemask.........................................5

Other (specify) 6

Unsure[DO NOT READ]..............................7

None[DO NOT READ]................................8

(51-70)

Specified Other

________________________________________________________________________________

A12 How often did you wash your hands immediately

after handling wildlife in the past month?

All the time.....................................1

Sometimes........................................2

Never............................................3

Other (specify) 4

(8-27)

Specified Other

________________________________________________________________________________

SKIPA13 CONTINUE

________________________________________________________________________________

Now I am going to ask you about any pet animals you may have come in contact

with.

________________________________________________________________________________

A13 Did you come in contact with any of the following pet animals in the

past month?

-1- Dogs (pets, farm dogs or service dogs)

-2- Cats

-3- Rats

-4- Mice

-5- Guinea Pigs

-6- Rabbits

-7- Any others

-1- -2- -3- -4- -5- -6- -7-

(29) (30) (31) (32) (33) (34) (35) SP

Yes............... 1 1 1 1 1 1 1

No................ 2 2 2 2 2 2 2

Unsure............ 3 3 3 3 3 3 3

________________________________________________________________________________

***** NEW QUESTION *****

________________________________________________________________________________

IF A13(7)='Yes' ASK

________________________________________________________________________________

A13OTH Please specify any others:[PROMPT FOR ONE ANIMAL THEY HAD MOST

DIRECT CONTACT WITH]

(36-55)

________________________________________________________________________________

ASK ALL

IF A13(1)='Yes' OR A13(2)='Yes' OR A13(3)='Yes' OR A13(4)='Yes' OR A13(5)='Yes'

OR A13(6)='Yes' OR A13(7)='Yes' ASK

________________________________________________________________________________

A13A approximately how many days in that month did you come in contact

with ....

-1- Dogs (pets, farm dogs or service dogs)

-2- Cats

-3- Rats

-4- Mice

-5- Guinea Pigs

-6- Rabbits

-7- Any others

-1- -2- -3- -4- -5- -6- -7-

(56-64) (65-73) 17/8-16 (17-25) (26-34) (35-43) (44-52)

0+................ _______ _______ _______ _______ _______ _______ _______

Don't know........ Y Y Y Y Y Y Y

________________________________________________________________________________

***** NEW QUESTION *****

________________________________________________________________________________

IF A13(7)='Yes' ASK

________________________________________________________________________________

A13AOTH approximately how many days in that month did you come in contact

with .....

0+ __________ (53-61)

Don't know.......................................Y

________________________________________________________________________________

ASK ALL

________________________________________________________________________________

A13B Was direct contact with .... at work, outside of paid work or both?

-1- Dogs (pets, farm dogs or service dogs)

-2- Cats

-3- Rats

-4- Mice

-5- Guinea Pigs

-6- Rabbits

-7- Any others

-1- -2- -3- -4- -5- -6- -7-

(62) (63) (64) (65) (66) (67) (68) SP

At work........... 1 1 1 1 1 1 1

Outside of paid

work.............. 2 2 2 2 2 2 2

Both.............. 3 3 3 3 3 3 3

Unsure............ 4 4 4 4 4 4 4

________________________________________________________________________________

***** NEW QUESTION *****

________________________________________________________________________________

IF A13(7)='Yes' ASK

________________________________________________________________________________

A13BOTH Was direct contact with ..... at work, was it outside of paid work

or both?

At work..........................................1

Outside of paid work.............................2

Both.............................................3

Unsure...........................................4

________________________________________________________________________________

ASK ALL

________________________________________________________________________________

A14 Did you clean up urine or feaces of any of the following pet animals?

-1- Dogs (pets, farm or service)

-2- Cats

-3- Rats

-4- Mice

-5- Guinea Pigs

-6- Rabbits

-7- Any others

-1- -2- -3- -4- -5- -6- -7-

(71) (72) (73) (74) (75) (76) (77) SP

Yes............... 1 1 1 1 1 1 1

No................ 2 2 2 2 2 2 2

Unsure............ 3 3 3 3 3 3 3

________________________________________________________________________________

***** NEW QUESTION *****

________________________________________________________________________________

IF A14(7)='Yes' ASK

________________________________________________________________________________

A14OTH Please specify any others:[PROMPT FOR ONE ANIMAL THEY HAD MOST

DIRECT CONTACT WITH]

(8-27)

________________________________________________________________________________

ASK ALL

IF A14(1)='Yes' OR A14(2)='Yes' OR A14(3)='Yes' OR A14(4)='Yes' OR A14(5)='Yes'

OR A14(6)='Yes' OR A14(7)='Yes' ASK

________________________________________________________________________________

A14A approximately how many days in that month did you clean up urine or

feaces of ....

-1- Dogs (pets, farm or service)

-2- Cats

-3- Rats

-4- Mice

-5- Guinea Pigs

-6- Rabbits

-7- Any others

-1- -2- -3- -4- -5- -6- -7-

(28-36) (37-45) (46-54) (55-63) (64-72) 19/8-16 (17-25)

0+................ _______ _______ _______ _______ _______ _______ _______

Don't know........ Y Y Y Y Y Y Y

________________________________________________________________________________

***** NEW QUESTION *****

________________________________________________________________________________

IF A14(7)='Yes' ASK

________________________________________________________________________________

A14AOTH approximately how many days in that month did you clean up urine or

feaces of .....

0+ __________ (26-34)

Don't know.......................................Y

________________________________________________________________________________

ASK ALL

________________________________________________________________________________

A14B did you clean up urine or feaces of .... at work, outside of paid

work or both?

-1- Dogs (pets, farm or service)

-2- Cats

-3- Rats

-4- Mice

-5- Guinea Pigs

-6- Rabbits

-7- Any others

-1- -2- -3- -4- -5- -6- -7-

(35) (36) (37) (38) (39) (40) (41) SP

At work........... 1 1 1 1 1 1 1

Outside of paid

work.............. 2 2 2 2 2 2 2

Both.............. 3 3 3 3 3 3 3

Unsure............ 4 4 4 4 4 4 4

________________________________________________________________________________

***** NEW QUESTION *****

________________________________________________________________________________

IF A14(7)='Yes' ASK

________________________________________________________________________________

A14BOTH did you clean up urine or feaces of ..... at work, outside of paid

work or both?

At work..........................................1

Outside of paid work.............................2

Both.............................................3

Unsure...........................................4

________________________________________________________________________________

ASK ALL

________________________________________________________________________________

Thinking back about all the animals that you had contact with including farmed

animals, wild animals or pets:

________________________________________________________________________________

A15 Did you hunt any animals in the past month?

Yes-[SPECIFY WHAT ANIMALS^o......................1

No...............................................2

Other (specify) 3

(44-63)

Specified Other

________________________________________________________________________________

A16 Were you involved with skinning or plucking animals in the past

month?

Yes-[SPECIFY WHAT ANIMALS^o......................1

No...............................................2

Other (specify) 3

(8-27)

Specified Other

________________________________________________________________________________

A17 Have you had contact with any dead animals in the past month that

were not slaughtered on purpose at home, work or elsewhere?

Yes-[SPECIFY WHAT ANIMALS^o......................1

No...............................................2

Unsure...........................................3

Other (specify) 4

(29-48)

Specified Other

________________________________________________________________________________

A18 Have you had contact with animals that aborted or have had still

births in the past month?

Yes-[SPECIFY WHAT ANIMALS^o......................1

No...............................................2

Unsure...........................................3

Other (specify) 4

(50-69)

Specified Other

________________________________________________________________________________

A19 Did you handle any animal feed in the past month?

Yes..............................................1

No...............................................2 GO TO SKIPW1

Unsure...........................................3 GO TO SKIPW1

________________________________________________________________________________

A19A Approximately, how many days in the past month did you handle animal

feed?

0+ __________ (71-79)

Don't know.......................................Y

________________________________________________________________________________

A19B Was handling the animal feed at work, outside of paid work or both?

At work..........................................1

Outside of paid work.............................2

Both.............................................3

Unsure...........................................4

________________________________________________________________________________

A19C Did you generally use gloves when handling the animal feed?

Yes..............................................1

No...............................................2

Unsure...........................................3

________________________________________________________________________________

A19D And what type of animal feed did you handle in the past month?

Yes..............................................1

No...............................................2

Unsure...........................................3

________________________________________________________________________________

SKIPW1 CONTINUE

________________________________________________________________________________

The following questions are about different environmental sources.

________________________________________________________________________________

W1 What are the sources of drinking water to your home?[READ

LIST]-{SELECT ALL THAT APPLY]

MP

Usual town supply................................1

Private bore / spring water......................2

Tanker truck water...............................3

Roof / rain water................................4

Creek / stream water.............................5

Other main source of drinking....................6

[DO NOT READ]Unsure..............................7

________________________________________________________________________________

IF W1='<Usual town supply>' ASK

________________________________________________________________________________

W1A Is the water from the usual town supply treated?[DO NOT READ]

Yes..............................................1

No...............................................2

Unsure...........................................3

________________________________________________________________________________

IF W1='<Private bore / spring water>' ASK

________________________________________________________________________________

W1B Is the water from the Private bore or spring treated?[DO NOT READ]

Yes..............................................1

No...............................................2

Unsure...........................................3

________________________________________________________________________________

IF W1='<Tanker truck water>' ASK

________________________________________________________________________________

W1C Is the water from the Tanker truck treated?[DO NOT READ]

Yes..............................................1

No...............................................2

Unsure...........................................3

________________________________________________________________________________

IF W1='<Roof / rain water>' ASK

________________________________________________________________________________

W1D Is the water collected from the roof treated?[DO NOT READ]

Yes..............................................1

No...............................................2

Unsure...........................................3

________________________________________________________________________________

IF W1='<Creek / stream water>' ASK

________________________________________________________________________________

W1E Is the water collected from the creek / stream treated?[DO NOT READ]

Yes..............................................1

No...............................................2

Unsure...........................................3

________________________________________________________________________________

IF W1='<Other main source of drinking>' ASK

________________________________________________________________________________

W1F Is the other main source of drinking water to your home treated?[DO

NOT READ]

Yes..............................................1

No...............................................2

Unsure...........................................3

________________________________________________________________________________

IF D6='<not working / retired>' ASK

GOTO SKIPW2

ASK ALL

________________________________________________________________________________

W2 What are the sources of drinking water to your place of employment?

[READ LIST]-[SELECT ALL THAT APPLY]

MP

Usual town supply................................1

Private bore / spring water......................2

Tanker truck water...............................3

Roof / rain water................................4

Creek / stream water.............................5

Other main source of drinking....................6

[DO NOT READ]Unsure..............................7

________________________________________________________________________________

IF W2='<Usual town supply>' ASK

________________________________________________________________________________

W2A Is the water from the usual town supply treated?[DO NOT READ]

Yes..............................................1

No...............................................2

Unsure...........................................3

________________________________________________________________________________

IF W2='<Private bore / spring water>' ASK

________________________________________________________________________________

W2B Is the water from the Private bore or spring treated?[DO NOT READ]

Yes..............................................1

No...............................................2

Unsure...........................................3

________________________________________________________________________________

IF W2='<Tanker truck water>' ASK

________________________________________________________________________________

W2C Is the water from the Tanker truck treated?[DO NOT READ]

Yes..............................................1

No...............................................2

Unsure...........................................3

________________________________________________________________________________

IF W2='<Roof / rain water>' ASK

________________________________________________________________________________

W2D Is the water collected from the roof treated?[DO NOT READ]

Yes..............................................1

No...............................................2

Unsure...........................................3

________________________________________________________________________________

IF W2='<Creek / stream water>' ASK

________________________________________________________________________________

W2E Is the water collected from the creek / stream treated?[DO NOT READ]

Yes..............................................1

No...............................................2

Unsure...........................................3

________________________________________________________________________________

IF W2='<Other main source of drinking>' ASK

________________________________________________________________________________

W2F Is the other main source of drinking water to your place of

employment treated?[DO NOT READ]

Yes..............................................1

No...............................................2

Unsure...........................................3

________________________________________________________________________________

ASK ALL

________________________________________________________________________________

SKIPW2 CONTINUE

________________________________________________________________________________

W4 Did you have contact with water from the following sources for

recreational purposes in the past month? [READ LIST]-[SELECT ALL

THAT APPLY]

MP

Ocean............................................1

River............................................2

Other (specify) 3

None.............................................4 GO TO W7SKIP

(24-43)

Specified Other

________________________________________________________________________________

W5 What sort of recreational activity were you involved with?[READ

LIST]-[SELECT ALL THAT APPLY]

....

Swimming Boating Fishing Any others

(45) (46) (47) (48) SP

Yes............... 1 1 1 1

No................ 2 2 2 2

Unsure............ 3 3 3 3

________________________________________________________________________________

***** NEW QUESTION *****

________________________________________________________________________________

IF W5(4)='Yes' ASK

________________________________________________________________________________

W5OTH Please specify any others

(49-68)

________________________________________________________________________________

IF W5(1)='Yes' OR W5(2)='Yes' OR W5(3)='Yes' OR W5(4)='Yes' ASK

________________________________________________________________________________

W6 And, in the past month, how many times a week did you go ....: More

than once a week, Once a week, or Less than once a week

Swimming Boating Fishing Any others

(69) (70) (71) (72) SP

More than once a

week.............. 1 1 1 1

Once a week....... 2 2 2 2

Less than once a

week.............. 3 3 3 3

[DO NOT

READ]Unsure....... 4 4 4 4

________________________________________________________________________________

***** NEW QUESTION *****

________________________________________________________________________________

IF W5(4)='Yes' ASK

________________________________________________________________________________

W6OTH And, in the past month, how many times a week did you go .....: More

than once a week, Once a week, or Less than once a week

More than once a week............................1

Once a week......................................2

Less than once a week............................3

[DO NOT READ]Unsure..............................4

________________________________________________________________________________

ASK ALL

W7SKIP CONTINUE

________________________________________________________________________________

W7X Did you encounter any of the following situations in the past

month?..

-1- Animal effluent

-2- Mud

-3- Drainage/plumbing work

-4- Landscaping

-5- Wetlands

-6- Flooding

-7- Any others[SPECIFY]

-1- -2- -3- -4- -5- -6- -7-

(72) (73) (74) (75) (76) (77) (78) SP

Yes............... 1 1 1 1 1 1 1

No................ 2 2 2 2 2 2 2

Unsure............ 3 3 3 3 3 3 3

________________________________________________________________________________

***** NEW QUESTION *****

________________________________________________________________________________

IF W7X(7)='Yes' ASK

________________________________________________________________________________

W7XOTH Please specify any others

(8-27)

________________________________________________________________________________

ASK ALL

IF W7X(1)='Yes' OR W7X(2)='Yes' OR W7X(3)='Yes' OR W7X(4)='Yes' OR W7X(5)='Yes'

OR W7X(6)='Yes' OR W7X(7)='Yes' ASK

________________________________________________________________________________

W7XA approximately how many days in that month did you encounter

-1- Animal effluent

-2- Mud

-3- Drainage/plumbing work

-4- Landscaping

-5- Wetlands

-6- Flooding

-7- Any others[SPECIFY]

-1- -2- -3- -4- -5- -6- -7-

(28-36) (37-45) (46-54) (55-63) (64-72) 42/8-16 (17-25)

0+................ _______ _______ _______ _______ _______ _______ _______

Don't know........ Y Y Y Y Y Y Y

________________________________________________________________________________

***** NEW QUESTION *****

________________________________________________________________________________

IF W7X(7)='Yes' ASK

________________________________________________________________________________

W7XAOTH approximately how many days in that month did you encounter .....

0+ __________ (26-34)

Don't know.......................................Y

________________________________________________________________________________

ASK ALL

________________________________________________________________________________

W7XB was the .... you encountered at work, outside of paid work or both?

-1- Animal effluent

-2- Mud

-3- Drainage/plumbing work

-4- Landscaping

-5- Wetlands

-6- Flooding

-7- Any others[SPECIFY]

-1- -2- -3- -4- -5- -6- -7-

(35) (36) (37) (38) (39) (40) (41) SP

At work........... 1 1 1 1 1 1 1

Outside of paid

work.............. 2 2 2 2 2 2 2

Both.............. 3 3 3 3 3 3 3

Unsure............ 4 4 4 4 4 4 4

________________________________________________________________________________

***** NEW QUESTION *****

________________________________________________________________________________

IF W7X(7)='Yes' ASK

________________________________________________________________________________

W7XBOTH was the ..... you encountered at work, outside of paid work or both?

At work..........................................1

Outside of paid work.............................2

Both.............................................3

Unsure...........................................4

________________________________________________________________________________

ASK ALL

________________________________________________________________________________

E1 Have you had any contact with soil for example gardening or

horticulture in the past month?[DO NOT READ]

Yes..............................................1

No...............................................2 GO TO SKIPE6

Unsure...........................................3 GO TO SKIPE6

________________________________________________________________________________

E2 Was this at work, outside of paid work or both?[DO NOT READ]

At work..........................................1

Outside of paid work.............................2

Both.............................................3

Unsure...........................................4

________________________________________________________________________________

E3 Did you use animal manure fertilizer while gardening or during

horticulture?[DO NOT READ]

Yes..............................................1

No...............................................2

Unsure...........................................3

________________________________________________________________________________

E4X Did you use any of the following personal protective equipment while

gardening or during horticulture?[READ LIST]

MP

Boots............................................1

Gloves...........................................2

Goggles..........................................3

Facemask.........................................4

Unsure[DO NOT READ]..............................5

None[DO NOT READ]................................6

Other (specify) 7

(44-63)

Specified Other

________________________________________________________________________________

E5 How often did you wash your hands immediately

after gardening in the past month?[READ LIST]

All the time.....................................1

Sometimes........................................2

Never............................................3

Other (specify) 4

(58-77)

Specified Other

________________________________________________________________________________

SKIPE6 CONTINUE

________________________________________________________________________________

E6 Have you drunk any raw milk in the past month?[DO NOT READ]

Yes..............................................1

No...............................................2

Unsure...........................................3

________________________________________________________________________________

E7 Have you taken part in other outdoor activities in the past

month?[READ LIST]

MP

Hiking/Walking...................................1

Camping..........................................2

Any others [SPECIFY]^o...........................3

No - none of above outdoor activities............4

Other (specify) 5

(8-27)

Specified Other

________________________________________________________________________________

E8 Have you walked barefoot outside in the past month?[DO NOT READ]

Yes..............................................1

No...............................................2

Unsure...........................................3

________________________________________________________________________________

SKIPE13 CONTINUE

________________________________________________________________________________

E13 Did you travel outside New Zealand in the past month?[DO NOT READ]

Yes..............................................1

No...............................................2 GO TO SKIPE14

Unsure...........................................3 GO TO SKIPE14

________________________________________________________________________________

Can you please provide some details of that travel outside of New Zealand?

________________________________________________________________________________

E14A Which country or countries did you visit?

(30-49)

Refused..........................................{

________________________________________________________________________________

E14B And what date did you leave New Zealand? [dd/mm/yy]

(50-69)

Refused..........................................{

________________________________________________________________________________

E14C And what date did you return to New Zealand?[dd/mm/yy]

(8-27)

Refused..........................................{

________________________________________________________________________________

SKIPE14 CONTINUE

________________________________________________________________________________

I will now ask you a few questions about your general health.

________________________________________________________________________________

H1 Have you had a cut or scratch in the past month[DO NOT READ]

Yes..............................................1

No...............................................2 GO TO SKIPH2

Unsure...........................................3 GO TO SKIPH2

________________________________________________________________________________

H2 Did you dress your wound?[DO NOT READ]

Yes..............................................1

No...............................................2

Unsure...........................................3

________________________________________________________________________________

SKIPH2 CONTINUE

________________________________________________________________________________

H3 Do you smoke cigarettes regularly, that is one or more a day?[DO NOT

READ]

Yes..............................................1 GO TO SKIPH5

No...............................................2

Refused..........................................3 GO TO SKIPH5

________________________________________________________________________________

H4 Have you ever been a regular smoker of one or more cigarettes a

day?[DO NOT READ]

Yes..............................................1

No...............................................2

Refused..........................................3

________________________________________________________________________________

SKIPH5 CONTINUE

________________________________________________________________________________

H5 Do you suffer from any of the following medical conditions?

Hay D Cardiac Lung Dep

fever Asthma iabetes disease disease Anxiety ression

(33) (34) (35) (36) (37) (38) (39) SP

Yes............... 1 1 1 1 1 1 1

No................ 2 2 2 2 2 2 2

Unsure............ 3 3 3 3 3 3 3

________________________________________________________________________________

***** NEW QUESTION *****

________________________________________________________________________________

IF H5(1)='Yes' OR H5(2)='Yes' OR H5(3)='Yes' OR H5(4)='Yes' OR H5(5)='Yes'.OR

.H5(6)='Yes' OR H5(7)='Yes' ASK

________________________________________________________________________________

H5A Can you tell me how many years you have had this condition for..

Hay D Cardiac Lung Dep

fever Asthma iabetes disease disease Anxiety ression

(40-48) (49-57) (58-66) (67-75) 26/8-16 (17-25) (26-34)

0+................ _______ _______ _______ _______ _______ _______ _______

Don't know........ Y Y Y Y Y Y Y

________________________________________________________________________________

***** NEW QUESTION *****

________________________________________________________________________________

ASK ALL

________________________________________________________________________________

H6 Are you on any regular medication?[DO NOT READ]

Yes [SPECIFY]^o..................................1

No...............................................2

Unsure...........................................3

Refused..........................................4

Other (specify) 5

(36-55)

Specified Other

________________________________________________________________________________

H7 Were you given antibiotics in the past month?[DO NOT READ]

Yes..............................................1

No...............................................2 GO TO SKIPH9

Unsure...........................................3 GO TO SKIPH9

________________________________________________________________________________

H8 What antibiotics were you given and why?

(57-76)

________________________________________________________________________________

SKIPH9 CONTINUE

________________________________________________________________________________

H9 Have you ever been diagnosed with lepto?[DO NOT READ]

Yes..............................................1

No...............................................2

Unsure...........................................3

________________________________________________________________________________

IF H9='Yes' ASK

________________________________________________________________________________

H10 How recent was this diagnosis?[READ LIST]

In the last 2 years..............................1

Between 2 to 5 years ago.........................2

Over 5 years.....................................3

[DO NOT READ] Unsure.............................4

________________________________________________________________________________

ASK ALL

________________________________________________________________________________

NAME Occasionally our supervisors call to ensure I have done the

interview.

May I have your first name only

________________________________________________________________________________

PHCHK ....and can I confirm that your phone number is %KEY%

Yes..............................................1

No...............................................2

________________________________________________________________________________

IF PHCHK='No' ASK

________________________________________________________________________________

PHONE ....and if you could tell me your phone number

[ENTER RESPONDENTS PHONE NUMBER]

(8-27)

________________________________________________________________________________

ASK ALL

________________________________________________________________________________

That's the end of this survey ......

We would like to thank you for taking part. My name is %INAME% and if you have

any queries about this survey you can contact:

Name: Dr Shahista Nisa

Phone: (06) 951 6918

Email: s.nisa-AT-massey.ac.nz
