# Supplementary material for: Leptospirosis in Aotearoa New Zealand: Protocol for a Nationwide Case-Control Study
Source: JMIR Res Protoc. 2023 Jun 8;12:e47900. doi: 10.2196/47900 (PMC10288348; doi:10.2196/47900)
Supplement: Multimedia Appendix 10 [file resprot_v12i1e47900_app10.docx]

**Multimedia Appendix 10: Follow-up questionnaire**

[COMMENTS WRITTEN IN SQUARE BRACKETS REPRESENTS NOTES FOR THE INTERVIEWER. IF HIGHLIGHTED IN YELLOW YOU NEED TO FIND INFO FROM THE ACUTE QUESTIONNAIRE]

Participant Name: _____________________________ Participant ID: _______

Acute Interviewer name: ________________ Acute interview date: ____

Follow-up Interviewer name: ______________ Follow-up interview date: _____

Support person name: _____________________ Support contact number: ____________

Hello, my name is Shahista Nisa and I am calling you from Massey University.

1. Is this PARTICIPANT NAME?

Yes [GO TO INTRODUCTION]

No

1. May I please speak with them?

Yes [GO TO INTRODUCTION]

No

1. When would be a good time to reach them?

_________________________ [ENTER TIME]

1. What is the best number to reach them?

___________________________ [ENTER NUMBER]

**SECTION 1: INTRODUCTION**

Hello PARTICIPANT NAME. Sha from Massey University spoke to you in MONTH/YEAR regarding your leptospirosis that you got in MONTH/YEAR. I am calling to see how you have been doing and do the follow up interview for lepto study. This survey will take about 20 minutes.

1. Is this a good time to talk?

Yes [GO TO CONSENT]

No

1. When would be a good time to reach you?

________________________ [ENTER TIME]

1. What is the best number to reach you?

________________________ [ENTER NUMBER]

**SECTION 2: CONSENT**

In order to comply with the rules specified by the Health and Disability Ethics Committee we need to ask for your verbal consent to use your answers for research purposes only. The answers you give in this survey will be used to create a de-identified dataset. This means your name is not included and no one will be able to identify you by looking at your answers. The dataset will be stored securely at Massey University. You can quit the study at any time, even after completing the questionnaire by contacting us. You may refuse to answer any questions that you would prefer not to answer. The findings from the study will be published in scientific reports, but these will not contain identifiable personal information. Information will be stored for 10 years before it is destroyed. If you have any concerns or questions, you can ask me now [PAUSE], or contact Shahista Nisa at Massey University on (06) 951 6918 or email at s.nisa@massey.ac.nz.

1. Do you consent for your answers to be used for research purposes only?

Yes

No [DO NOT PROCEED] Thank you for your time. This is the end of survey

**SECTION 3: IDENTIFICATION**

In case some of your details have changed since we last spoke, I would like to confirm these again please.

1. What is your address? [ENTER TEXT AND NUMBER]

NUMBER: _____________ STREET NAME: ___________________________________

SUBURB: ___________________________ TOWN:______________________________

POST CODE: _________

1. What is the best contact number for you?

[ENTER NUMBER]

1. What is your email address?

[ENTER TEXT]

1. What is the best way to contact you? [READ OPTIONS AND SELECT ALL THAT APPLIES]

Telephone

Email

Others [SPECIFY] _________

1. When is the best time to contact?

Anytime

Weekends [ASK FOR POSSIBLE TIME OPTIONS] _____________

Weekdays [ASK FOR POSSIBLE TIME OPTIONS] _______________

# **SECTION 4: UPDATE ON FIRST LEPTO SYMPTOMS**

In the first part of this survey, we will be asking you about some of the same topics we talked about last time. We want to know how you have been since we last spoke with you.

When you first became ill MONTH/YEAR you mentioned that you had the following symptoms. I will ask you to tell me more about these symptoms.

[SELECT FIRST ILL SYMPTOMS FROM ACUTE QUESTIONNAIRE (page 6 and 7) AND READ OUT SYMPTOMS IN “FIRST ILL SYMPTOMS” FROM TABLE BELOW. WHEN THE PATIENT FIRST BECAME ILL IS THE STARTING POINT. FOR EACH SYMPTOM ASK ABOUT DURATION, FREQUENCY AND SEVERITY USING A 5 POINT SCALE EACH TIME.]

| **Symptoms** | **Symptoms you reported when first ill with lepto** | | **How long did this affect you?**   1. **< a week** 2. **A week to a month** 3. **>1 to 3 months** 4. **>3 to 5 months** 5. **>6 months** | | **During that time, how frequent was this symptom?**   1. **Daily** 2. **2-3 times/week** 3. **weekly** 4. **monthly** 5. **Less than 5 times** | | **How severe was this symptom?**  **On a scale of 1-5 with 1 as mild and 5 as severe** | | **Do you still have this symptom?**  **Tick if yes** | |  |
| --- | --- | --- | --- | --- | --- | --- | --- | --- | --- | --- | --- |
| Headache |  | | 1 2 3 4 5 | | 1 2 3 4 5 | | 1 2 3 4 5 | |  | |  |
| Fever |  | | 1 2 3 4 5 | | 1 2 3 4 5 | | 1 2 3 4 5 | |  | |  |
| Sore eyes |  | | 1 2 3 4 5 | | 1 2 3 4 5 | | 1 2 3 4 5 | |  | |  |
| Sensitivity to light |  | | 1 2 3 4 5 | | 1 2 3 4 5 | | 1 2 3 4 5 | |  | |  |
| Muscle pain in your legs |  | | 1 2 3 4 5 | | 1 2 3 4 5 | | 1 2 3 4 5 | |  | |  |
| Muscle pain in your back |  | | 1 2 3 4 5 | | 1 2 3 4 5 | | 1 2 3 4 5 | |  | |  |
| Nausea |  | | 1 2 3 4 5 | | 1 2 3 4 5 | | 1 2 3 4 5 | |  | |  |
| Diarrhoea |  | | 1 2 3 4 5 | | 1 2 3 4 5 | | 1 2 3 4 5 | |  | |  |
| Abdominal pain |  | | 1 2 3 4 5 | | 1 2 3 4 5 | | 1 2 3 4 5 | |  | |  |
| Fatigue |  | | 1 2 3 4 5 | | 1 2 3 4 5 | | 1 2 3 4 5 | |  | |  |
| **Symptoms** | | **Symptoms you reported when first ill with lepto** | | **How long has this affected you?**   1. **< a week** 2. **A week to a month** 3. **>1 to 3 months** 4. **>3 to 5 months** 5. **>6 months** | | **During that time how frequent was the symptom?**   1. **Daily** 2. **2-3 times/week** 3. **weekly** 4. **monthly** 5. **Less than 5 times** | | **How severe was the symptom?**  **On a scale of 1-5 with 1 as mild and 5 as severe** | | **Do you still have this symptom?**  **Tick if yes** | |
| Cough | |  | | 1 2 3 4 5 | | 1 2 3 4 5 | | 1 2 3 4 5 | |  | |
| Additional symptoms reported when you first became ill [SPECIFY] | |  | |  | |  | |  | |  | |
|  | |  | | 1 2 3 4 5 | | 1 2 3 4 5 | | 1 2 3 4 5 | |  | |
|  | |  | | 1 2 3 4 5 | | 1 2 3 4 5 | | 1 2 3 4 5 | |  | |
|  | |  | | 1 2 3 4 5 | | 1 2 3 4 5 | | 1 2 3 4 5 | |  | |

**SECTION 5: SYMPTOMS DEVELOPED POST-FIRST LEPTO**

1. Since you first became ill with lepto have other symptoms developed that you feel are related to you having lepto?

Yes [FILL OUT TABLE BELOW]

No [GO TO SECTION 6]

| **Symptoms**  **[SPECIFY BELOW]** | **How long has this affected you?**   1. **< a week** 2. **A week to a month** 3. **>1 to 3 months** 4. **>3 to 5 months** 5. **>6 months** | **During that time how frequent was this symptom?**   1. **Daily** 2. **2-3 times/week** 3. **weekly** 4. **monthly** 5. **Less than 5 times** | **How severe was this symptom ?**  **On a scale of 1-5 with 1 as mild and 5 as severe** | **Do you still have this symptom?**  **Tick if yes** |
| --- | --- | --- | --- | --- |
|  | 1 2 3 4 5 | 1 2 3 4 5 | 1 2 3 4 5 |  |
|  | 1 2 3 4 5 | 1 2 3 4 5 | 1 2 3 4 5 |  |
|  | 1 2 3 4 5 | 1 2 3 4 5 | 1 2 3 4 5 |  |
|  | 1 2 3 4 5 | 1 2 3 4 5 | 1 2 3 4 5 |  |

# **SECTION 6: YOUR WORK LIFE**

[REVIEW TIME OFF FROM ACUTE QUESTIONNAIRE (page 5)]

We want to find out what impact lepto had on your work life please.

1. What is your job or occupation now? If you have more than one job, please tell me all of them.

[GET DETAIL: FOR EXAMPLE, IF THE JOB TITLE IS FARMER ASK IF THEY ARE DAIRY FARMER, BEEF FARMER, SHEEP FARMER, CROP FARMER, MIXED FARMING OR OTHER TYPES]

Used to be: [OCCUPATION]

Job title 1: __________________

Job title 2: ___________________

Job title 3: ____________________

Not working/retired: ______________

Other (specify): __________________

1. In MONTH/YEAR, you told us that you had taken [NUMBER OF DAYS OFF WORK] days of leave from work. Did you take any additional days off from your work or business that you believe was due to leptospirosis?

Yes

No [Go to QUESTION 4]

1. How many additional days did you take off work?

[NUMBER OF DAYS: ___________________________________________]

1. At anytime since you first became ill with lepto, did you get paid sick leave?

Yes

No

1. How well has your workplace supported your recovery? [READ OPTIONS]

1- very well

2- well

3- Neutral

4- not well

5- not very well at all

**SECTION 7: WORKPLACE COMPENSATION**

[REVIEW INITIAL QUESTIONNAIRE REGARDING WORKPLACE COMPENSATION (page 23-25) BEFORE PROCEEDING]

Thanks for that. I want to follow up on some questions about workplace compensation please. I will be repeating some questions from the previous interview to update how workplace compensation is going.

[REPEAT QUESTIONS 1-3 IF THE CLAIM WAS NOT LODGED OR ANSWERED UNSURE IN INITIAL QUESTIONNAIRE]

1. Were you eligible for Workers Compensation?

Yes

No [ASK FOR AND SPECIFY REASON WHY NO ___________________________________________________________ ]

Unsure

1. Was a claim lodged by your GP?

Yes [GO TO QUESTION 4]

No

Unsure

1. Do you know why a claim was not lodged?

Yes [SPECIFY REASON__________________________________________]

No [GO TO QUESTION 7]

[REPEAT QUESTION 4-9 IF THE CLAIM WAS LODGED BUT NOT ACCEPTED IN INITIAL QUESTIONNAIRE]

1. Was the claim accepted?

Yes [GO TO QUESTION 6]

No

Haven’t heard back [GO TO QUESTION 7]

1. Did you receive any feedback about why your claim was not accepted?

Yes [SPECIFY ________________________________ GO TO QUESTION 8]

No [GO TO QUESTION 8]

1. Did you receive any of the following entitlement?

Income replacement

Treatment and rehabilitation costs

Lump-sum compensation

Other [SPECIFY__________________________________________]

Unsure

None

1. How would you rate your compensation experience? [READ OPTIONS AND SELECT ONE]

Extremely satisfied

Satisfied

Dissatisfied

Extremely dissatisfied

1. Can you identify 3 areas that need improvement? [DO NOT READ OPTIONS]

Support from case managers

Communication

Timeliness

Others [SPECIFY_______________________________________________]

1. Can you identify 3 areas that you think went well during the process?

Support from case managers

Communication

Timeliness

Others [SPECIFY]

**SECTION 8: K10**

[THE FOLLOWING QUESTIONS MAY CAUSE DISTRESS TO THE PARTICIPANT. PERFORM RISK ASSESSMENT AND RISK MANAGEMENT AS PER PROTOCOL IF THE PARTICIPANT ANSWERS SOME OF THE TIME, MOST OF THE TIME OR ALL OF THE TIME FOR THE QUESTIONS.]

We have now to the 10 questions about mental wellness that you answered for us last time. Before we get onto them, I just want to see how you are doing… [WAIT FOR THE PARTICIPANT TO RESPOND]. There is no right or wrong answer. Don’t linger too long over each question; usually your first response is the best. Are you ready to proceed?

1. Thinking back to the last 30 days, about how often did you feel tired out for no good reason? [READ OPTIONS]

None of the time

A little of the time

Some of the time

Most of the time

All of the time

1. Thinking back to the last 30 days, about how often did you feel nervous? [READ OPTIONS]

None of the time

A little of the time

Some of the time

Most of the time

All of the time

1. Thinking back to the last 30 days, about how often did you feel so nervous that nothing could calm you down?

None of the time

A little of the time

Some of the time

Most of the time

All of the time

1. Thinking back to the last 30 days, about how often did you feel hopeless? [READ OPTIONS]

None of the time

A little of the time

Some of the time

Most of the time

All of the time

1. Thinking back to the last 30 days, about how often did you feel restless or fidgety? [READ OPTIONS]

None of the time

A little of the time

Some of the time

Most of the time

All of the time

1. Thinking back to the last 30 days, about how often did you feel so restless you could not sit still? [READ OPTIONS]

None of the time

A little of the time

Some of the time

Most of the time

All of the time

1. Thinking back to the last 30 days, about how often did you feel depressed? [READ OPTIONS]

None of the time

A little of the time

Some of the time

Most of the time

All of the time

1. Thinking back to the last 30 days, about how often did you feel that everything was an effort? [READ OPTIONS]

None of the time

A little of the time

Some of the time

Most of the time

All of the time

1. Thinking back to the last 30 days, about how often did you feel so sad that nothing could cheer you up? [READ OPTIONS]

None of the time

A little of the time

Some of the time

Most of the time

All of the time

1. Thinking back to the last 30 days, about how often did you feel worthless? [READ OPTIONS]

None of the time

A little of the time

Some of the time

Most of the time

All of the time

**SECTION 9: COST ASSOCIATED WITH LEPTOSPIROSIS**

Thanks for that. These questions are to work out if lepto has affected you financially. You may find it helpful to have your calendar with you.

1. How many times did you consult your doctor for your leptospirosis including when you first became ill in [MONTH, YEAR]?

[ENTER NUMBER___________________]

1. Were there any costs associated with the following in relation to you having lepto? [READ OPTIONS]

| **Activities** | **Cost involved** | **Approximate total spend** |
| --- | --- | --- |
| Doctor’s consultations |  | ____________ |
| Medicines |  | ____________ |
| Other health related e.g. physios |  | ____________ |
| Transport |  | ____________ |
| Childcare |  | ____________ |
| Time taken off work for any health care related visit (but not full days off work) |  | ____________ |
| Housework |  | ____________ |
| Gardening |  | ____________ |
| Other [SPECIFY________________] |  | ____________ |

1. On a scale of 1 to 5, has having lepto impacted on meeting your everyday needs for things like accommodation, food, clothing and other necessities? [READ OPTIONS]

1- Never

2- Rarely

3- Occasionally

4- Often

5- Always

**SECTION 10: YOUR SOCIAL AND FAMILY LIFE**

These questions are to work out if lepto has impacted your social and family life.

1. On a scale of 1 to 5, has having lepto restricted you from participating in any social or cultural events? [READ OPTIONS]

1- Never

2- Rarely

3- Occasionally

4- Often

5- Always

1. On a scale of 1 to 5, how well have you coped with your lepto journey? [READ OPTIONS]

1- very well

2- well

3- Neutral

4- not well

5- not very well at all

1. On a scale of 1 to 5, how well have you returned to your usual daily activities? [READ OPTIONS]

1- very well

2- well

3- Neutral

4- not well

5- not very well at all

1. On a scale of 1 to 5, how well has your family and friends supported your recovery? [READ OPTIONS]

1- very well

2- well

3- Neutral

4- not well

5- not very well at all

**SECTION 11: CLOSING**

Thank you very much for participating in this survey. Do you have anything else you would like to talk about, or any questions that you want to ask me [PAUSE, RESPOND AND NOTE DOWN THE QUESTION AND RESOLUTION

______________________________________________________________________________________________________________________________________________________
