# Supplementary material for: Leptospirosis in Aotearoa New Zealand: Protocol for a Nationwide Case-Control Study
Source: JMIR Res Protoc. 2023 Jun 8;12:e47900. doi: 10.2196/47900 (PMC10288348; doi:10.2196/47900)
Supplement: Multimedia Appendix 11 [file resprot_v12i1e47900_app11.docx]

**Multimedia Appendix 11: Semi-structured questionnaire**

*Text in italics represents notes for the interviewer*

Participant name: ____________________________________ Participant ID: ____________

Interviewer name: ___________________________________ Interview date: ___________

**INTRODUCTION**

*Formalities: intro, thanks. When ready and before you begin remind that a koha will be sent in the mail on completion of the interview.*

Thank you for taking time out for me today and agreeing to be a part of this study. Before we proceed, is it okay if I record our conversation?

Yes  No

*Start recording on zoom/Dictaphone/other and then repeat the recording request consent.*

I am keen to talk with you because we would like to hear about your experiences with leptospirosis. This will take approximately one hour. Please note that your personal details and any information you give will not be revealed to anyone other than the researchers.

The purpose of this research is to collect the experiences of people like you – who have experience with Lepto – as messages to improve the work of three groups of professionals:

1. Medical professionals (doctors, nurses)
2. Public health policymakers (in the Ministry of Health, District Health Boards, ACC)
3. Farm workers, meat workers

Before we begin, I need to ask you for verbal consent to use your answers for research purposes only.

- This conversation will be partially or wholly transcribed. We will send you a draft of this transcript and you can then change or delete text or add comments, before we place the transcript in an anonymised dataset.
- The conversation will be added to other people’s conversation to create a de-identified dataset. This means no one will be able to identify you by looking at your answers. The dataset will be stored securely at Massey University. You can withdraw from the study at any time by contacting us and we will destroy all your data.
- You may decline to discuss any topic you wish.
- The findings from the study will be published in scientific reports, but these will not contain identifiable personal information.
- Information will be stored for 10 years before it is destroyed.

Do you consent for your information to be used for research purposes only?

Yes  No

- *Read occupation and workplace compensation sections from acute and follow-up questionnaire to provide context to this interview.*
- *Go through sections 1 to 3.*
- *Use the risk assessment and risk management plans if necessary.*

*Let participant tell their story, prompt with bullet points below if necessary*

- *Why? How? Where? When? Who? What? Why? How much? How many?*
- *How does that make you feel? Why was that good/bad? How was that for you? What do you think went well? What didn’t go so well?*
- *Can you tell me anything else about that? Do you have any examples? Can you tell me more? Can you help me understand that [situation]?*
- *Look for unusual terms, strong annotations, flags*
- *Support if difficult: That must have been really hard for you. This seems like a hard thing to talk about, take your time (verbal and non-verbal support). Should we stop for a cup of tea and maybe come back to this?*
- *See specific prompts after each question.*

| **Why do we want to talk to you?**  The purpose of this research is to collect the experiences of people like you – who have experience with Lepto – as messages to improve the work of three groups of professionals:   1. Medical professionals (doctors, nurses) 2. Public health policymakers (in the Ministry of Health, District Health Boards, ACC) 3. Farm workers, meat workers   We believe the experiences of you and your family – and the experiences of others like you – can help reduce the infection rates and limit the negative effects on people’s health and livelihoods. We are grateful for your assistance. |
| --- |

**Section 1: Your illness (your messages to medical professionals)**

**Can you tell me about your health experience with leptospirosis?**

| Relevant notes from follow-up questionnaire for *this* person on *this* topic: |
| --- |

- *How are you feeling, eating, sleeping (mentally and physically)?*
- *Anything in particular that has hindered or helped you with dealing or living with physical effects of leptospirosis e.g. family, boss, neighbourhood, etc.*

**Section 2: Your livelihood (your messages to policymakers in government and ACC)**

**Can you tell me about how leptospirosis has affected you financially?**

| Relevant notes from follow-up questionnaire for *this* person on *this* topic: |
| --- |

- *Workplace compensation?*
- *Series of events that occurred. Easy or difficult process.*
- *If they sought cover, what entitlements received? If compensation not sought, why not?*
- *How did you get by?*
- *What were effects on other members of the household?*

**Section 3: Your advice to colleagues (your messages to other workers)**

**Given your experience with lepto, what kind of advice would you like to share or offer to other people such as workmates, but also employers or family members regarding lepto?**

| Relevant notes from follow-up questionnaire for *this* person on *this* topic: |
| --- |

- *Risks of infections*
- *Ways to protect from risk of infection*
- *Availability of information*
- *Change in views about lepto after illness*

**CONCLUSION**

- Is there anything else you would like to tell me?
- What outcomes would you like to see around leptospirosis in general?
- How has telling of your story been today?
- *If negative, do you need any support around this? Is there something I can do e.g. ring your GP and make an appointment?*

I have no more questions at this stage. Do you have anything else you would like to talk about, or any questions that you want to ask me?

Thank you so much for making time to see me today. We really value your trust and willingness to share your story. I will listen to the recording and start/read the transcription. If I have any questions regarding this interview, would it be ok to contact you?

Yes  No

Best time to contact: Anytime 9am-5pm 5pm-9pm Other (specify): _____

Best method of contact: ______________________________________________________

Contact details: _____________________________________________________________
